# Supplementary material for: N-[1,3-Dialkyl(aryl)-2-oxoimidazolidin-4-ylidene]-aryl(alkyl)sulphonamides as Novel Selective Human Cannabinoid Type 2 Receptor (hCB2R) Ligands; Insights into the Mechanism of Receptor Activation/Deactivation
Source: Molecules. 2022 Nov 23;27(23):8152. doi: 10.3390/molecules27238152 (PMC9738591; doi:10.3390/molecules27238152)
Supplement: Supplementary file 1 [file molecules-27-08152-s001.zip › molecules-2043356-supplementary.pdf]

Supporting Information

N-[1,3-Dialkyl(aryl)-2-oxoimidazolidin-4-ylidene]-aryl(alkyl)sulphonamides as novel selective human cannabinoid type 2 receptor (hCB2R) ligands. Insights into the mechanism of receptor activation/deactivation.

Eleonora Gianquinto[1], Federica Sodano[1,2], Barbara Rolando[1], Ali Mokhtar Mahmoud[3], Magdalena Kostrzewa[3], Marco Allarà[3], Poulami Kumar[3], Francesca Spyrakis[1], Alessia Ligresti[3,*], Konstantin Chegaev[1,*]

[1] Department of Drug Science and Technology, University of Torino, 10125 Torino, Italy;

[2] Department of Pharmacy, "Federico II" University of Naples, 80131 Naples, Italy;

[3] National Research Council of Italy, Institute of Biomolecular Chemistry, 80078 Pozzuoli, Italy

Corresponding authors: Alessia Ligresti, aligresti@icb.cnr.it ; Konstantin Chegaev, konstantin.chegaev@unito.it.

Table of contents

Figure S1. Competition radioligand binding assay at hCB2R. S2

Figure S2. Chemical structure of compounds 47, 49, 51, 53 from Mugnaini *et al.* S3

Figure S3. Docking poses of compound 47 and 49 in 5zty, and of 51 and 53 from Mugnaini *et al.* in 6kpc S4

Figure S4. Docking poses of compounds 10e and 10g in 6kpc S5

Figure S5. Docking poses of compound 12b in 5zty S6

Figure S6. Alignment of 12e and AM10257 in 5zty S7

^{1}H and ^{13}C NMR spectra of all final compounds S8-S50

Figure S1. Competition radioligand binding assay of **10d** (A), **10e** (B), **10f** (C), **10g** (D), **10h** (E), **12a** (F), **12b** (G), **12e** (H) and **13** (I) at hCB2R, using [^{3}H]CP55 940. Data are reported as mean ± SE of three independent experiments conducted in duplicate.

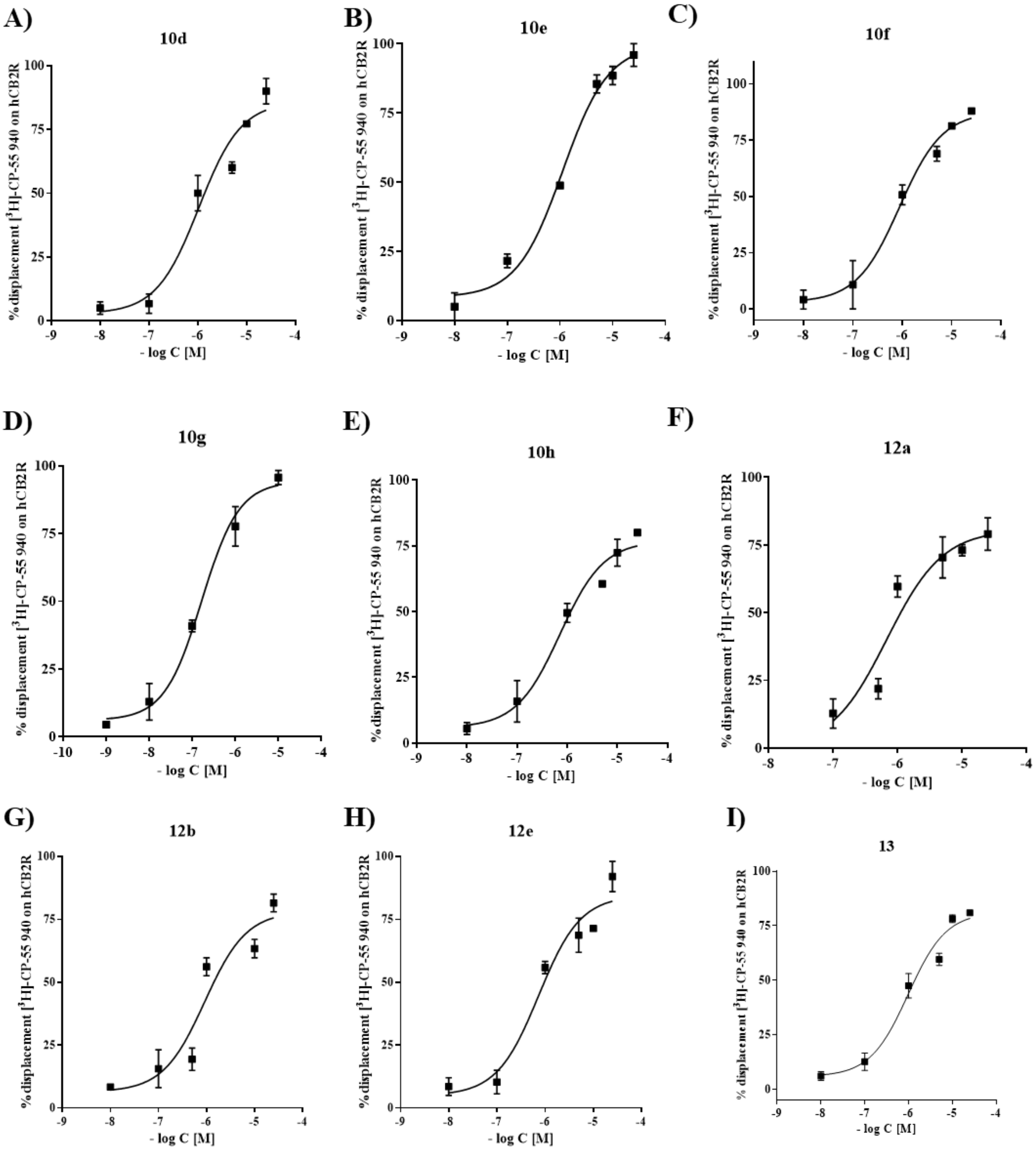

Figure S2. Chemical structure of compounds 47 (a), 49 (b), 51 (c) and 53 (d) from Mugnaini et al. [12].

a

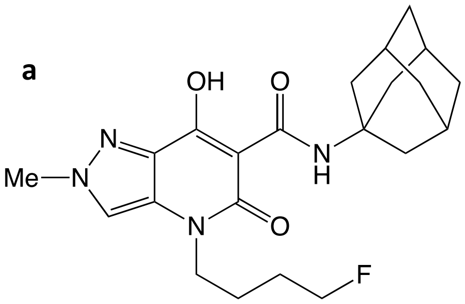

b

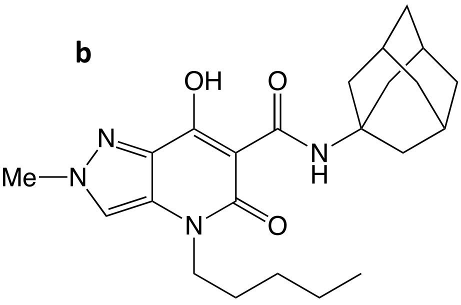

c

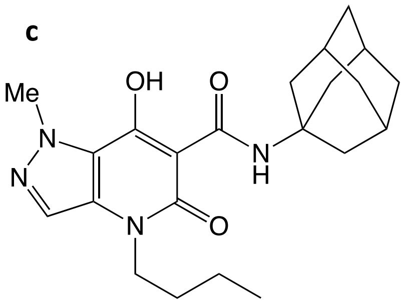

d

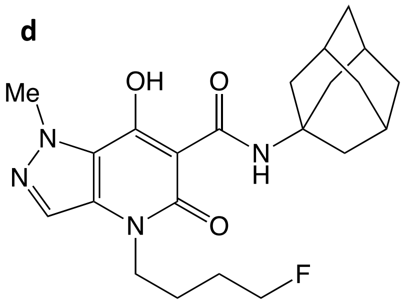

Figure S3. Docking poses of compound 49 (dark teal sticks, **a**) and compound 47 (grey sticks, **b**) in the antagonist conformation of hCB2R (PDB ID 5zty, light orange cartoon), and compound 51 (lilac sticks, **c**) and compound 53 (magenta sticks, **d**) in the agonist conformation of hCB2R (PDB ID 6kpc, light green cartoon). Compounds have been reported and described by Mugnaini et al. [12]. Residues lining the binding site are represented as capped sticks and labelled, helices are numbered as TM1-7.

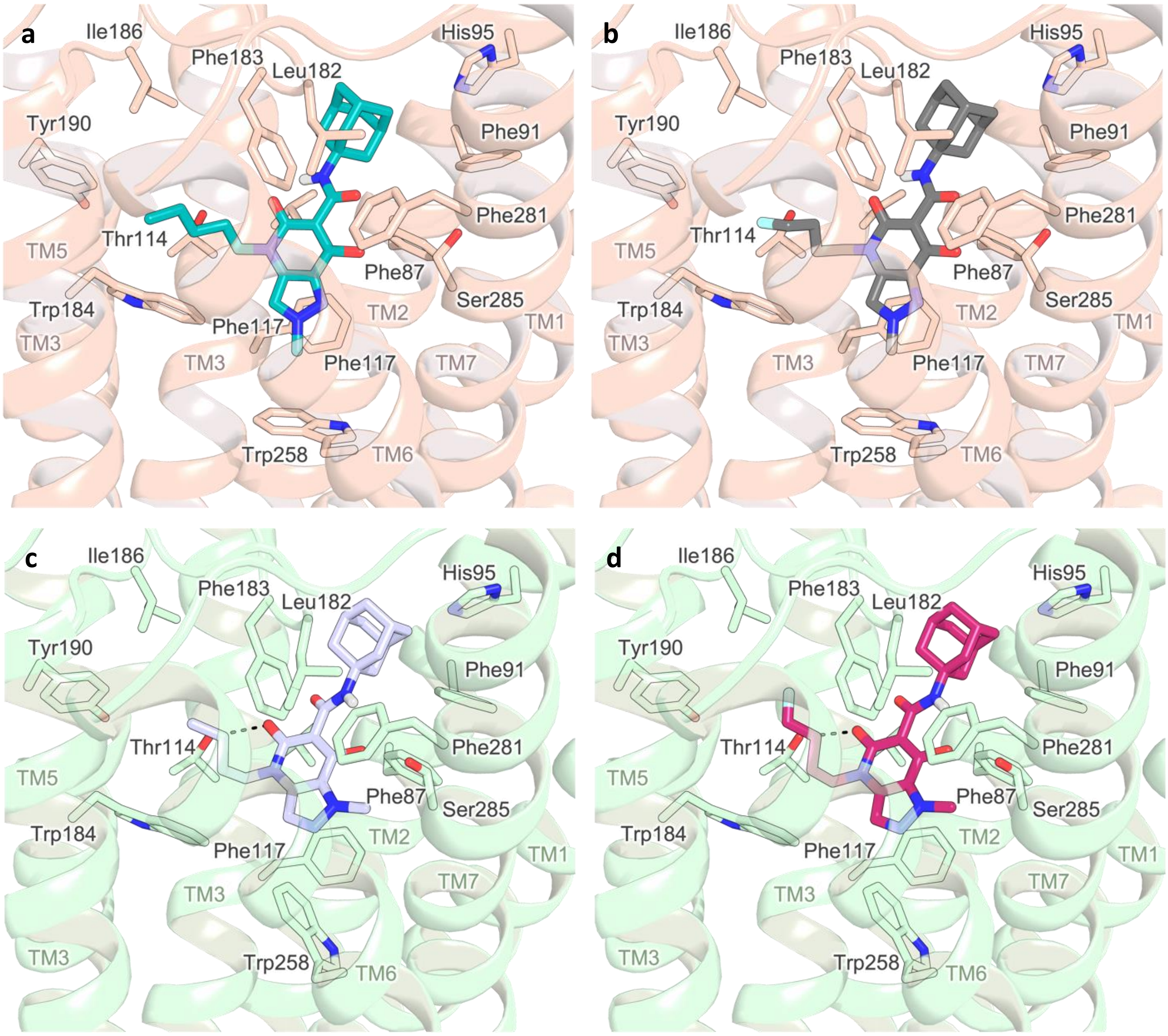

Figure S4. Docking poses of compounds **10e** (salmon pink, **a**) and **10g** (cyan, **b**) in the agonist conformation of hCB2R receptor (PDB ID: 6kpc, light green cartoon). Residues lining the binding site are represented as capped sticks and labelled, helices are numbered as TM1-7. Hydrogen bonds are represented as black dashed lines.

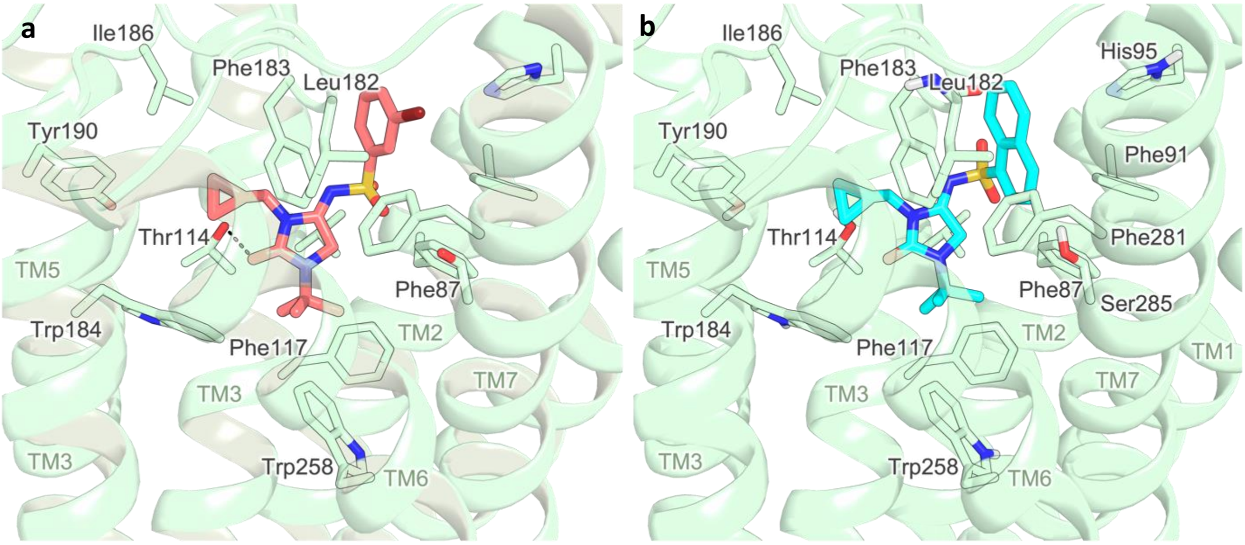

Figure S5. Docking poses of compound **12b** in the antagonist conformation of CB2R receptor (PDB ID 5zty). Residues lining the binding site are represented as capped sticks and labelled, transmembrane helices are numbered as TM1-7. Hydrogen bonds are represented as black dashed lines.

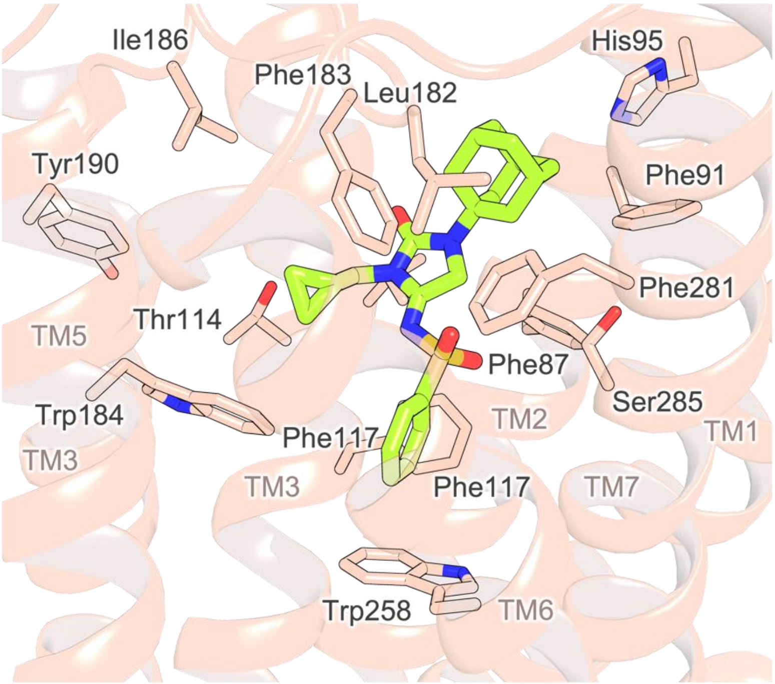

Figure S6. Alignment of **12e** docking pose (yellow) and antagonist AM10257 (green) in the antagonist-like state (PDB ID 5zty). Protein is coloured in light orange cartoon, residues lining the binding site are represented as capped sticks and labelled, helices are numbered as TM1-7.

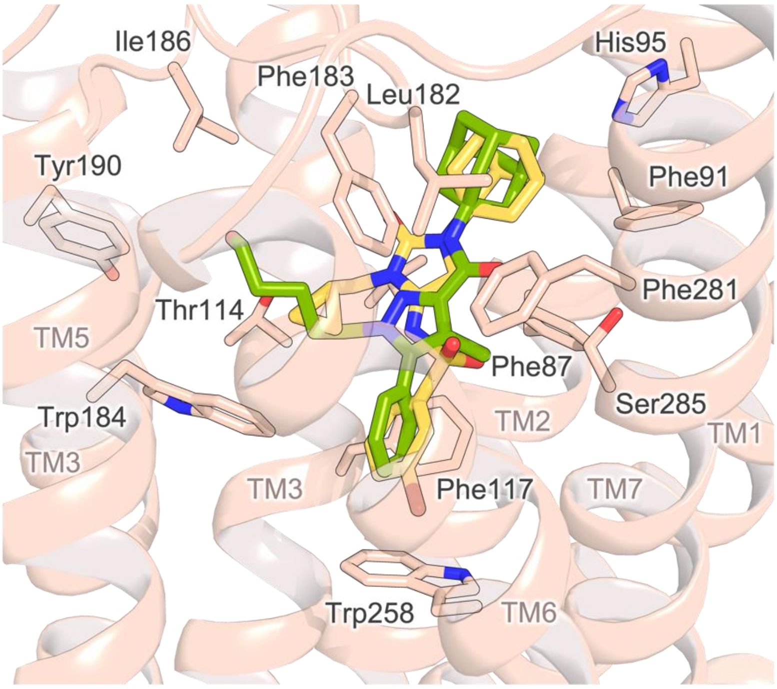

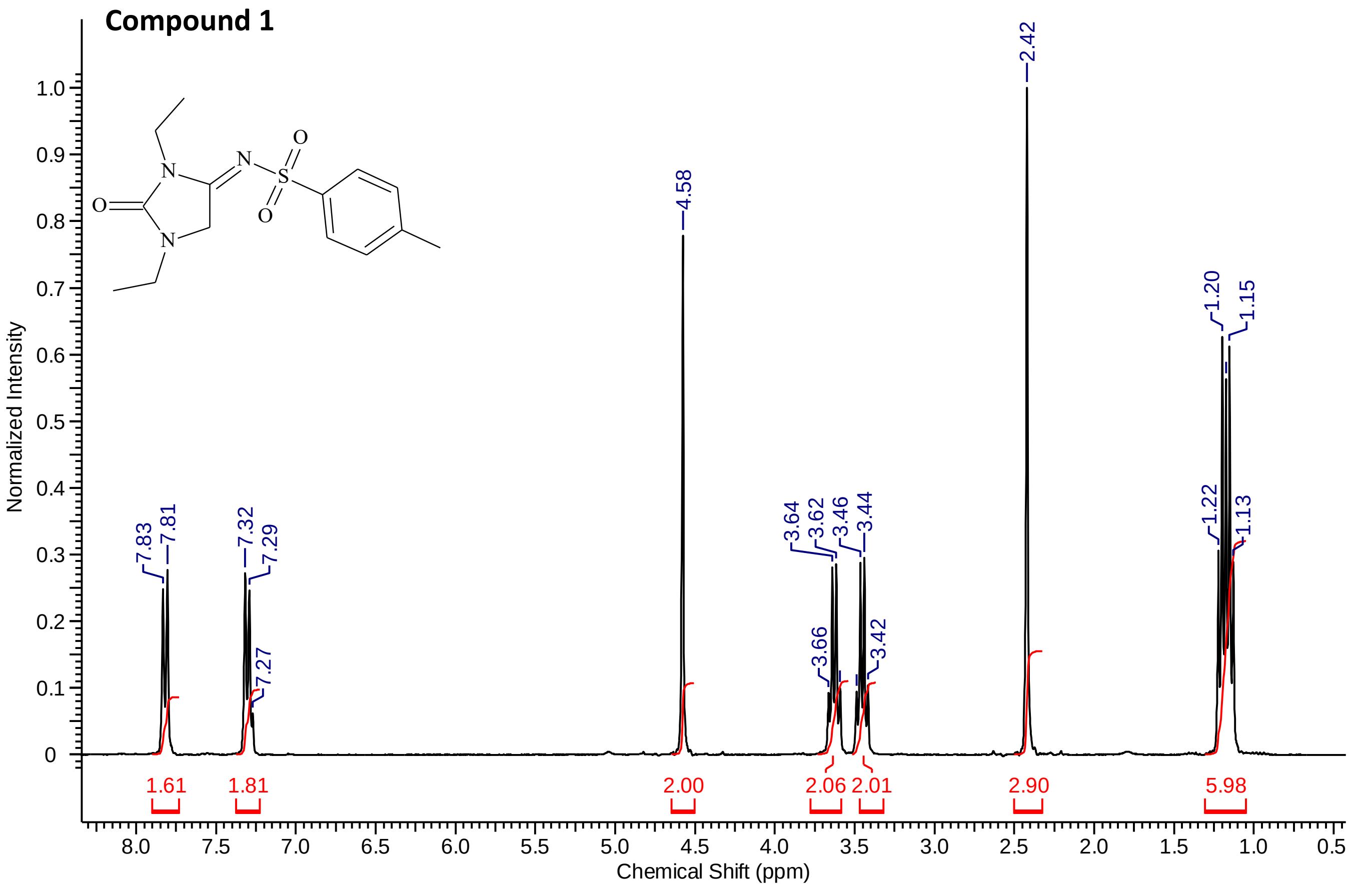
Compound 1
Normalized Intensity
Chemical Shift (ppm)
7.83 7.81 7.32 7.29 7.27 4.58 3.66 3.64 3.62 3.46 3.44 3.42 2.42 1.22 1.20 1.15 1.13
1.61 1.81 2.00 2.06 2.01 2.90 5.98
N N O N S O O

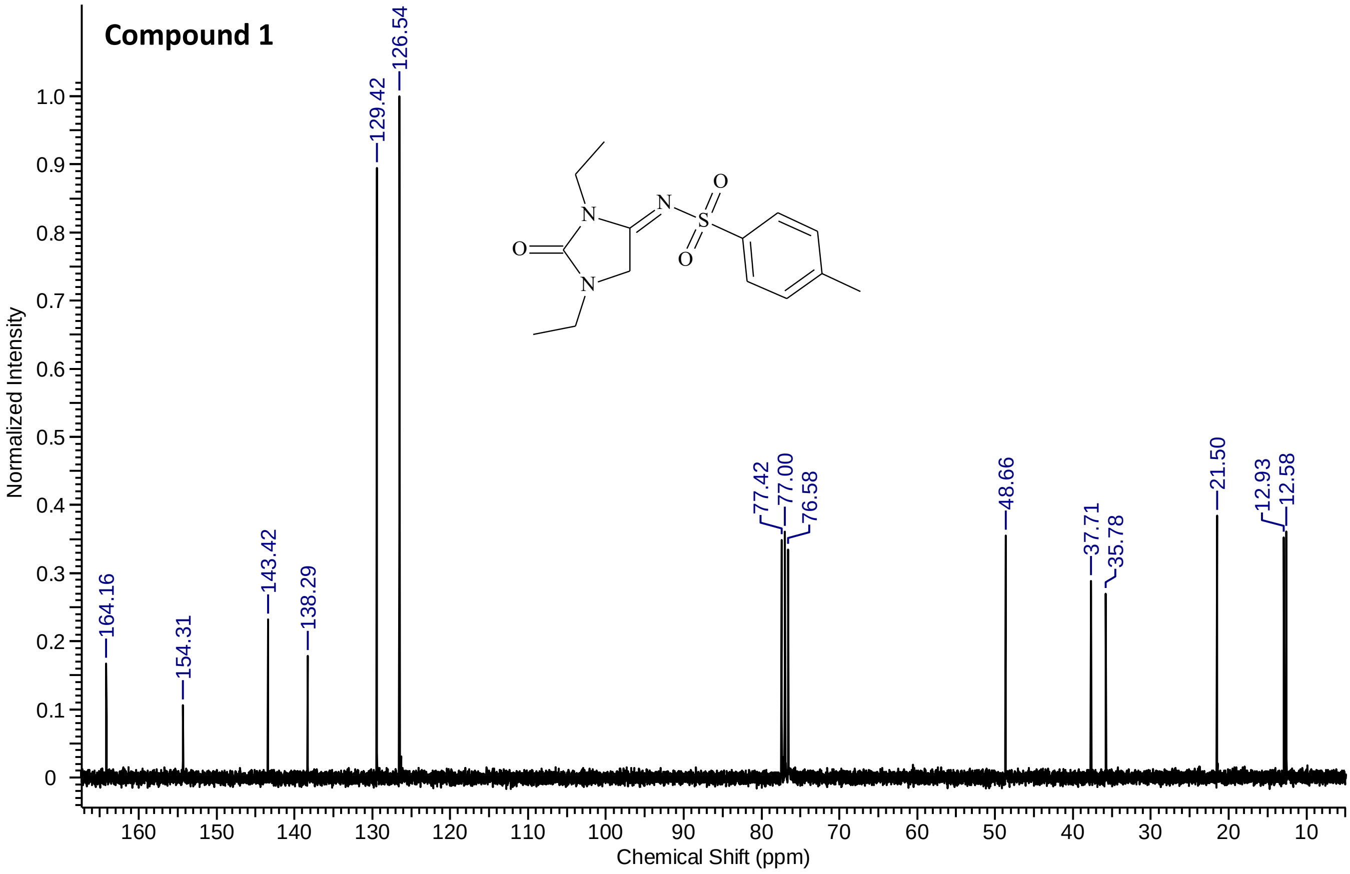
Compound 1
Normalized Intensity
Chemical Shift (ppm)
164.16 154.31 143.42 138.29 129.42 126.54 77.42 77.00 76.58 48.66 37.71 35.78 21.50 12.93 12.58
N N O N S O O

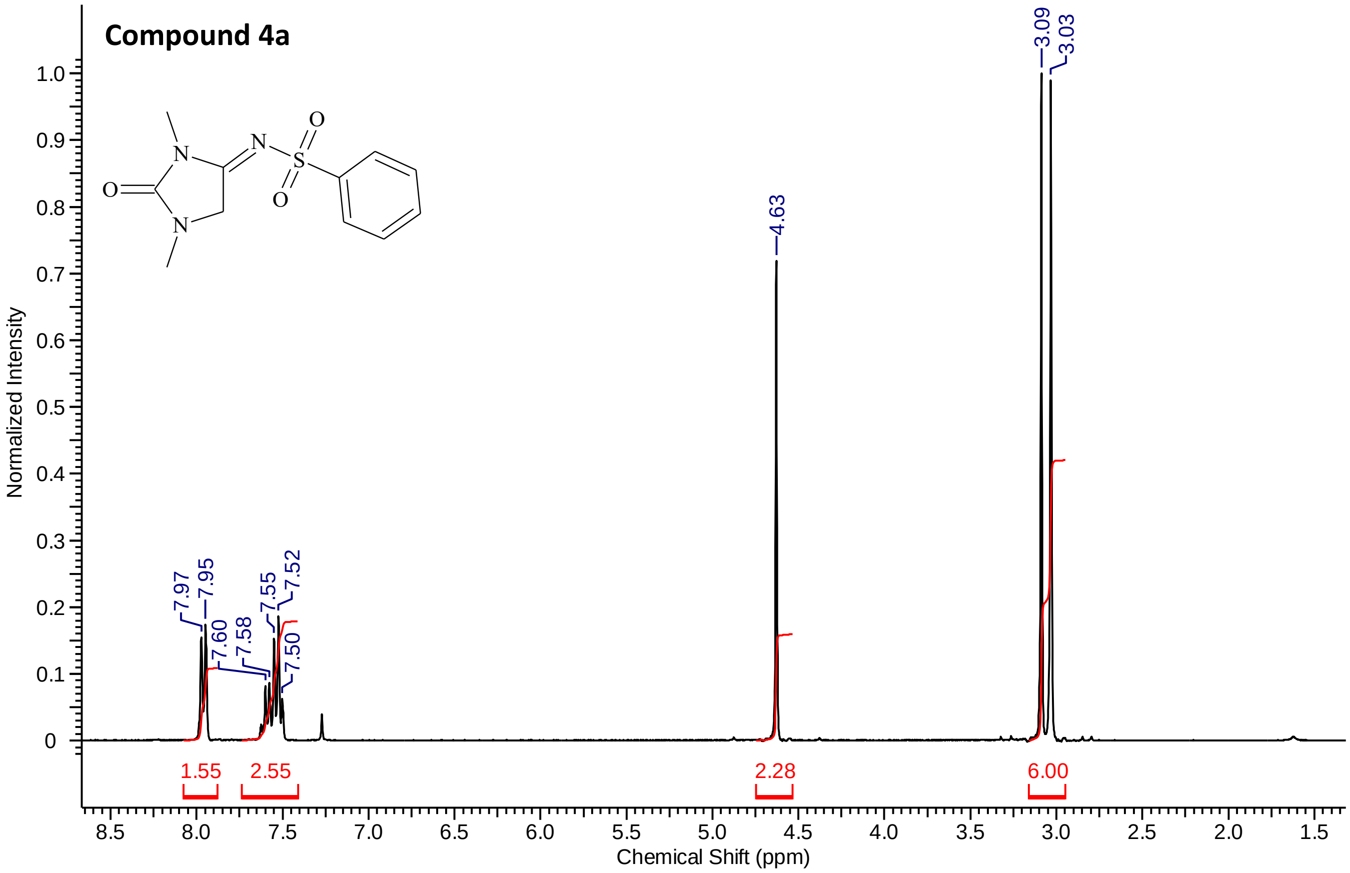

Compound 4a
Normalized Intensity
Chemical Shift (ppm)
7.97
7.95
7.60
7.58
7.55
7.52
7.50
4.63
3.09
3.03
1.55
2.55
2.28
6.00

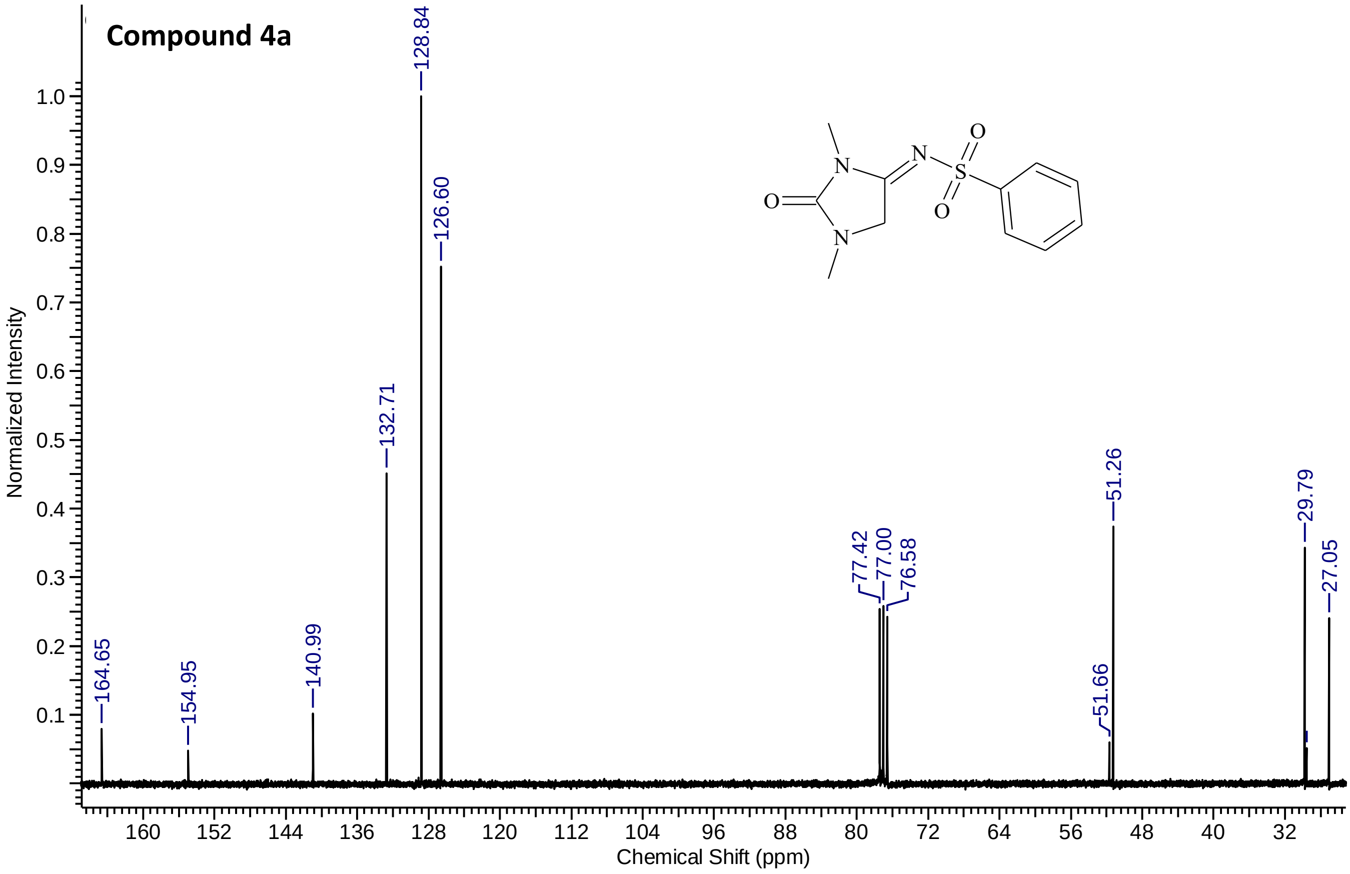

Compound 4a
Normalized Intensity
Chemical Shift (ppm)
164.65
154.95
140.99
132.71
128.84
126.60
77.42
77.00
76.58
51.66
51.26
29.79
27.05

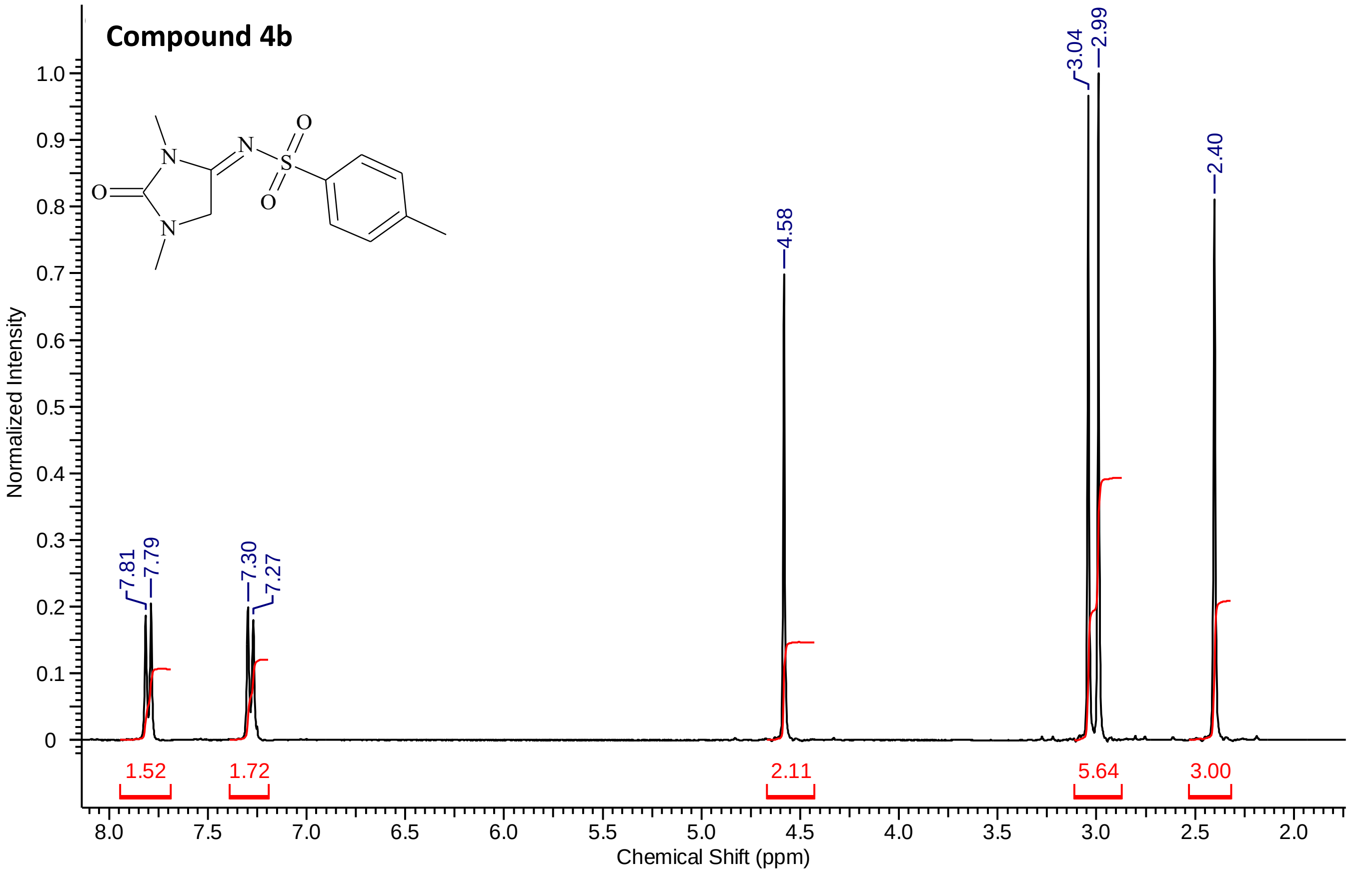
Compound 4b
7.81
7.79
7.30
7.27
4.58
3.04
2.99
2.40
1.52
1.72
2.11
5.64
3.00
Normalized Intensity
Chemical Shift (ppm)

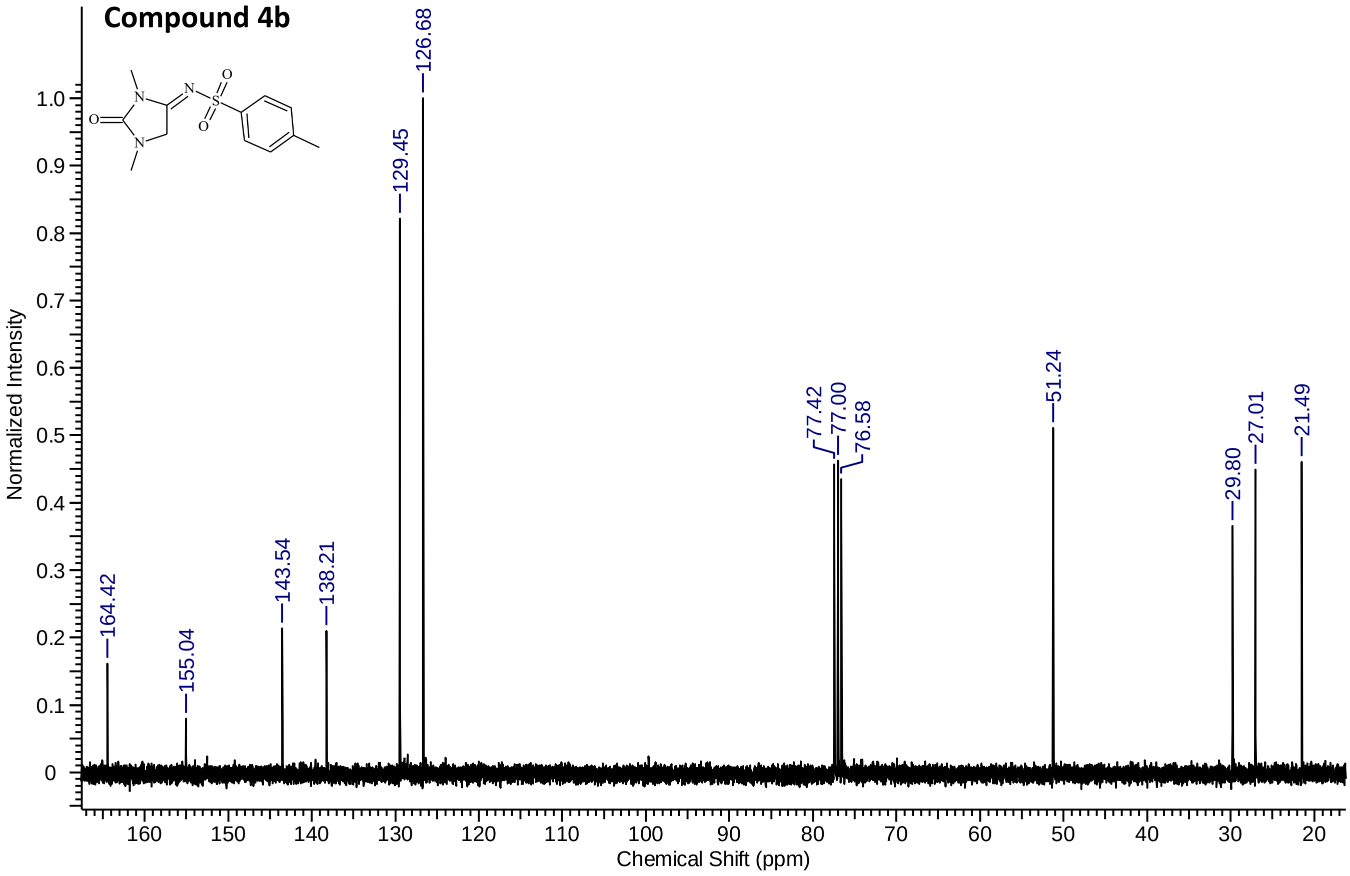
Compound 4b
164.42
155.04
143.54
138.21
129.45
126.68
77.42
77.00
76.58
51.24
29.80
27.01
21.49
Normalized Intensity
Chemical Shift (ppm)

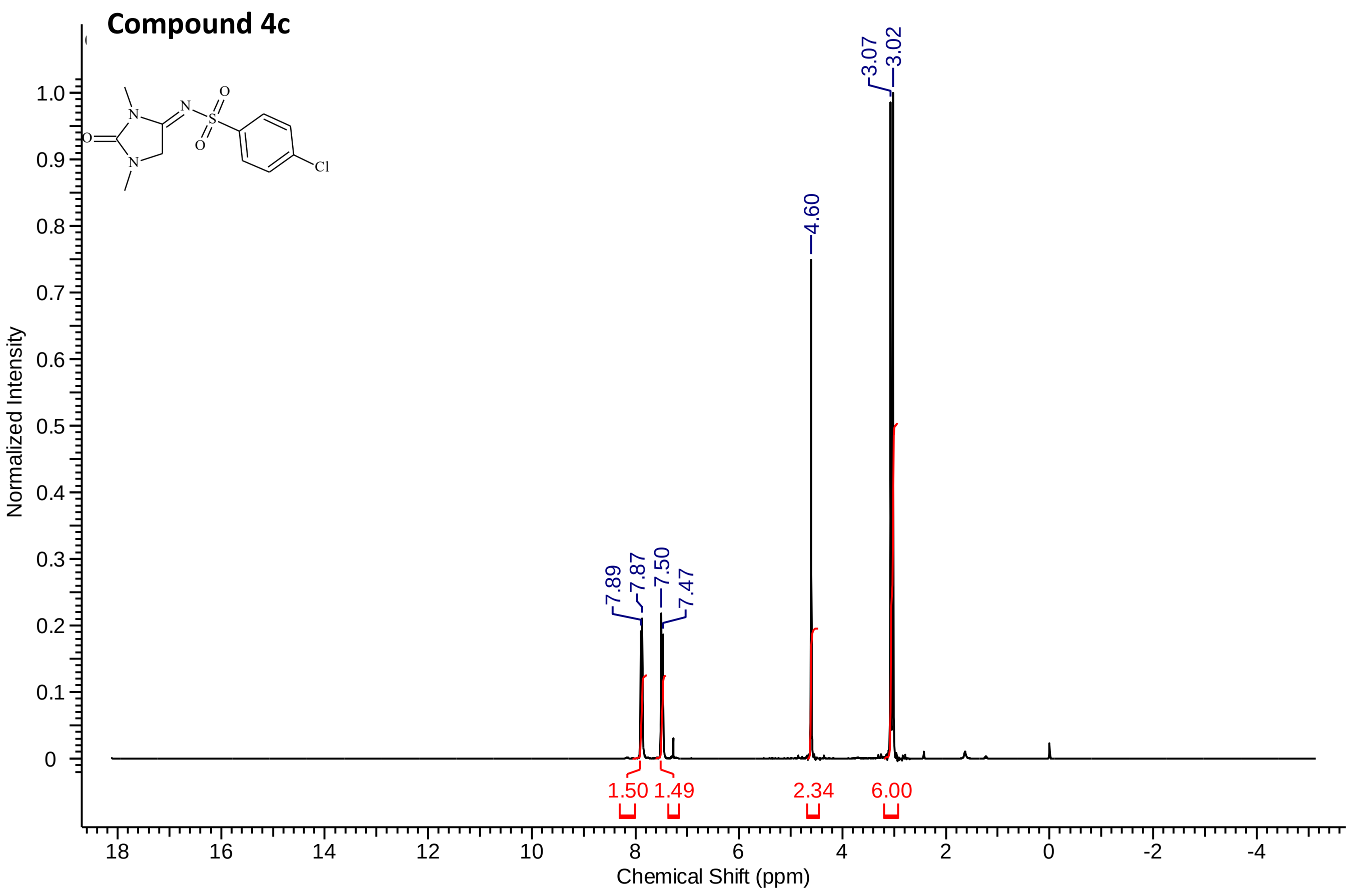
Compound 4c
N
N
O
N
S
O
O
Cl
Normalized Intensity
Chemical Shift (ppm)
7.89
7.87
7.50
7.47
4.60
3.07
3.02
1.50
1.49
2.34
6.00

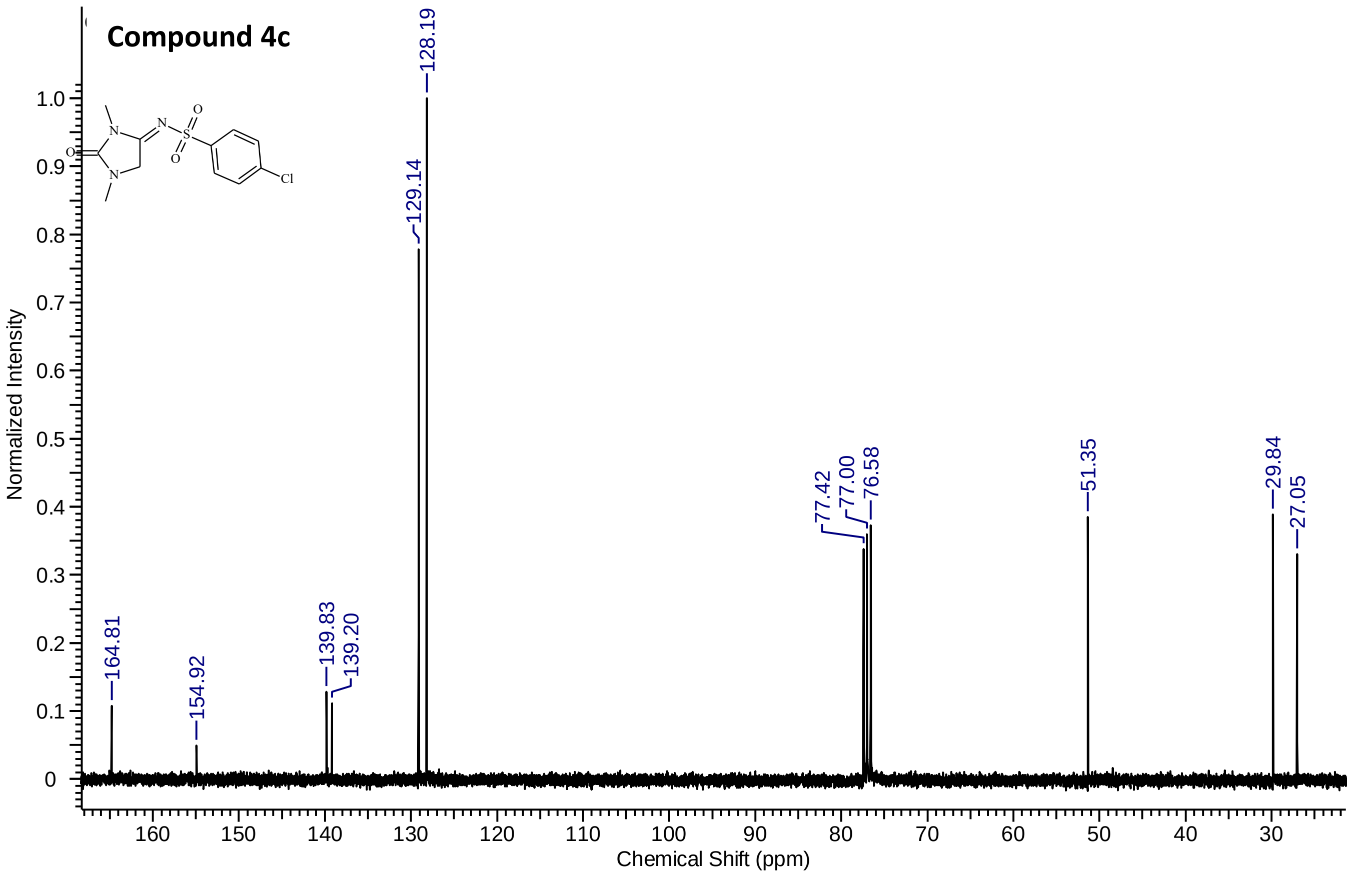
Compound 4c
N
N
O
N
S
O
O
Cl
Normalized Intensity
Chemical Shift (ppm)
164.81
154.92
139.83
139.20
129.14
128.19
77.42
77.00
76.58
51.35
29.84
27.05

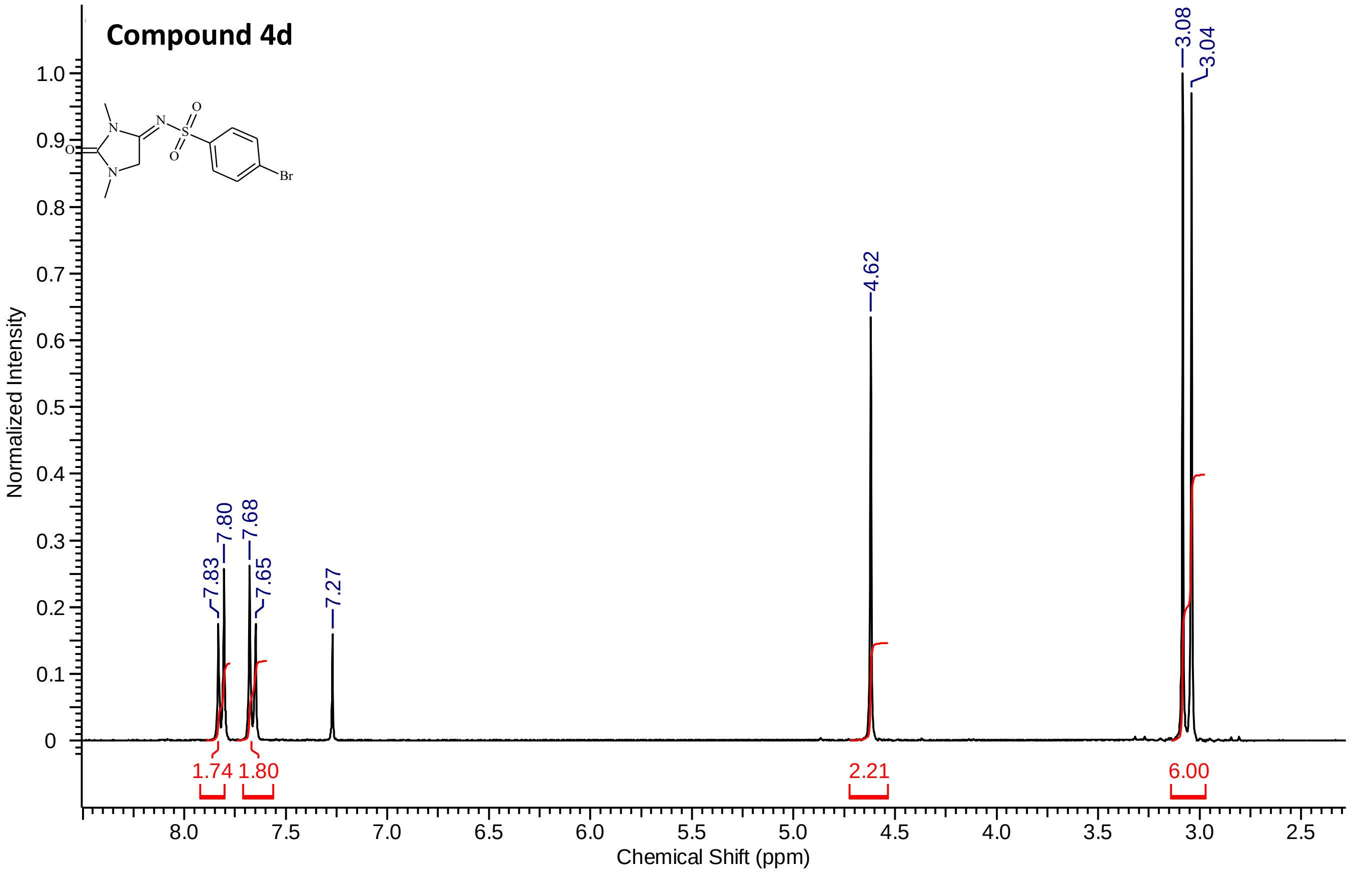
Compound 4d
Br
Normalized Intensity
Chemical Shift (ppm)
7.83
7.80
7.68
7.65
7.27
4.62
3.08
3.04
1.74
1.80
2.21
6.00

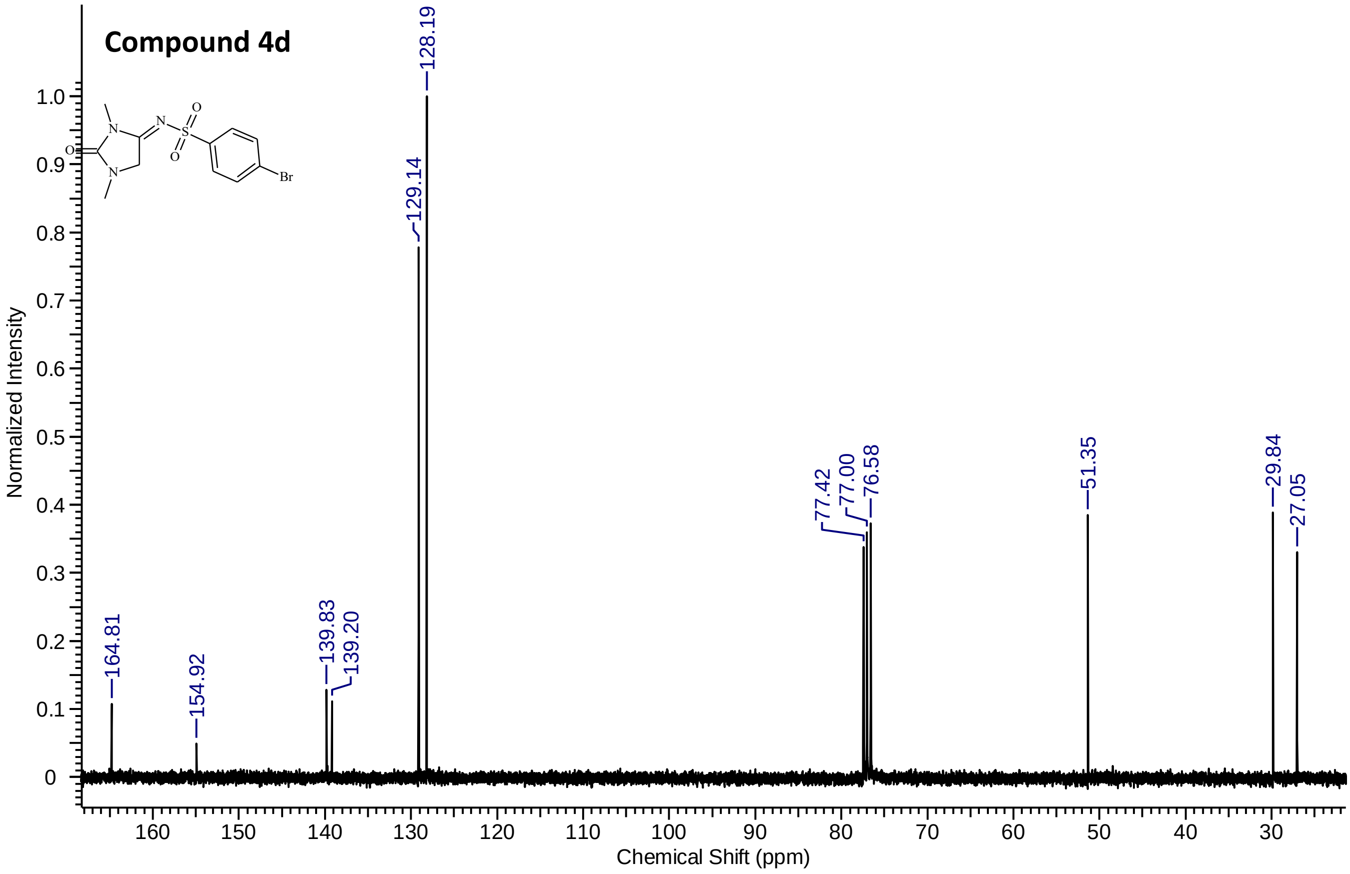
Compound 4d
Br
Normalized Intensity
Chemical Shift (ppm)
164.81
154.92
139.83
139.20
129.14
128.19
77.42
77.00
76.58
51.35
29.84
27.05

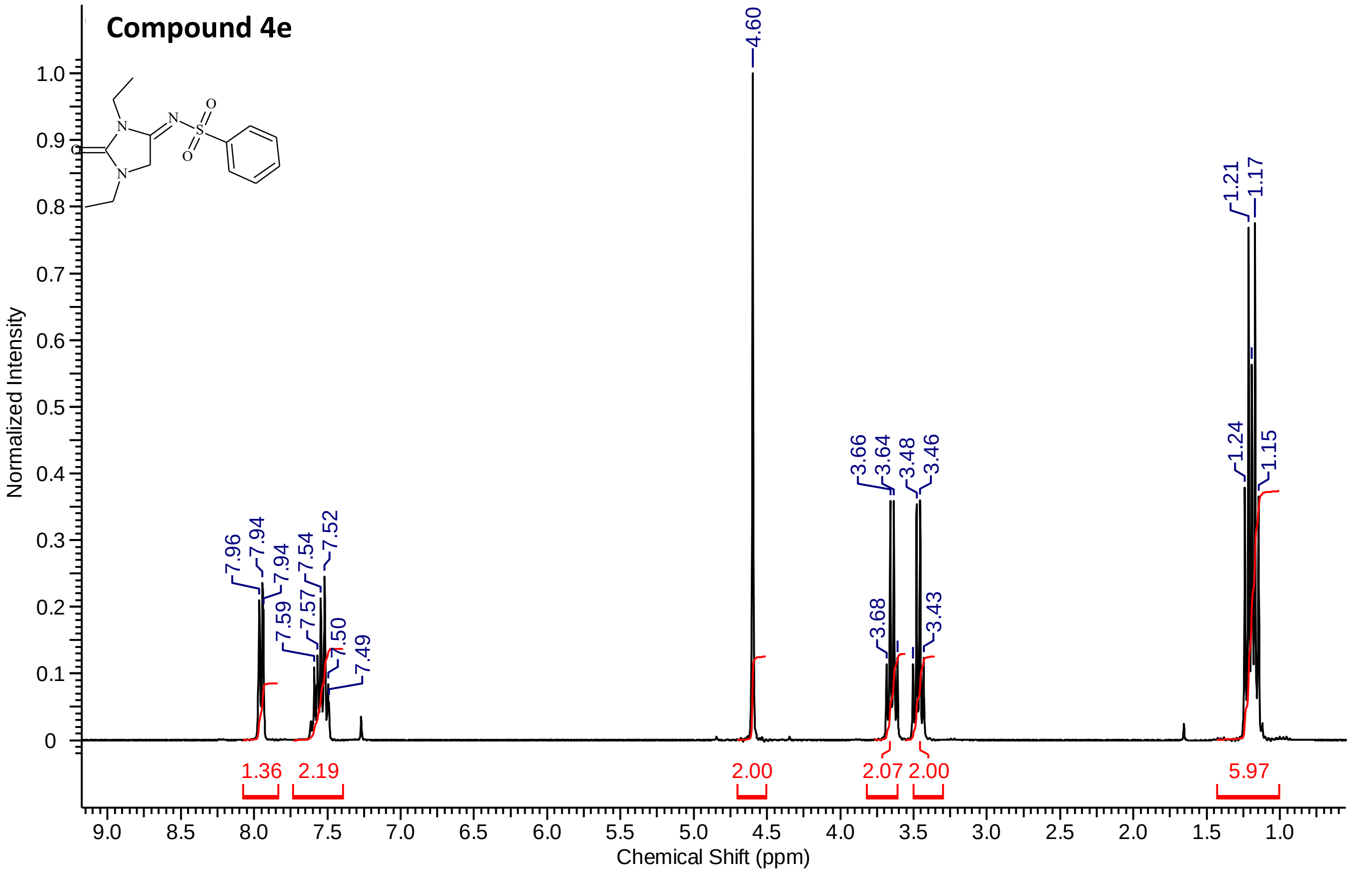
Compound 4e
Normalized Intensity
Chemical Shift (ppm)
4.60
3.66 3.64 3.48 3.46 3.68 3.43
1.21 1.17 1.24 1.15
7.96 7.94 7.94 7.59 7.57 7.54 7.52 7.50 7.49
1.36 2.19 2.00 2.07 2.00 5.97

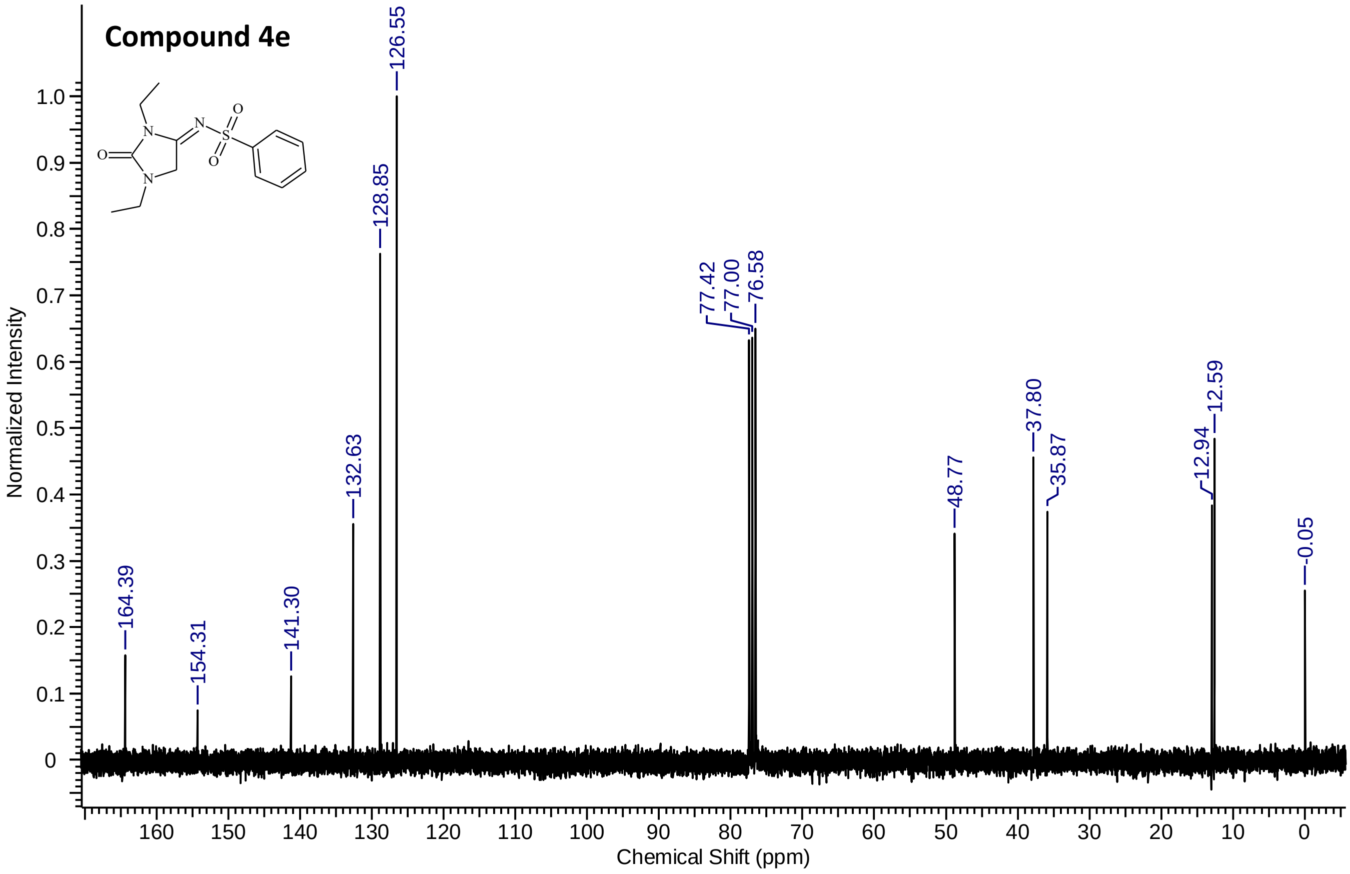
Compound 4e
Normalized Intensity
Chemical Shift (ppm)
164.39 154.31 141.30 132.63 128.85 126.55
77.42 77.00 76.58
48.77 37.80 35.87 12.94 12.59 -0.05

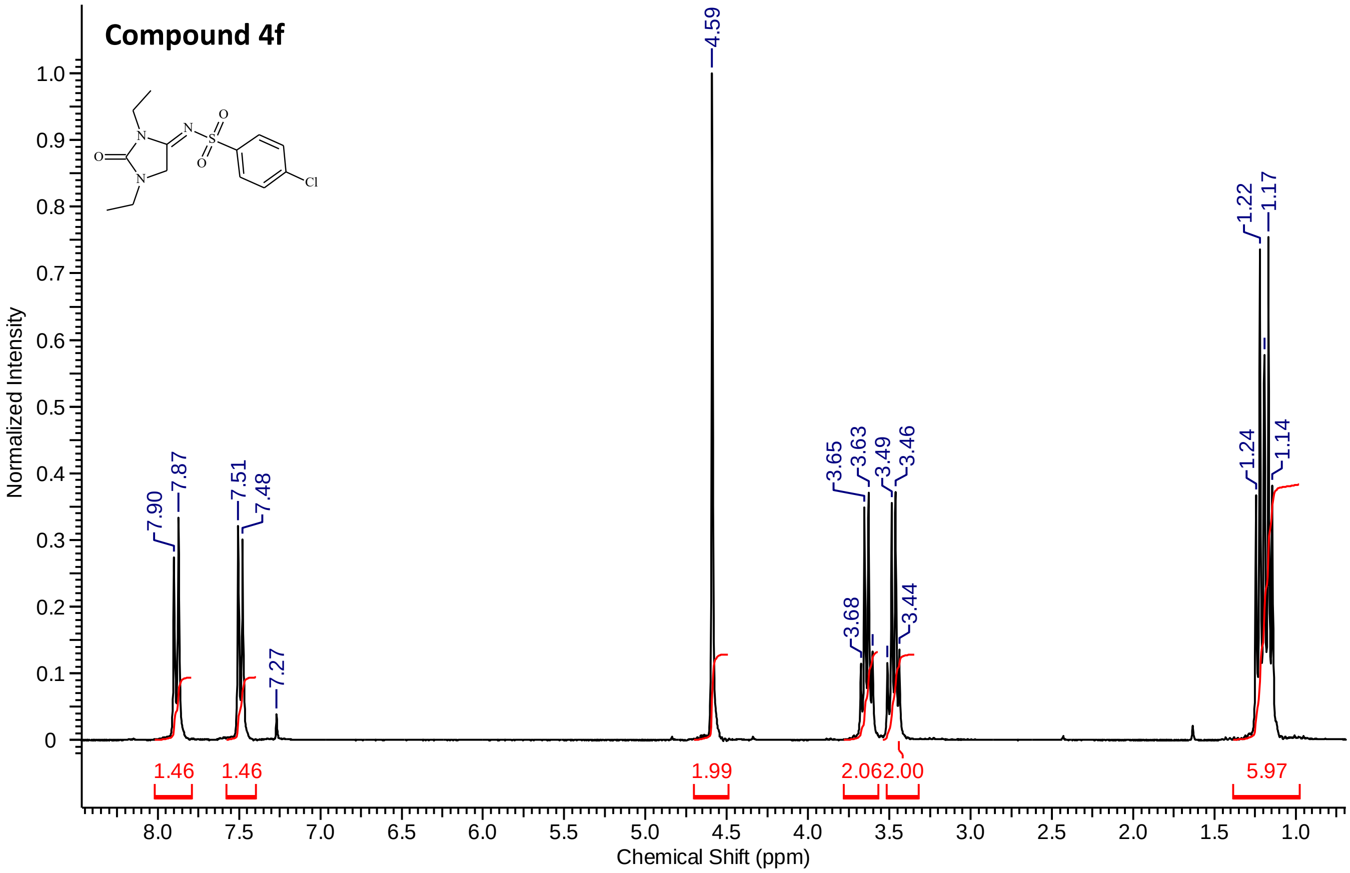

Compound 4f
Normalized Intensity
Chemical Shift (ppm)
7.90
7.87
7.51
7.48
7.27
4.59
3.68
3.65
3.63
3.49
3.46
3.44
1.24
1.22
1.17
1.14
1.46
1.46
1.99
2.06
2.00
5.97

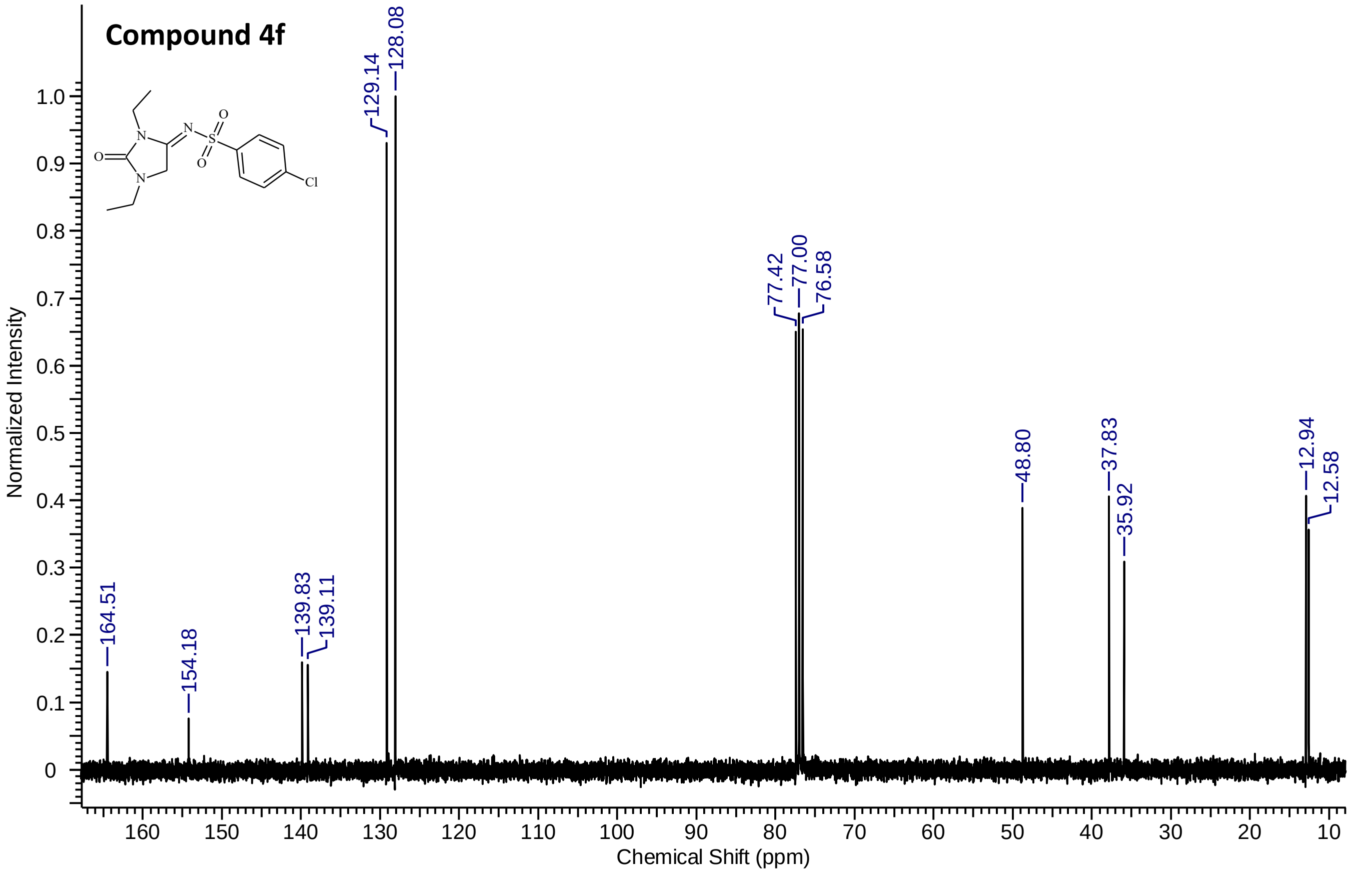

Compound 4f
Normalized Intensity
Chemical Shift (ppm)
164.51
154.18
139.83
139.11
129.14
128.08
77.42
77.00
76.58
48.80
37.83
35.92
12.94
12.58

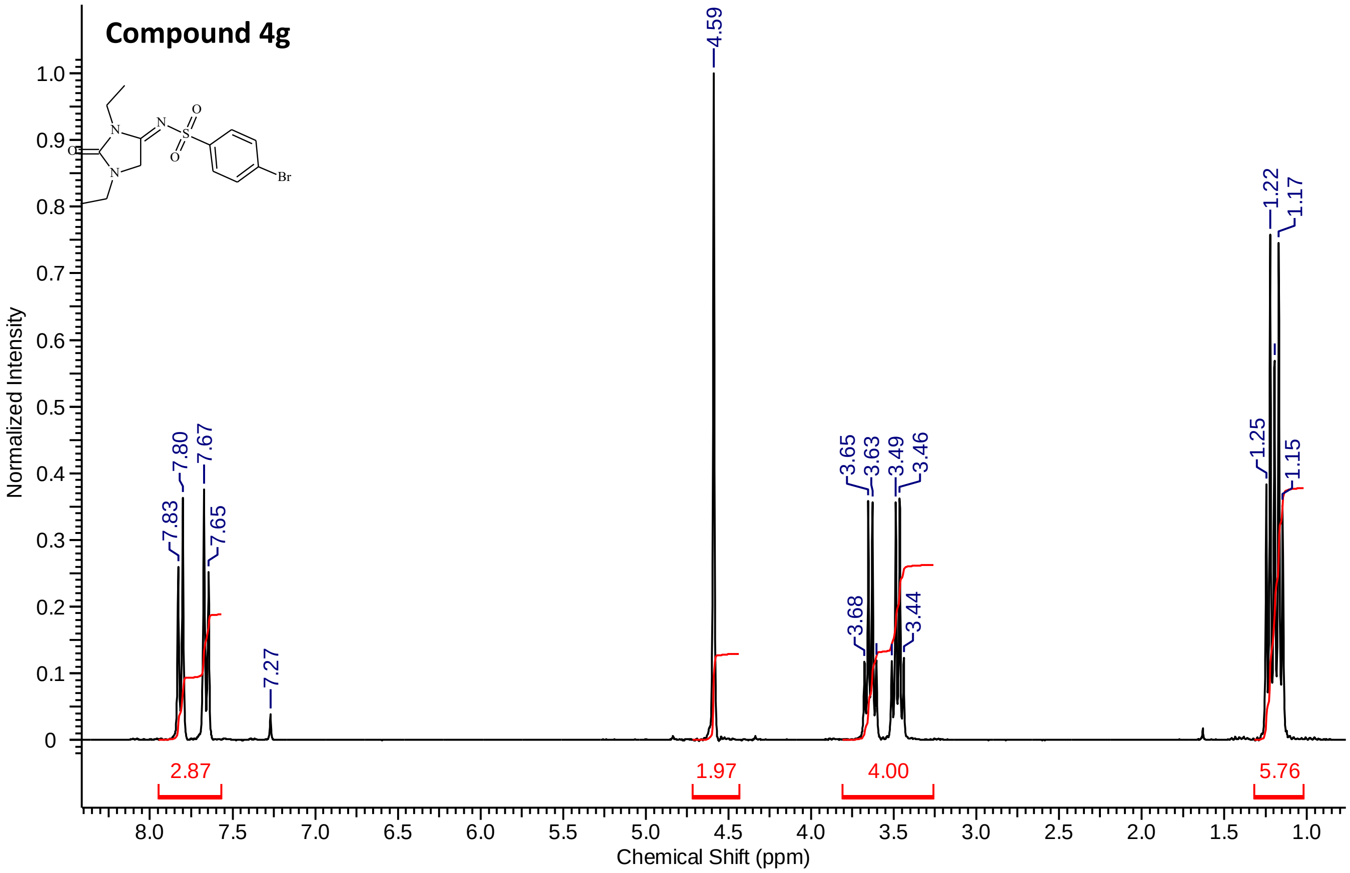
Compound 4g
Normalized Intensity
Chemical Shift (ppm)
7.83
7.80
7.67
7.65
7.27
4.59
3.65
3.63
3.49
3.46
3.68
3.44
1.22
1.17
1.25
1.15
2.87
1.97
4.00
5.76

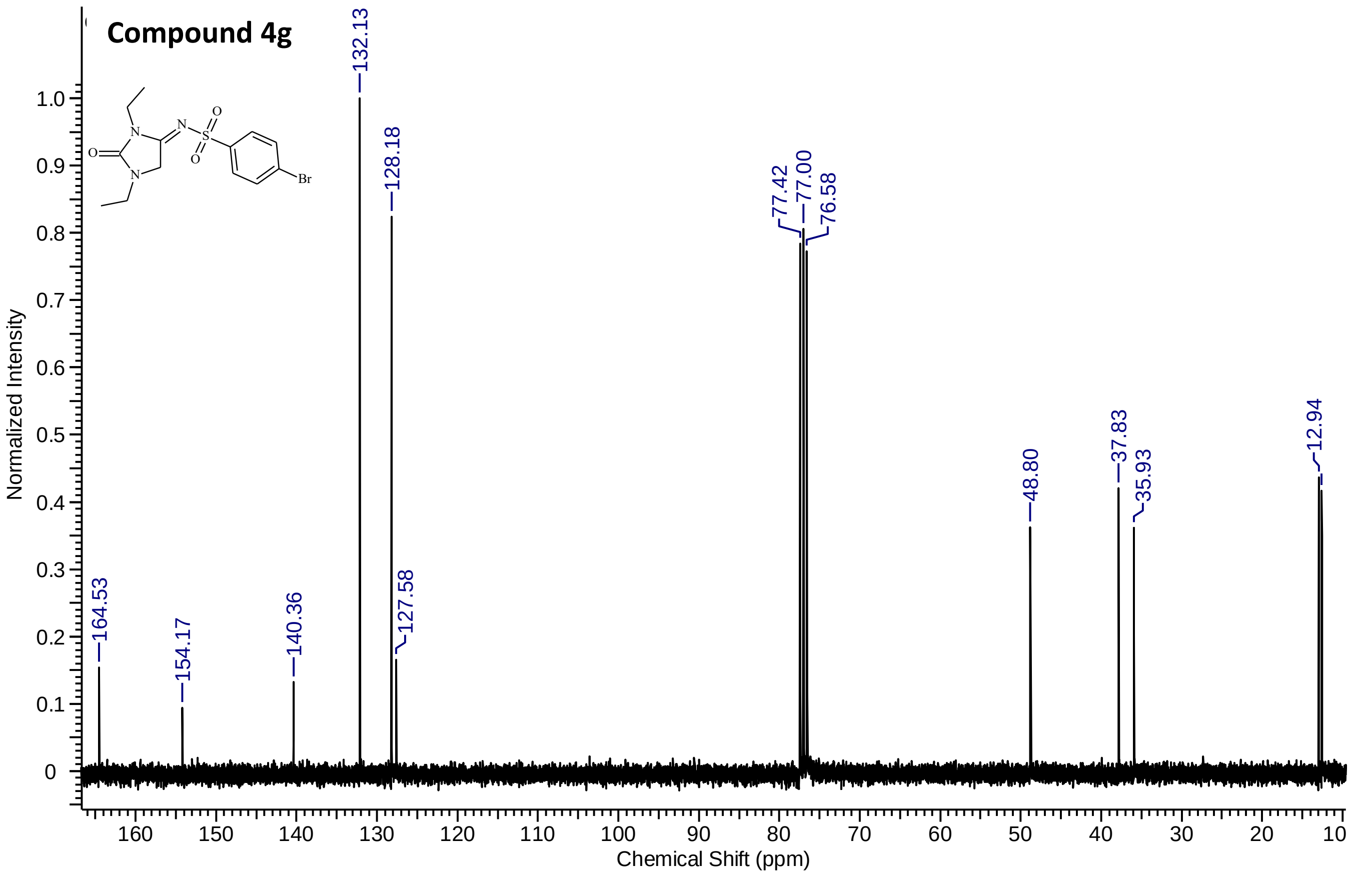
Compound 4g
Normalized Intensity
Chemical Shift (ppm)
164.53
154.17
140.36
132.13
128.18
127.58
77.42
77.00
76.58
48.80
37.83
35.93
12.94

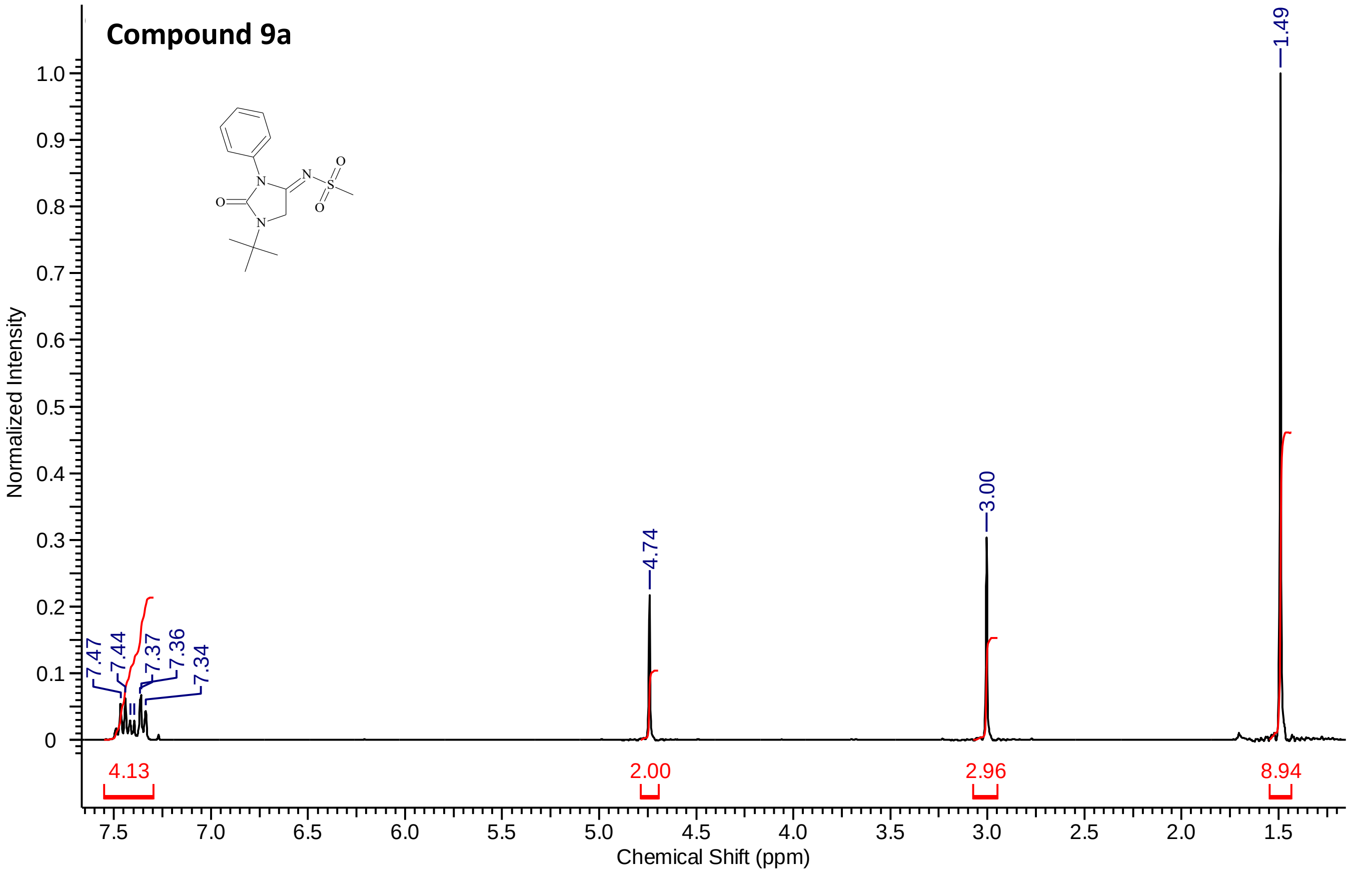
Compound 9a
Normalized Intensity
Chemical Shift (ppm)
7.47
7.44
7.37
7.36
7.34
4.74
3.00
1.49
4.13
2.00
2.96
8.94

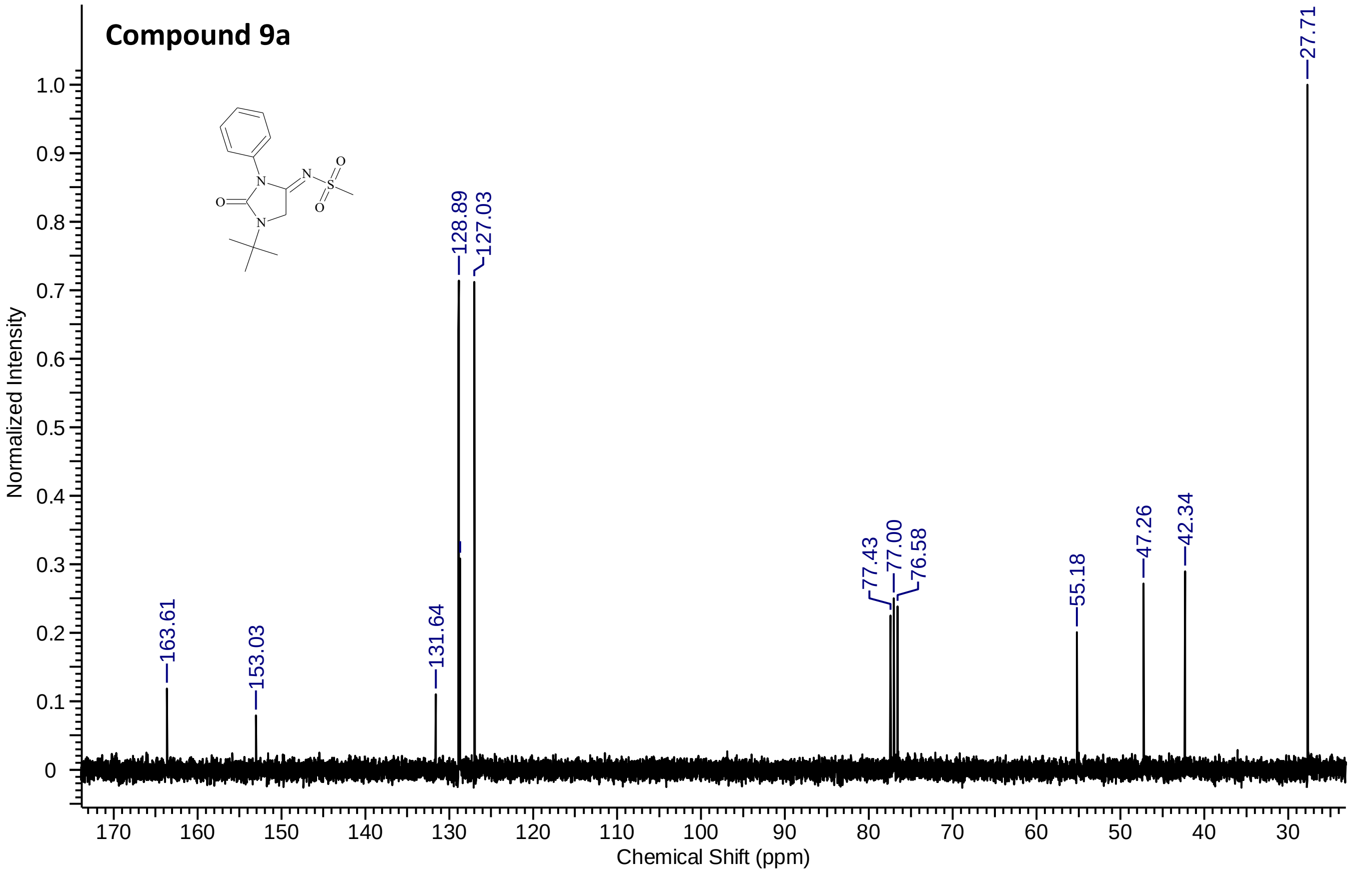
Compound 9a
Normalized Intensity
Chemical Shift (ppm)
163.61
153.03
131.64
128.89
127.03
77.43
77.00
76.58
55.18
47.26
42.34
27.71

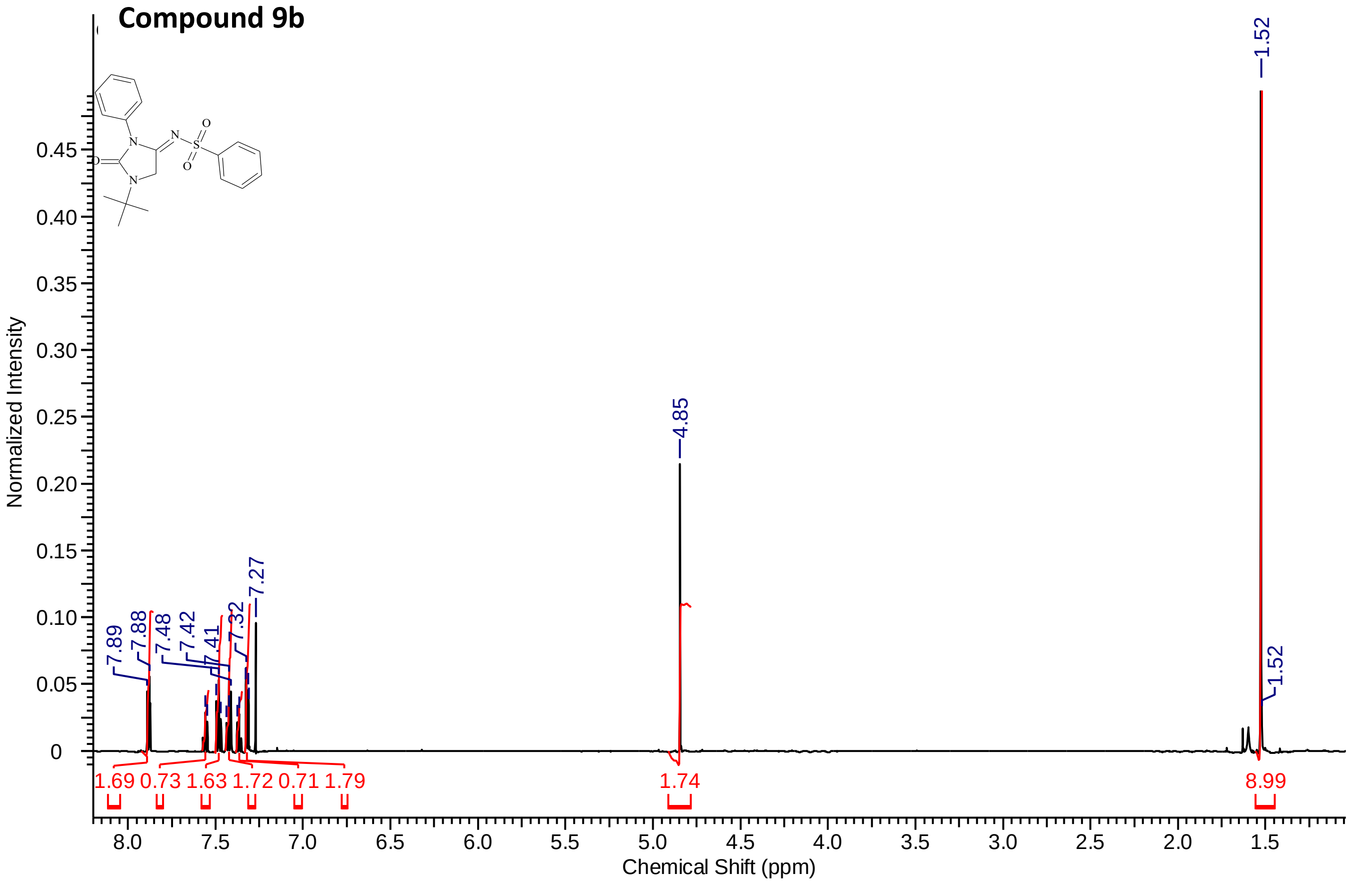
Compound 9b
N
O
S
N
N
7.89
7.88
7.48
7.42
7.41
7.32
7.27
4.85
1.52
1.52
1.69
0.73
1.63
1.72
0.71
1.79
1.74
8.99
Normalized Intensity
Chemical Shift (ppm)

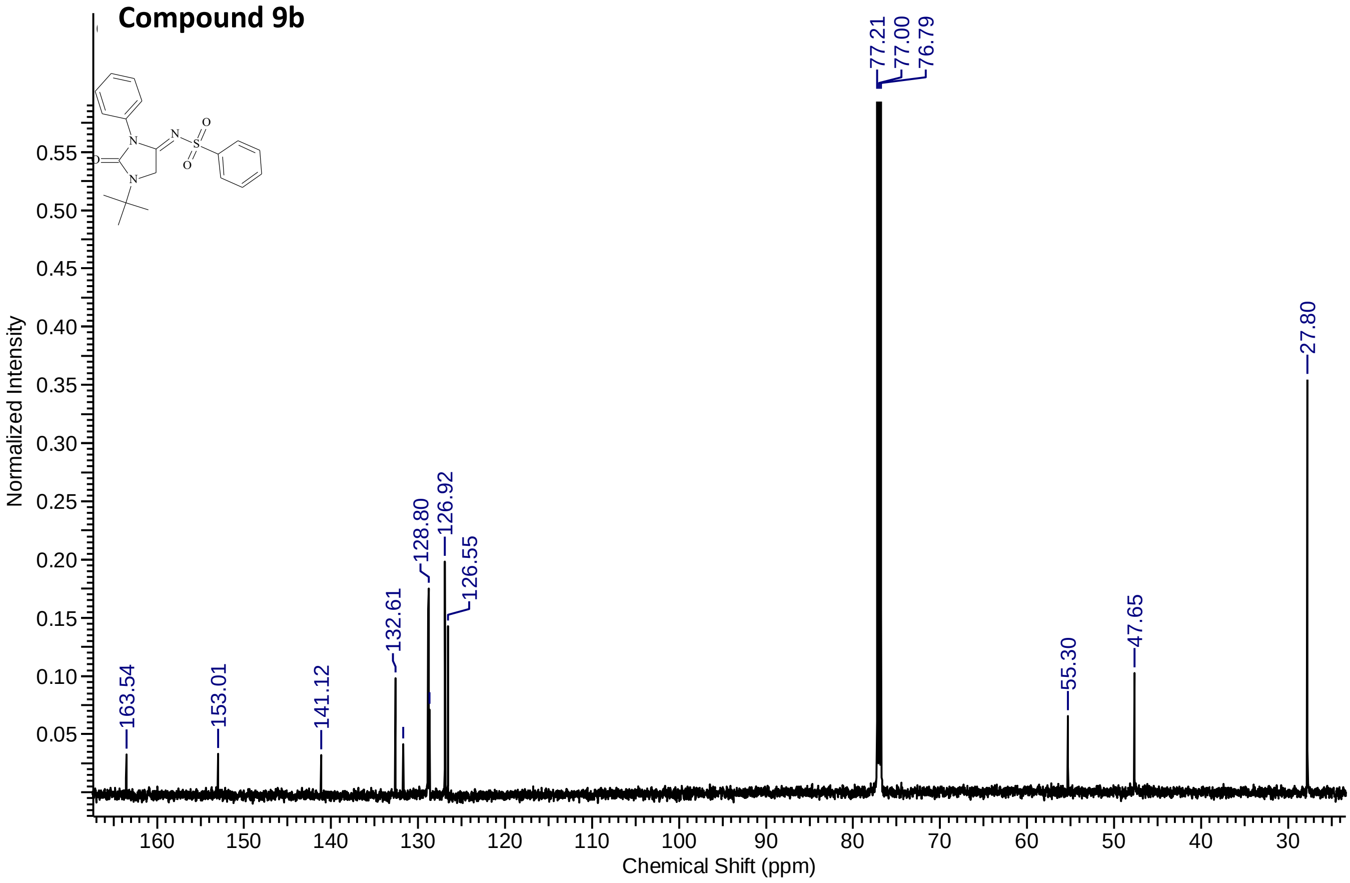
Compound 9b
N
O
S
N
N
163.54
153.01
141.12
132.61
128.80
126.92
126.55
77.21
77.00
76.79
55.30
47.65
27.80
Normalized Intensity
Chemical Shift (ppm)

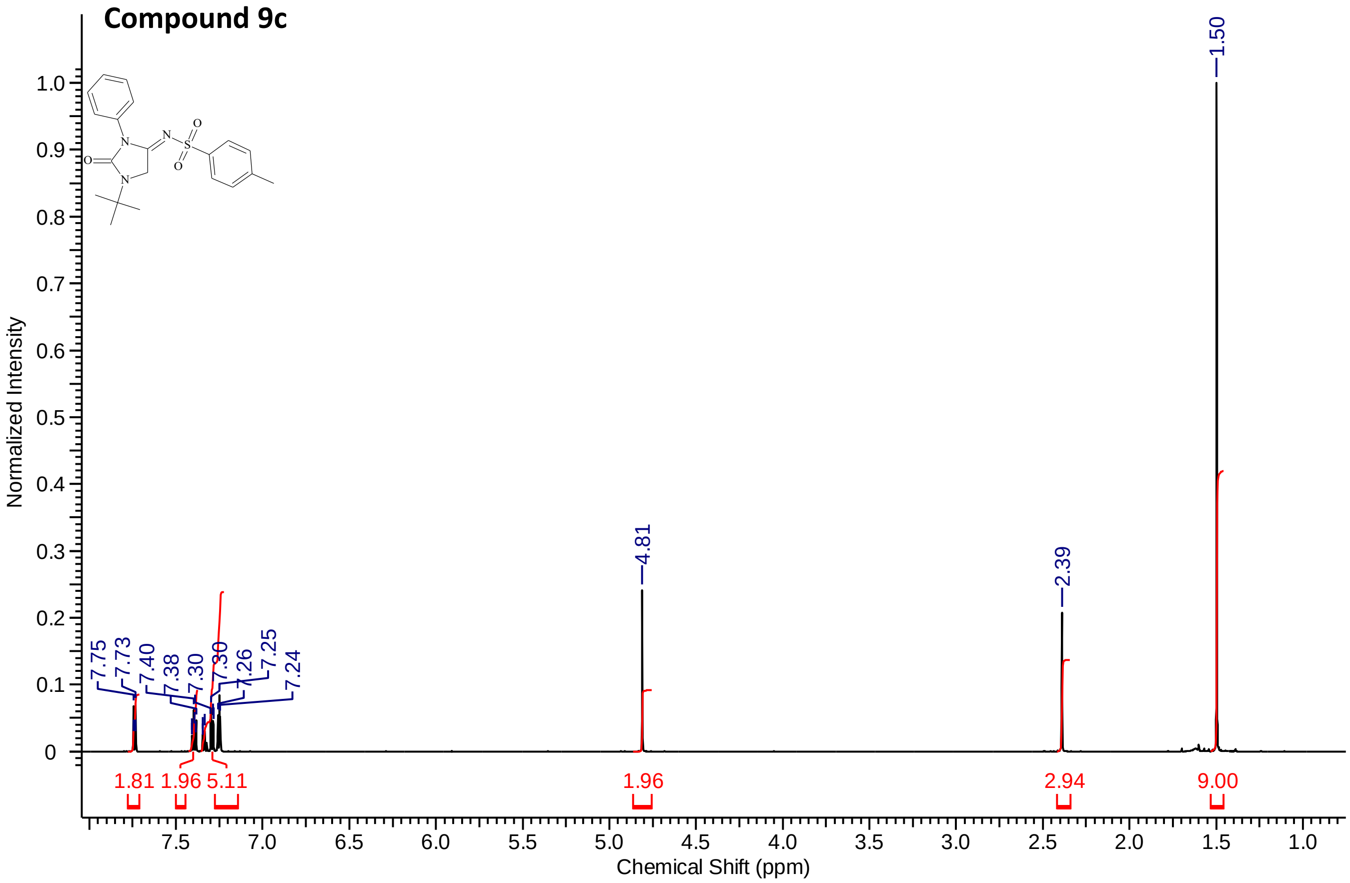
Compound 9c
Normalized Intensity
Chemical Shift (ppm)
7.75
7.73
7.40
7.38
7.30
7.30
7.26
7.25
7.24
4.81
2.39
1.50
1.81
1.96
5.11
1.96
2.94
9.00

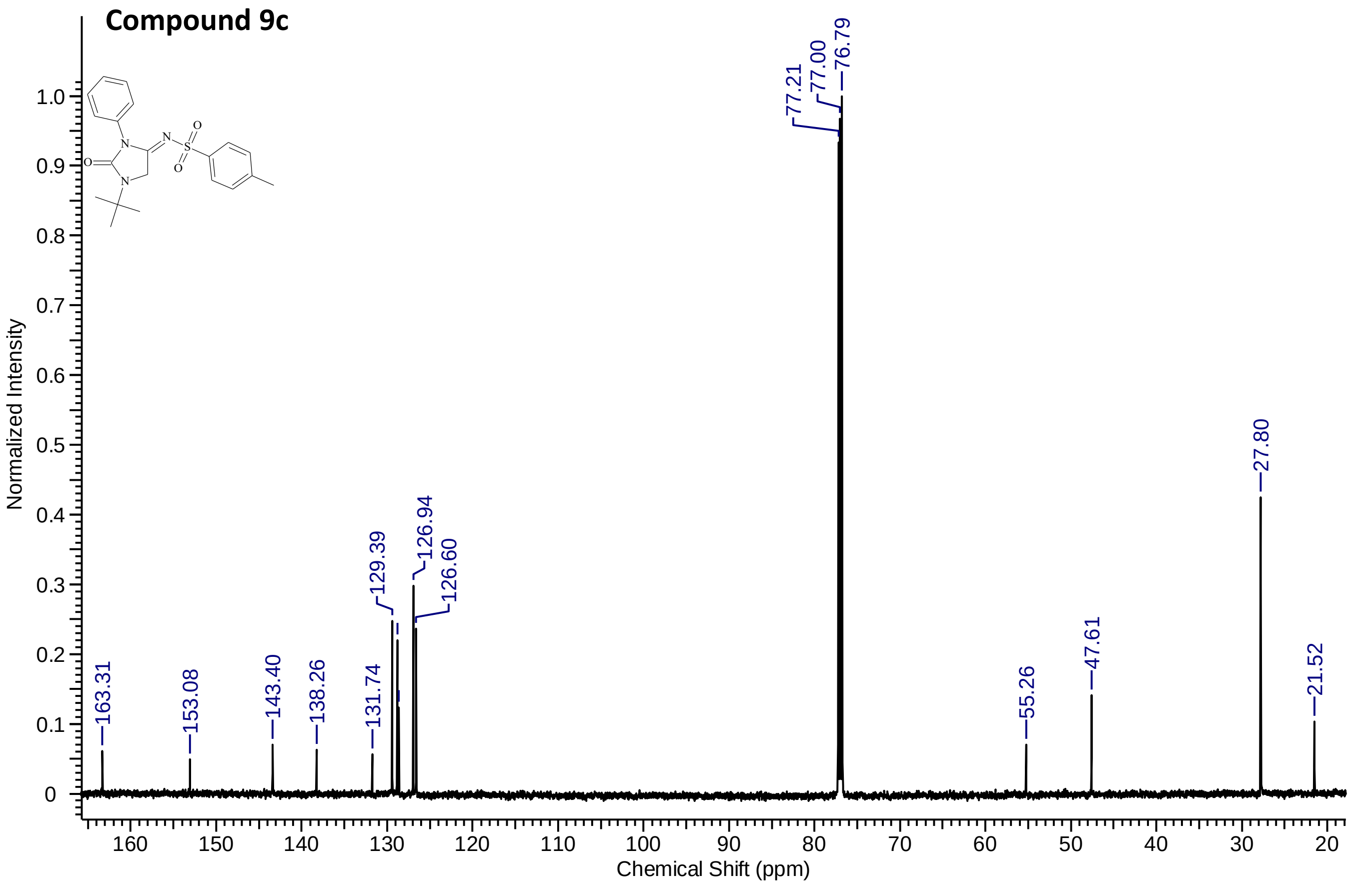
Compound 9c
Normalized Intensity
Chemical Shift (ppm)
163.31
153.08
143.40
138.26
131.74
129.39
126.94
126.60
77.21
77.00
76.79
55.26
47.61
27.80
21.52

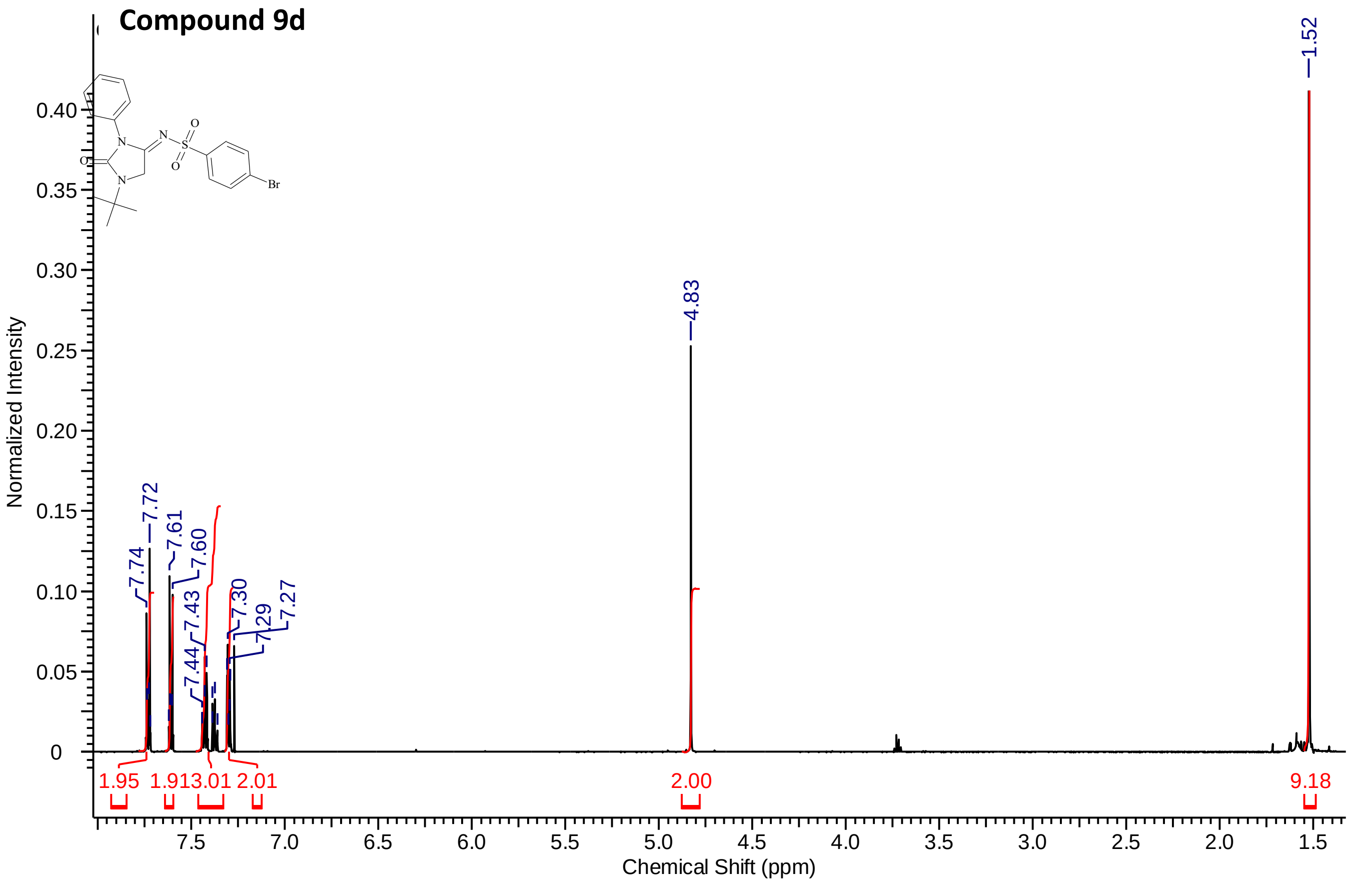
Compound 9d
Normalized Intensity
Chemical Shift (ppm)
7.74
7.72
7.61
7.60
7.43
7.44
7.30
7.29
7.27
4.83
1.52
1.95
1.91
3.01
2.01
2.00
9.18
Br

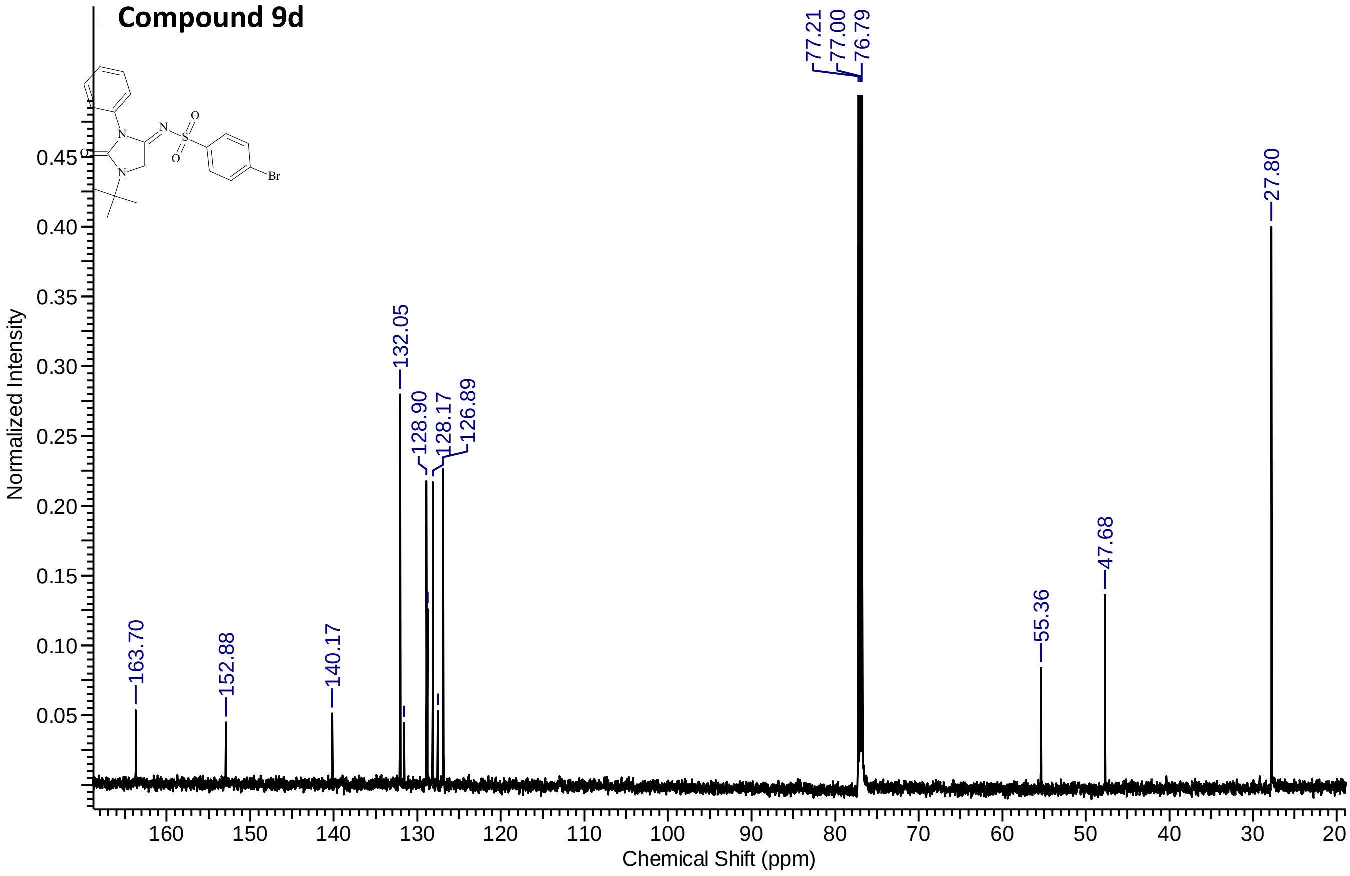
Compound 9d
Normalized Intensity
Chemical Shift (ppm)
163.70
152.88
140.17
132.05
128.90
128.17
126.89
77.21
77.00
76.79
55.36
47.68
27.80
Br

Compound 9e

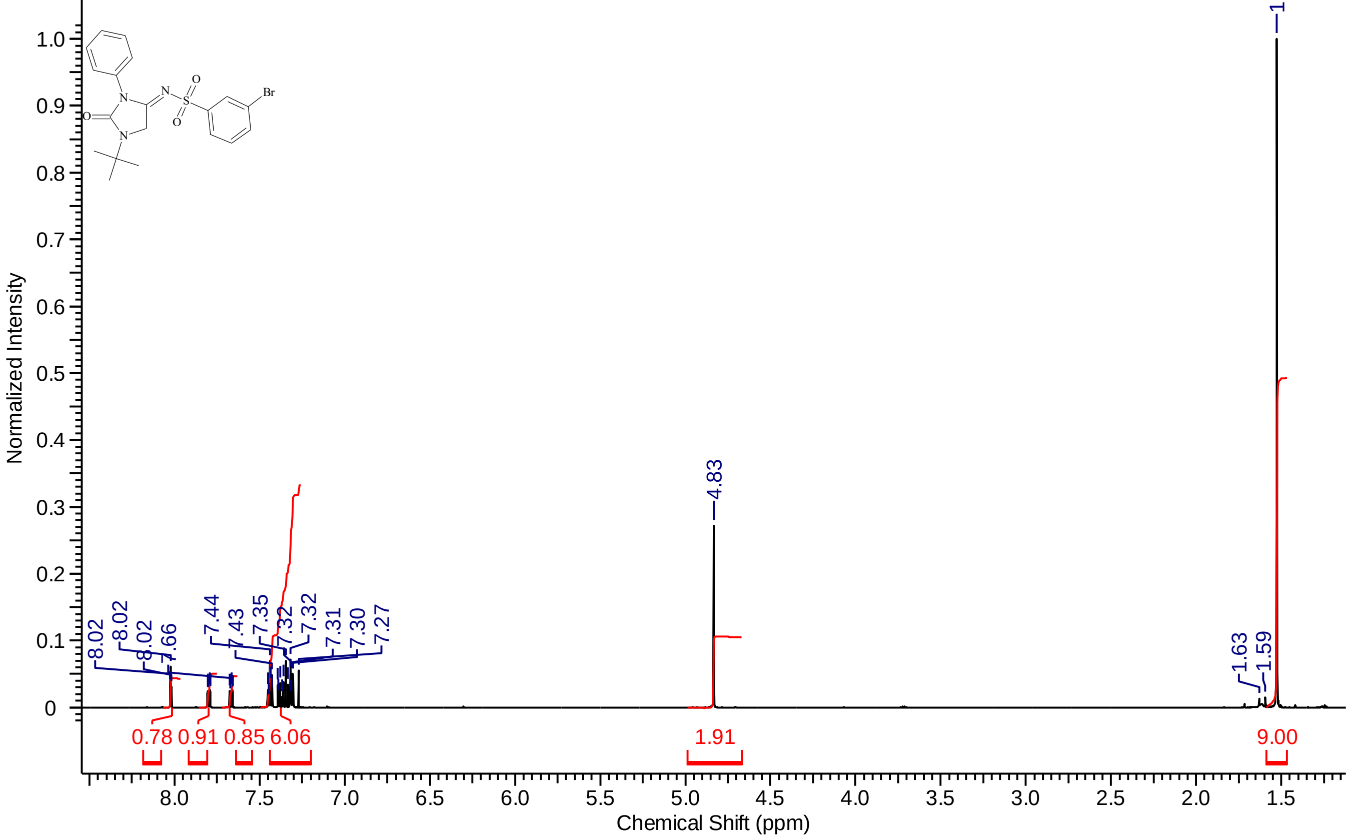

Compound 9e

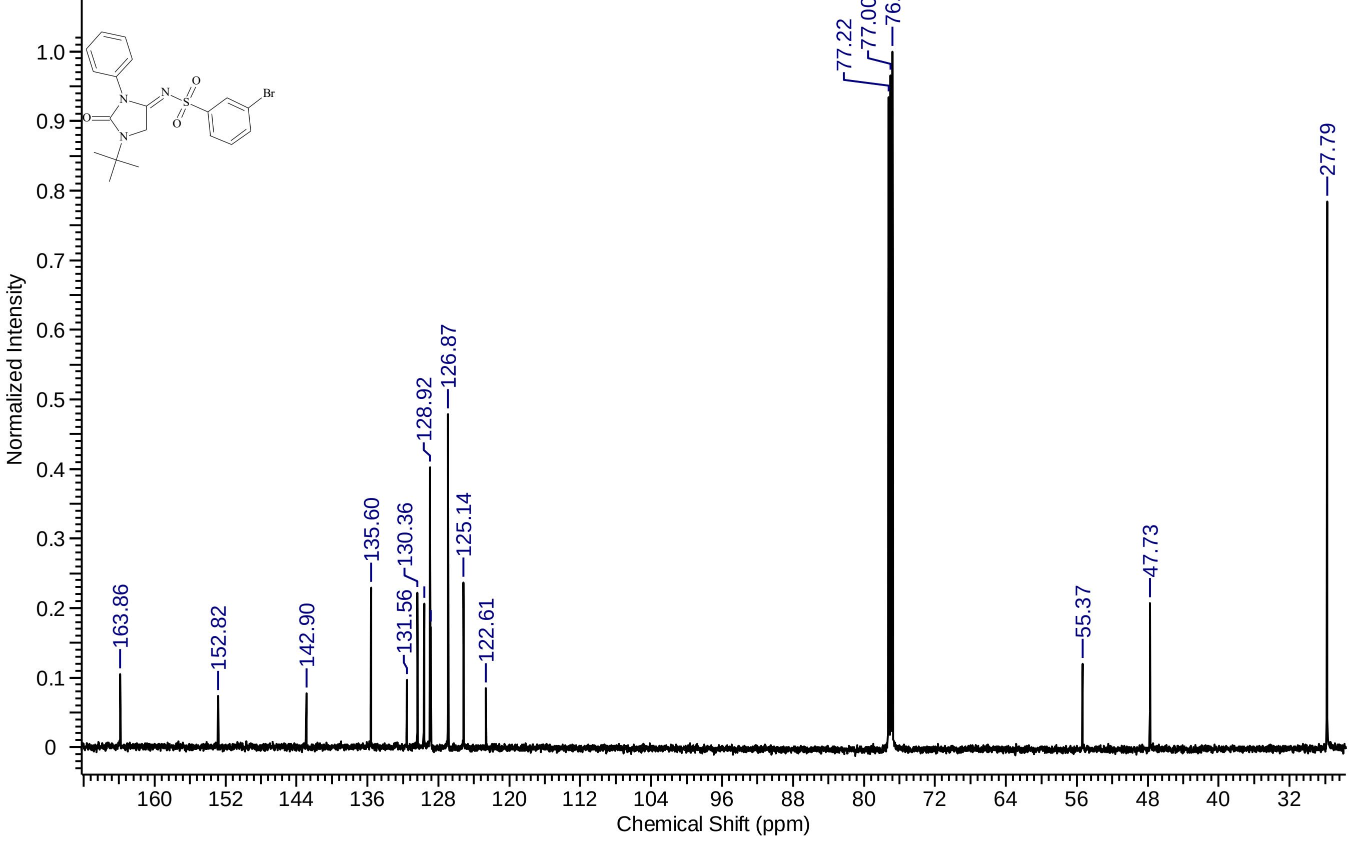

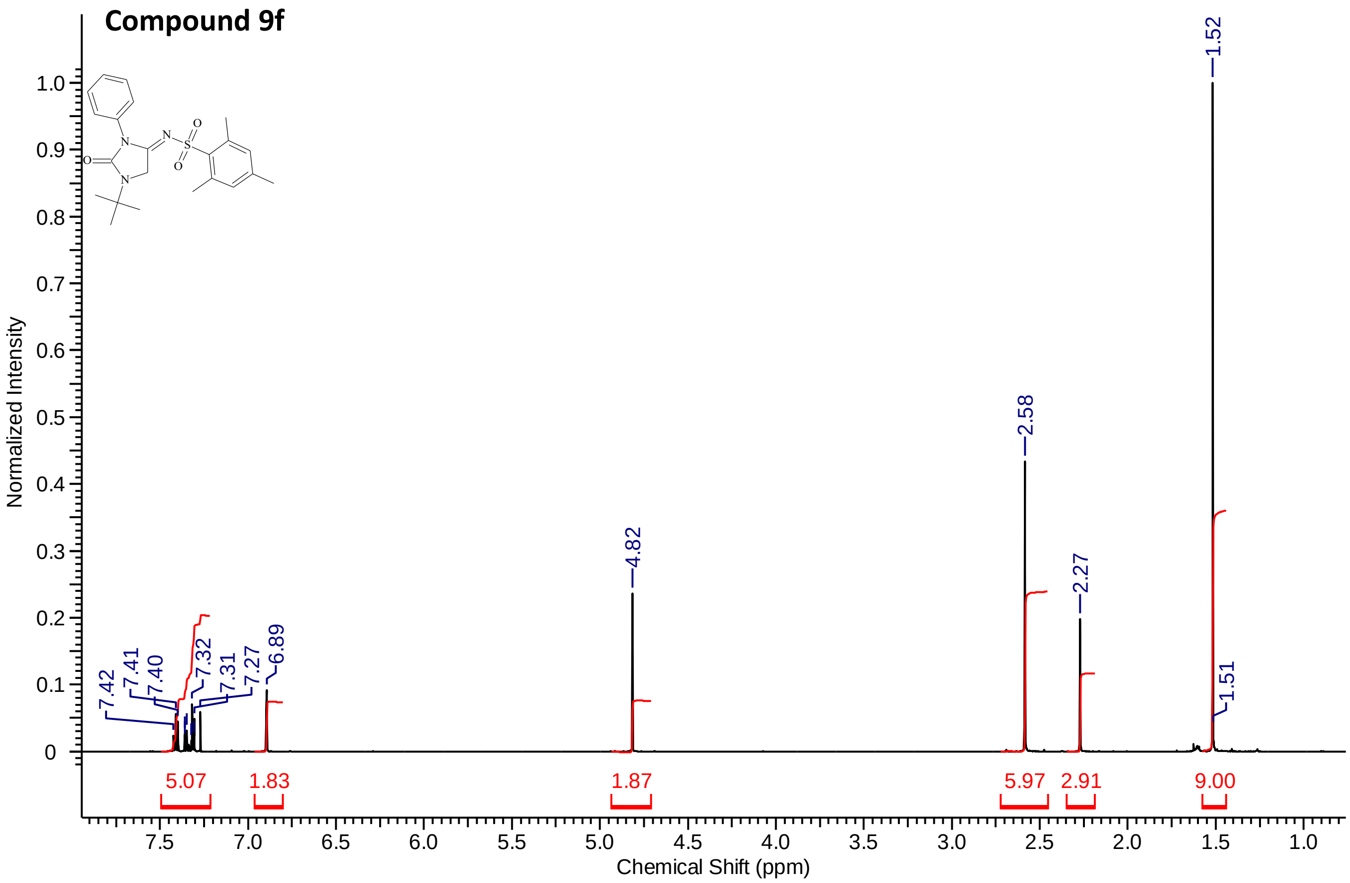
Compound 9f
Normalized Intensity
Chemical Shift (ppm)
7.42
7.41
7.40
7.32
7.31
7.27
6.89
4.82
2.58
2.27
1.52
1.51
5.07
1.83
1.87
5.97
2.91
9.00

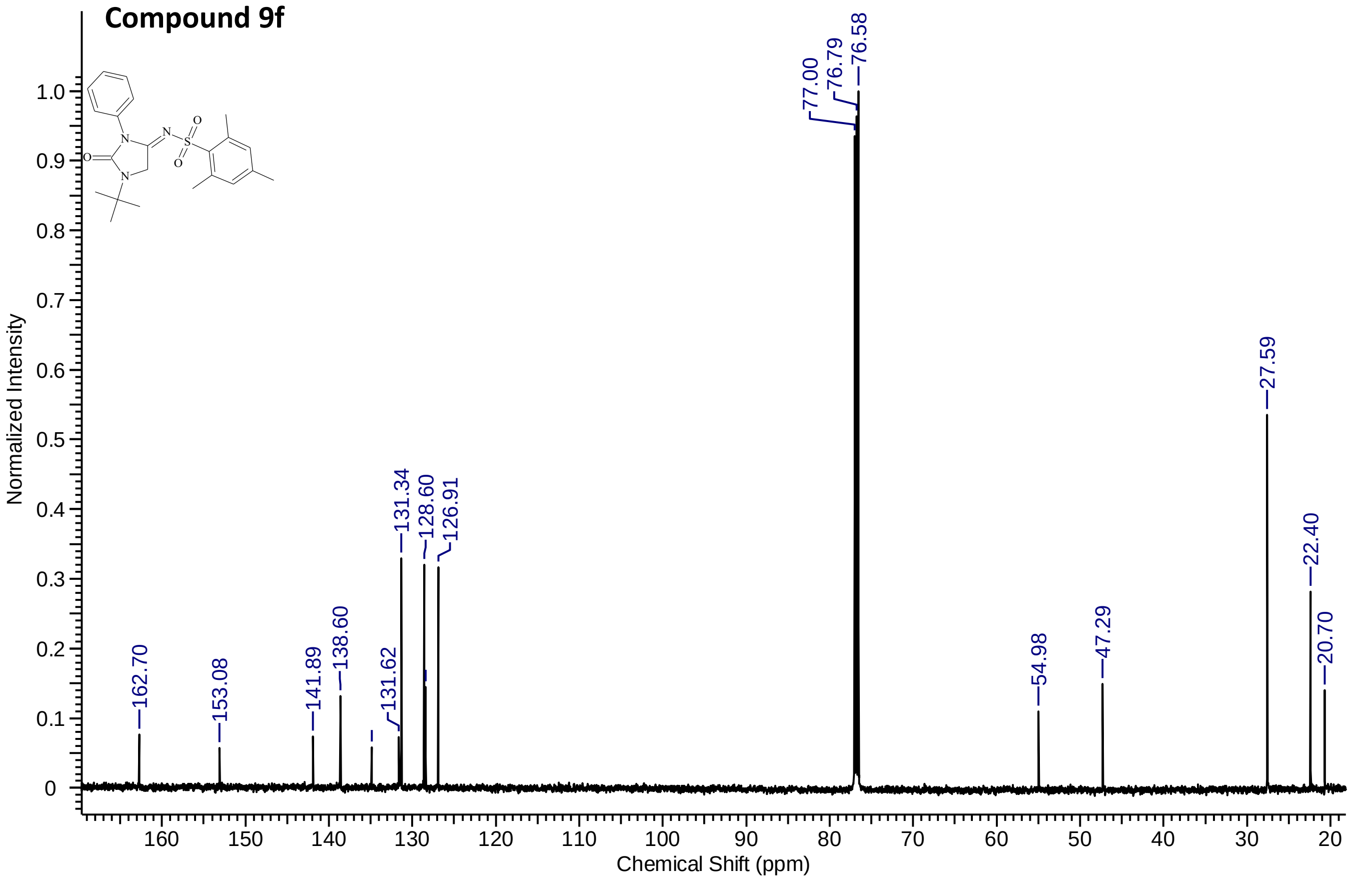
Compound 9f
Normalized Intensity
Chemical Shift (ppm)
162.70
153.08
141.89
138.60
131.62
131.34
128.60
126.91
77.00
76.79
76.58
54.98
47.29
27.59
22.40
20.70

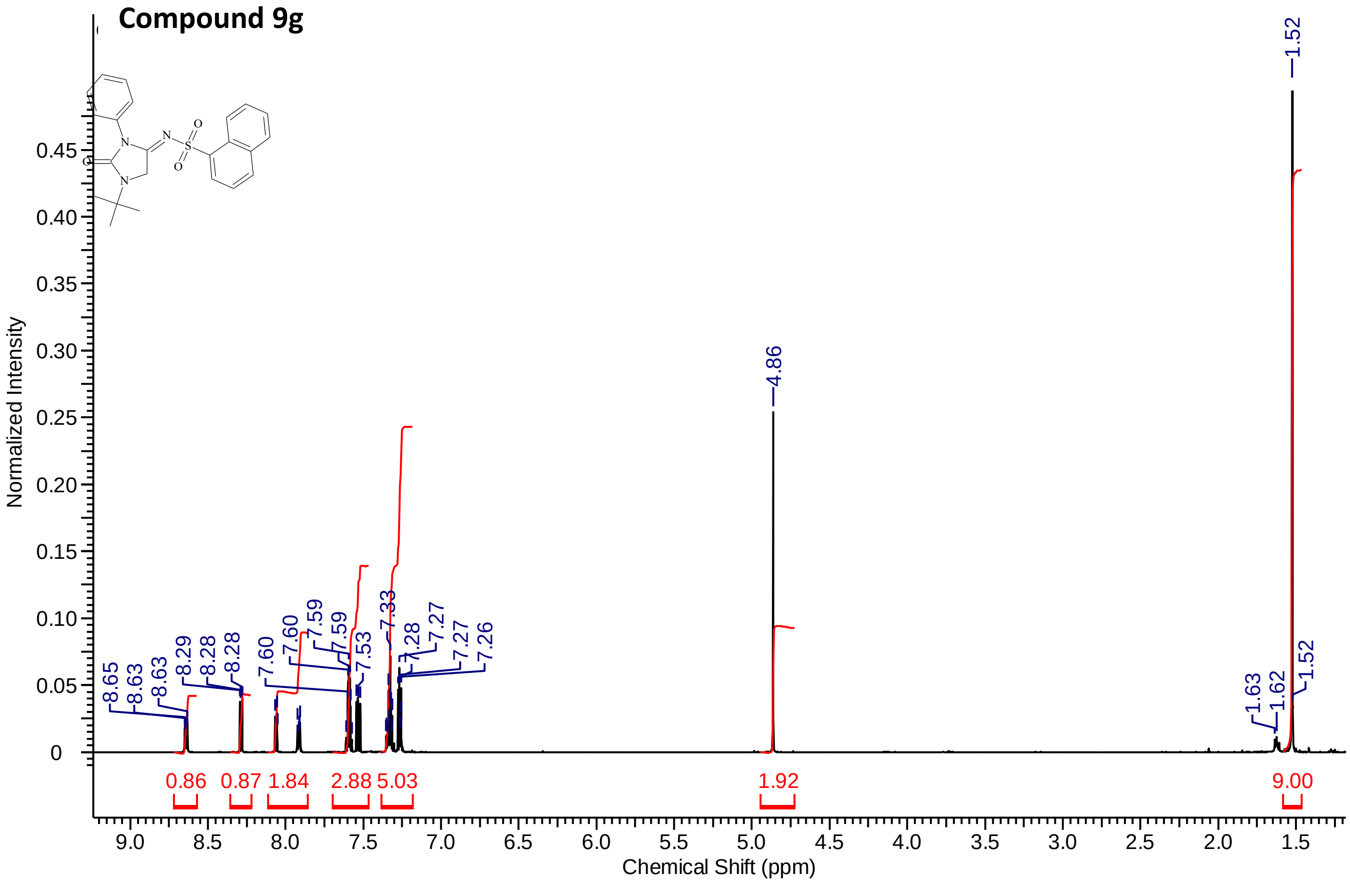
Compound 9g
Normalized Intensity
Chemical Shift (ppm)
8.65 8.63 8.63 8.29 8.28 8.28 7.60 7.60 7.59 7.59 7.53 7.33 7.28 7.27 7.27 7.26
4.86
1.63 1.62 1.52 1.52
0.86 0.87 1.84 2.88 5.03 1.92 9.00

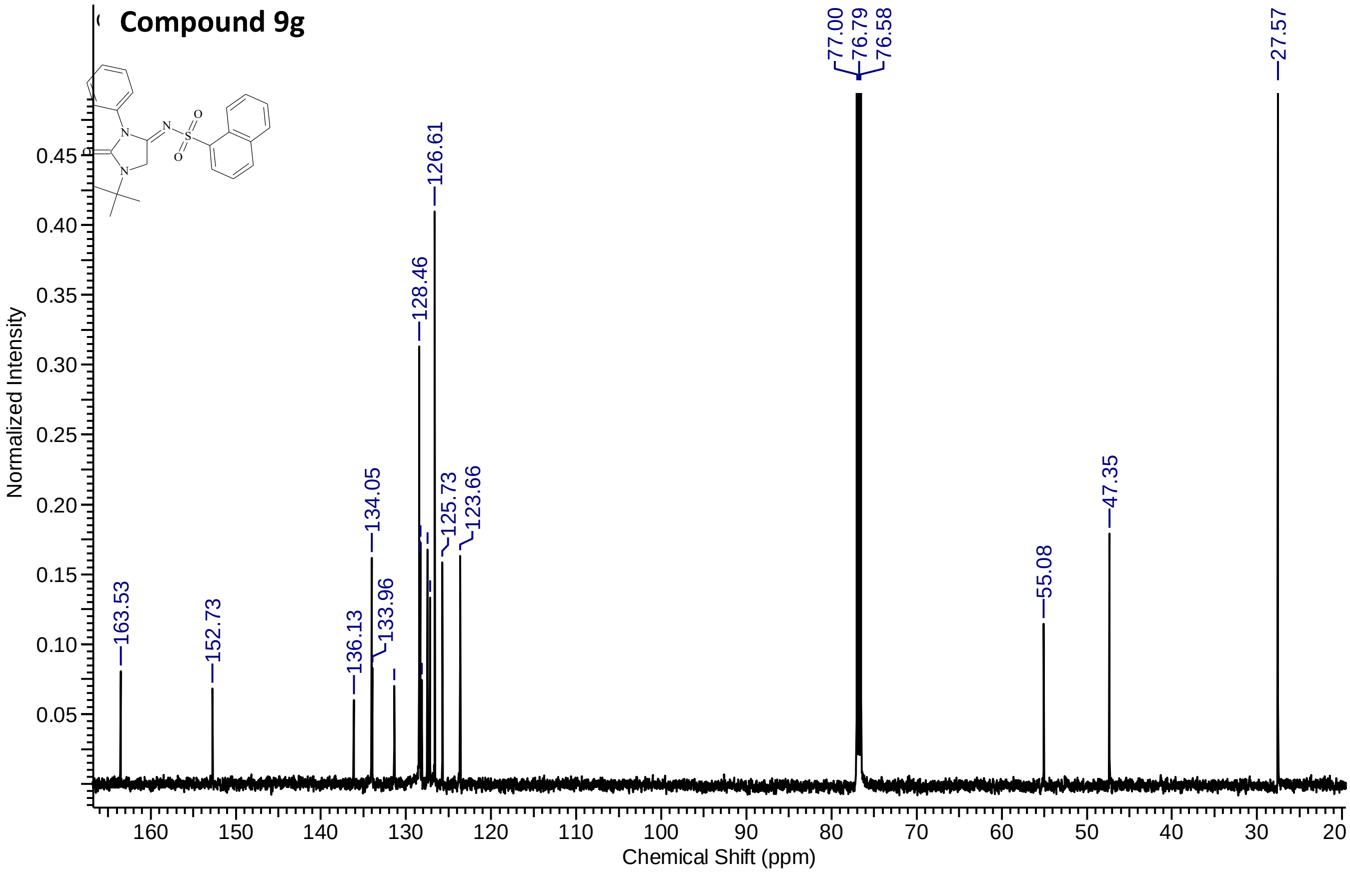
Compound 9g
Normalized Intensity
Chemical Shift (ppm)
163.53
152.73
136.13 134.05 133.96 128.46 126.61 125.73 123.66
77.00 76.79 76.58
55.08 47.35 27.57

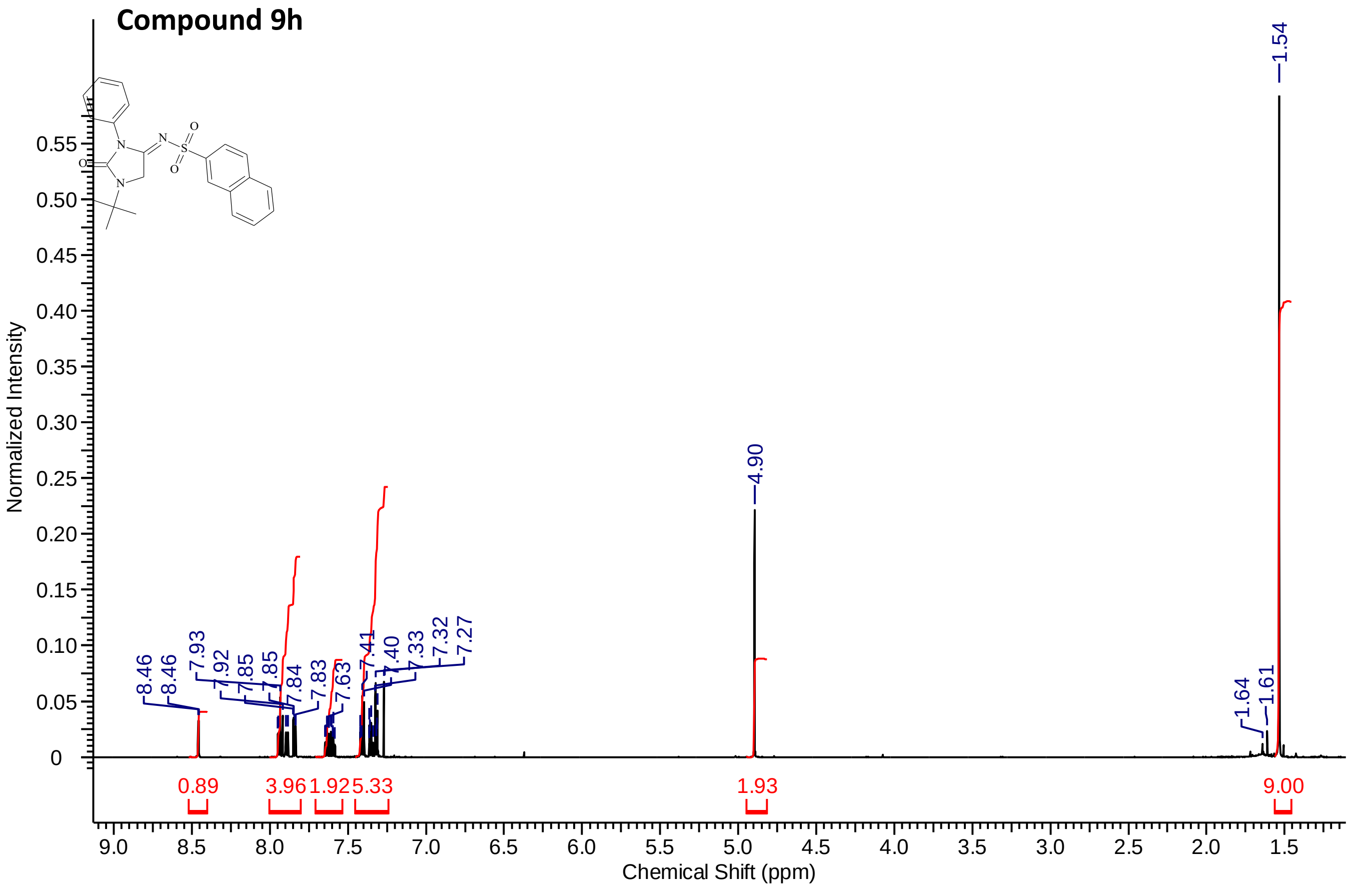
Compound 9h
8.46
8.46
7.93
7.92
7.85
7.85
7.84
7.83
7.63
7.41
7.40
7.33
7.32
7.27
4.90
1.64
1.61
1.54
0.89
3.96
1.92
5.33
1.93
9.00
Normalized Intensity
Chemical Shift (ppm)

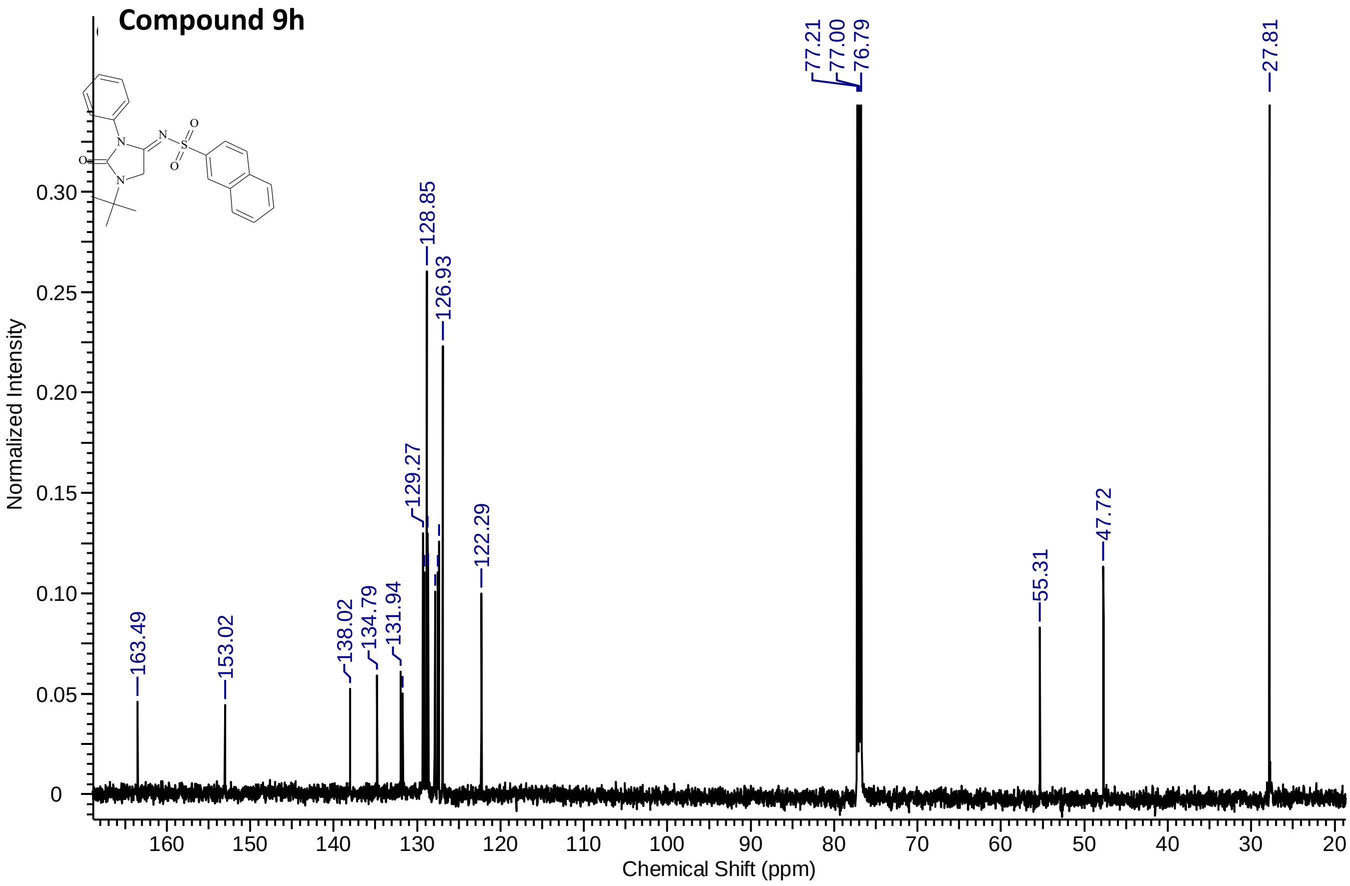
Compound 9h
163.49
153.02
138.02
134.79
131.94
129.27
128.85
126.93
122.29
77.21
77.00
76.79
55.31
47.72
27.81
Normalized Intensity
Chemical Shift (ppm)

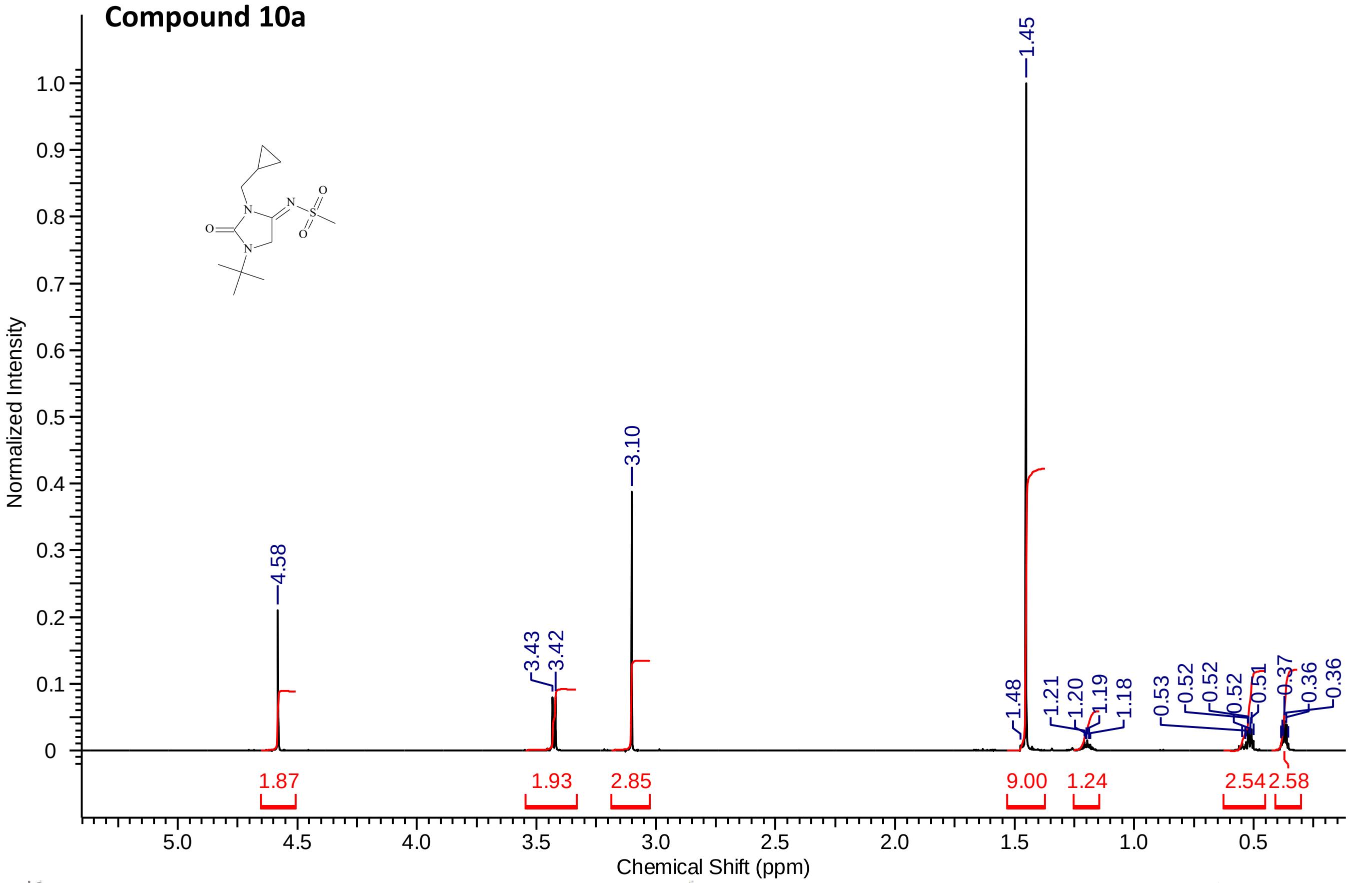
Compound 10a
Normalized Intensity
Chemical Shift (ppm)
4.58
3.43
3.42
3.10
1.48
1.45
1.21
1.20
1.19
1.18
0.53
0.52
0.52
0.52
0.51
0.37
0.36
0.36
1.87
1.93
2.85
9.00
1.24
2.54
2.58

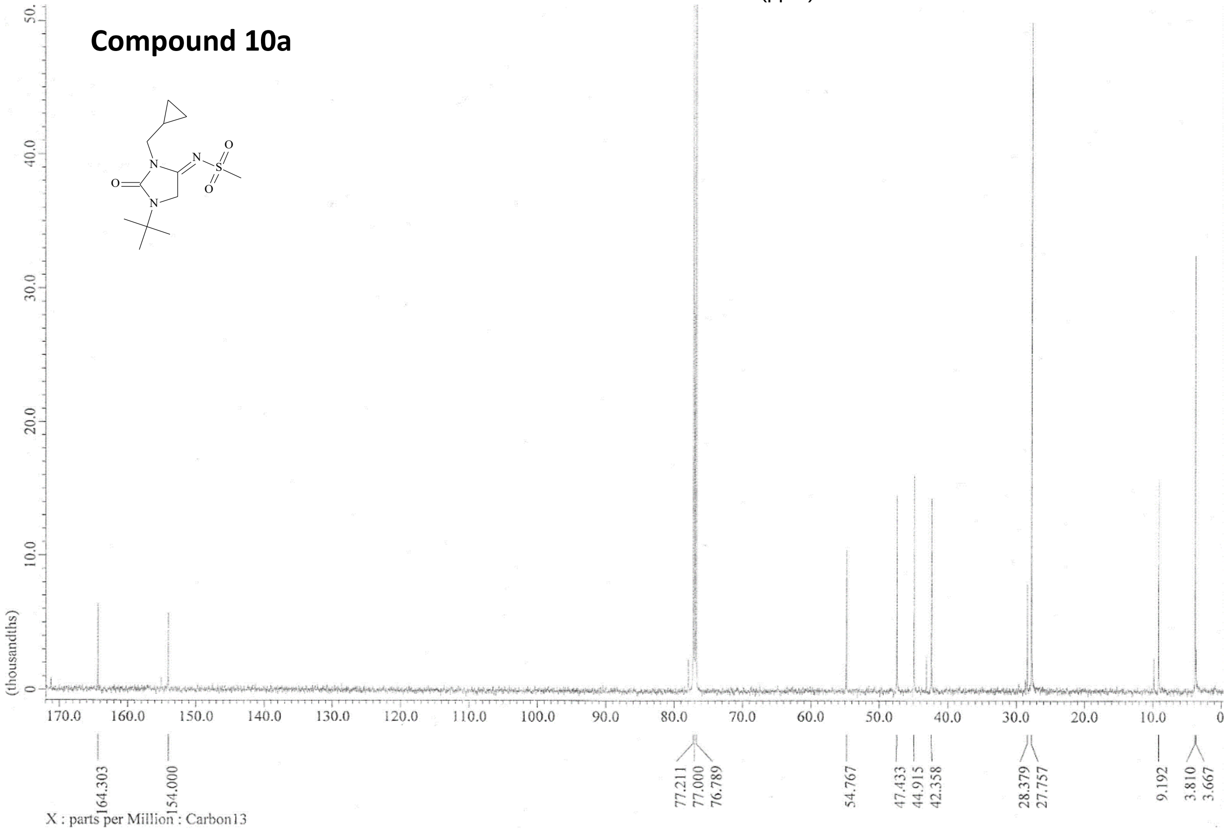
Compound 10a
(thousandths)
164.303
154.000
77.211
77.000
76.789
54.767
47.433
44.915
42.358
28.379
27.757
9.192
3.810
3.667
X : parts per Million : Carbon13

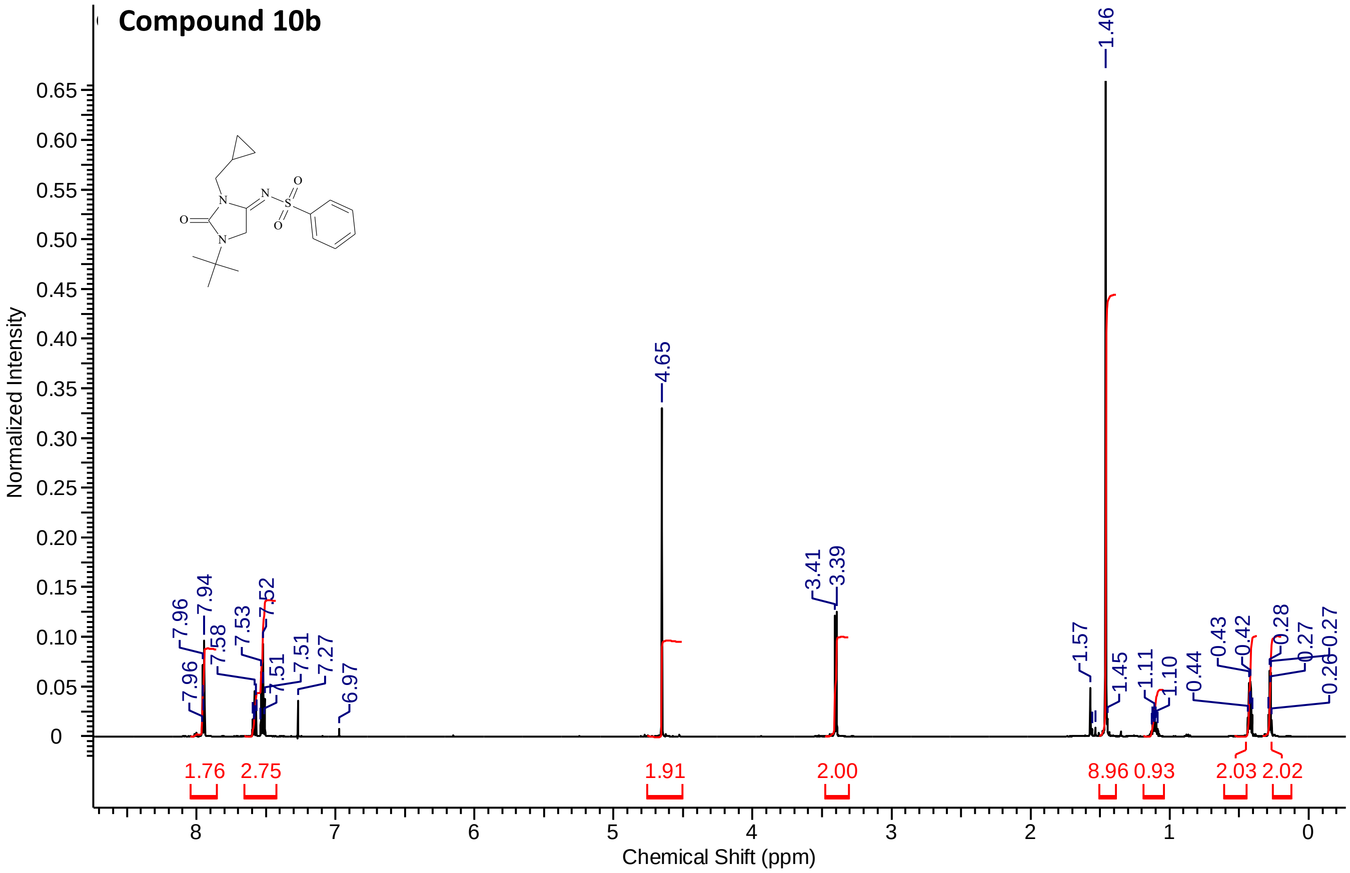

Compound 10b
Normalized Intensity
Chemical Shift (ppm)
1.76
2.75
1.91
2.00
8.96
0.93
2.03
2.02

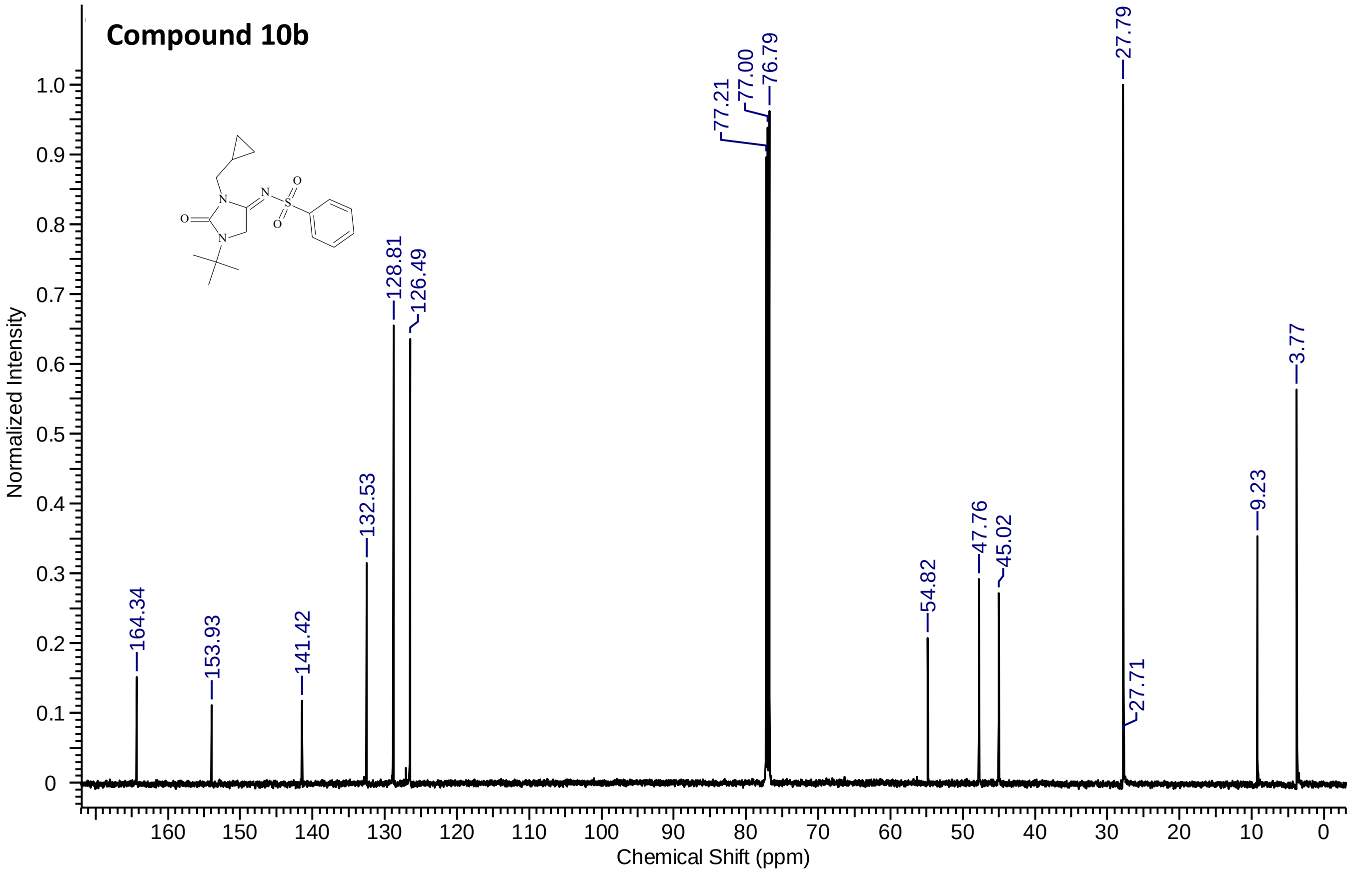

Compound 10b
Normalized Intensity
Chemical Shift (ppm)

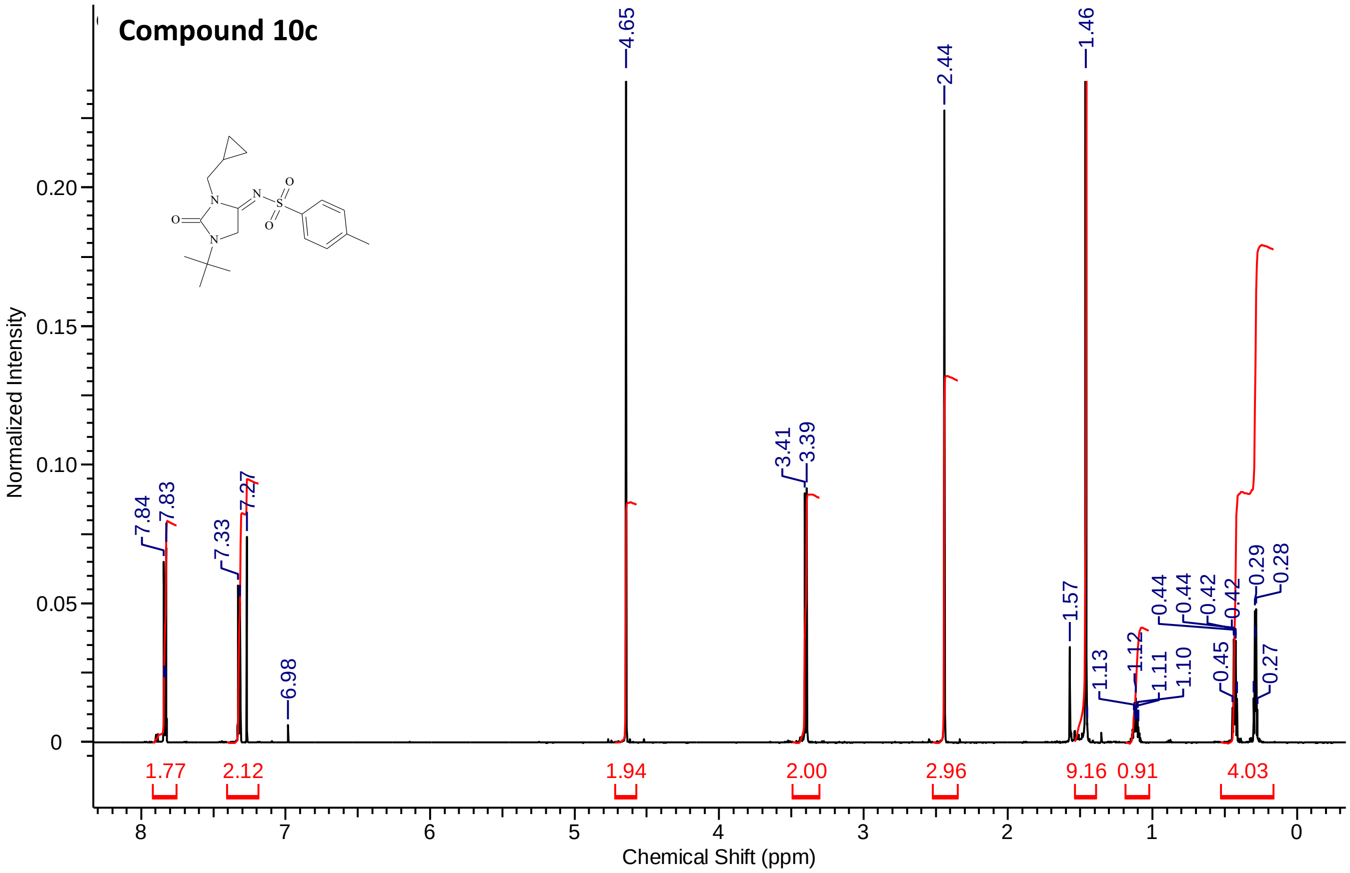
Compound 10c
Normalized Intensity
Chemical Shift (ppm)
7.84
7.83
7.33
7.27
6.98
4.65
3.41
3.39
2.44
1.57
1.46
1.13
1.12
1.11
1.10
0.45
0.44
0.44
0.42
0.42
0.29
0.28
0.27
1.77
2.12
1.94
2.00
2.96
9.16
0.91
4.03

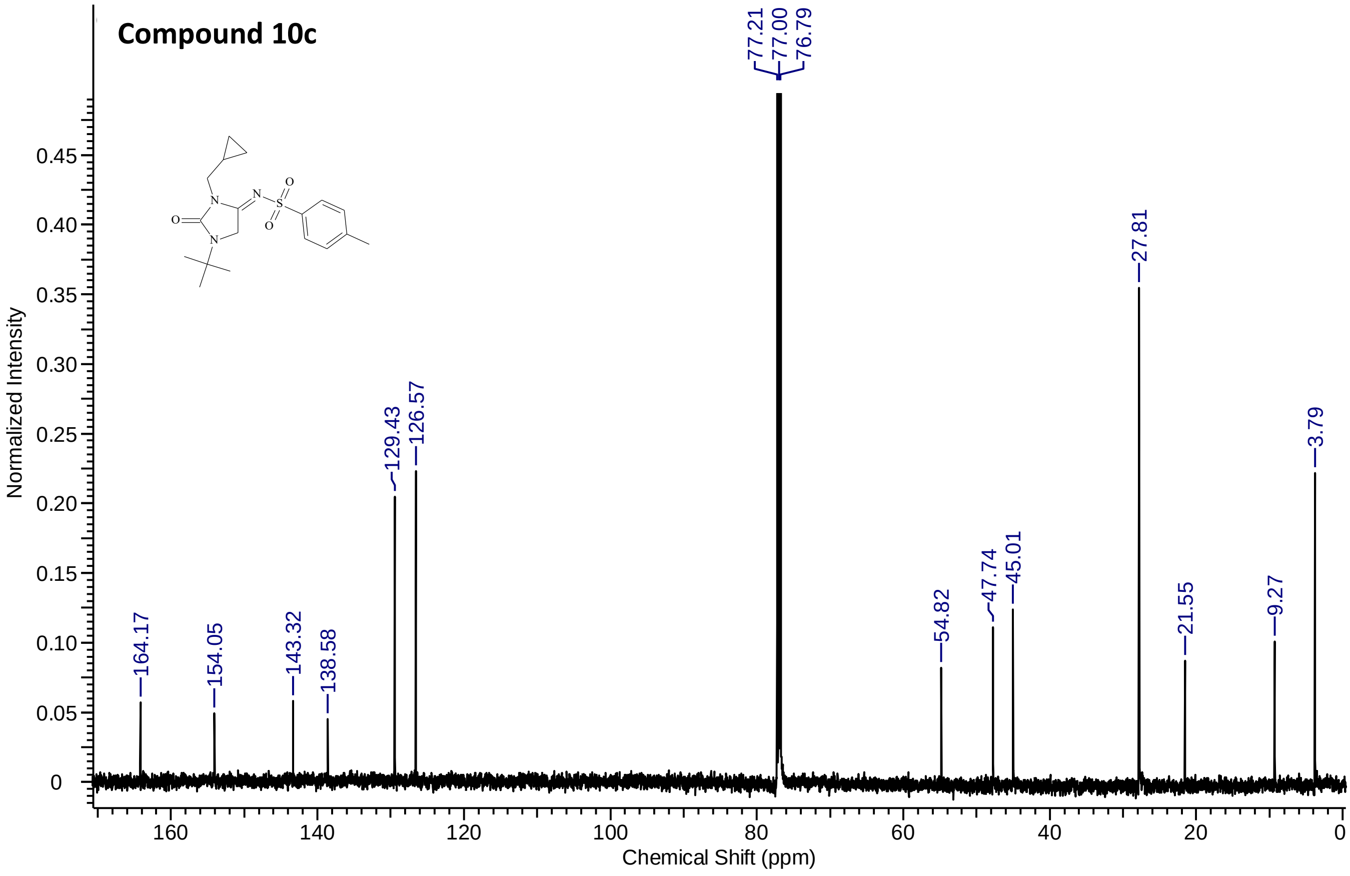
Compound 10c
Normalized Intensity
Chemical Shift (ppm)
164.17
154.05
143.32
138.58
129.43
126.57
77.21
77.00
76.79
54.82
47.74
45.01
27.81
21.55
9.27
3.79

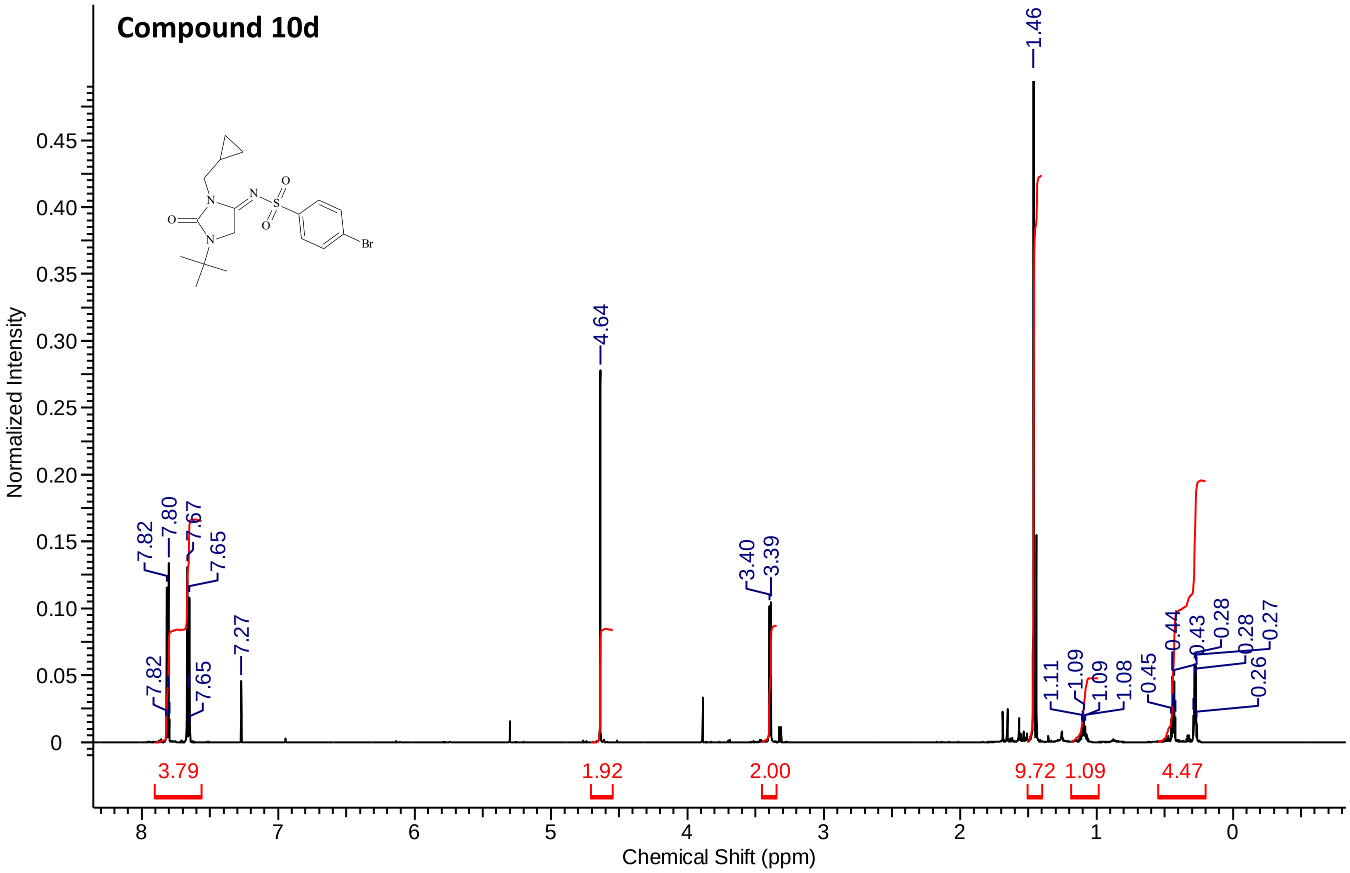
Compound 10d
Normalized Intensity
Chemical Shift (ppm)
7.82 7.80 7.67 7.65 7.82 7.65 7.27 4.64 3.40 3.39 1.46 1.11 1.09 1.09 1.08 0.45 0.44 0.43 0.28 0.28 0.27 0.26
3.79 1.92 2.00 9.72 1.09 4.47
Br

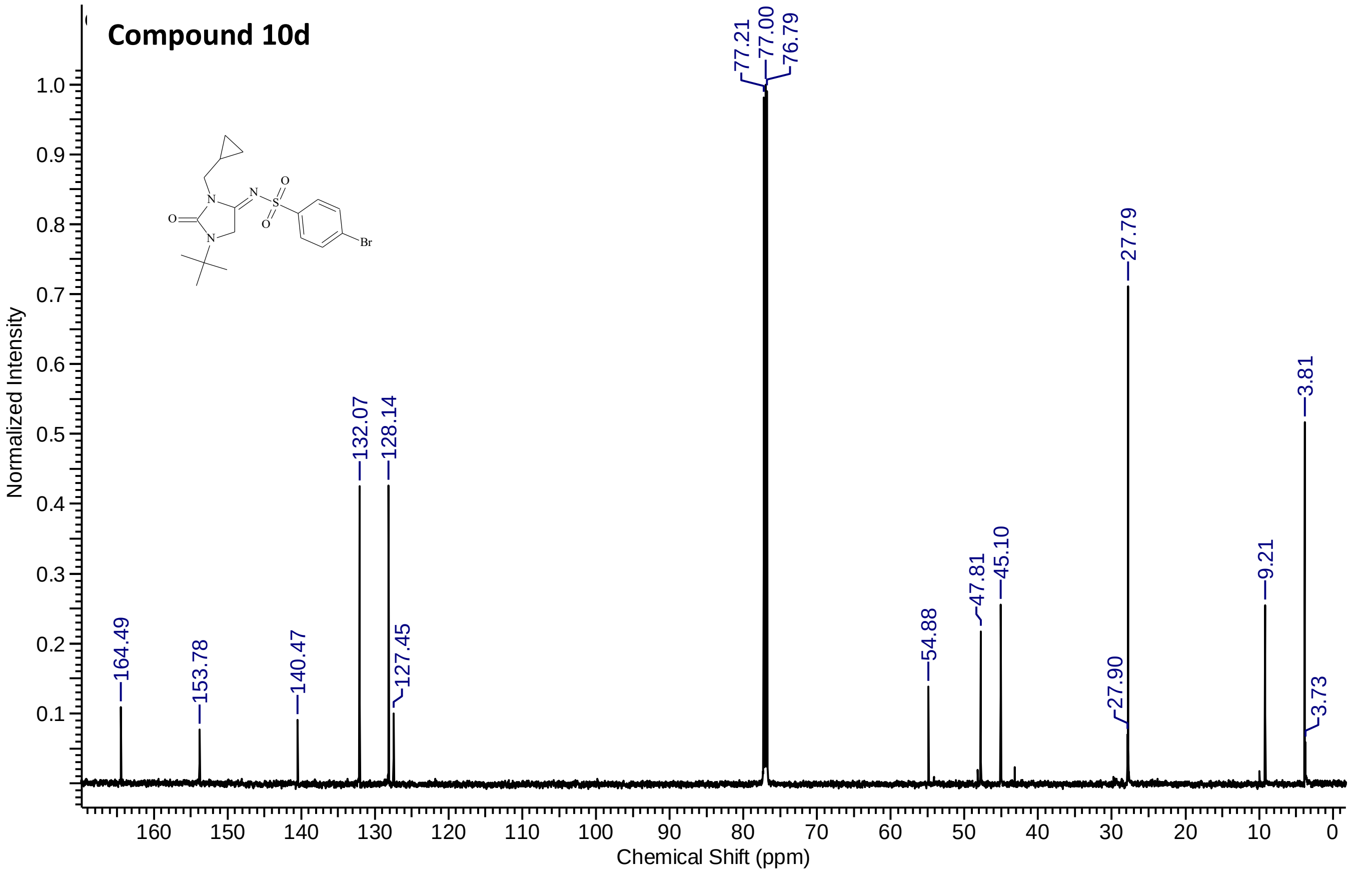
Compound 10d
Normalized Intensity
Chemical Shift (ppm)
164.49 153.78 140.47 132.07 128.14 127.45 77.21 77.00 76.79 54.88 47.81 45.10 27.90 27.79 9.21 3.81 3.73
Br

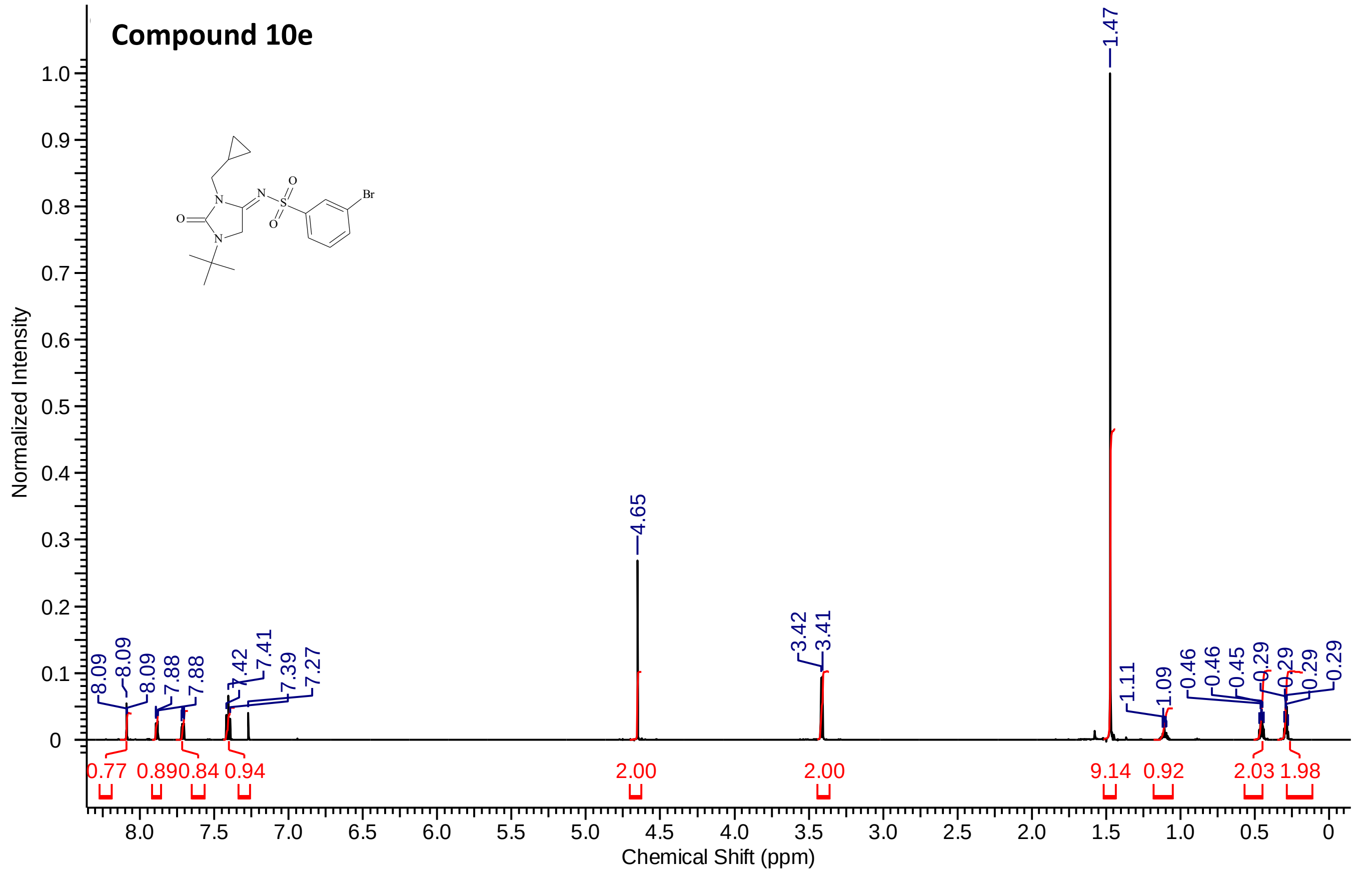
Compound 10e
Br
N
O
S
Normalized Intensity
Chemical Shift (ppm)
8.09 8.09 8.09 7.88 7.88 7.42 7.41 7.39 7.27
4.65
3.42 3.41
1.47
1.11 1.09 0.46 0.46 0.45 0.29 0.29 0.29 0.29
0.77 0.89 0.84 0.94 2.00 2.00 9.14 0.92 2.03 1.98

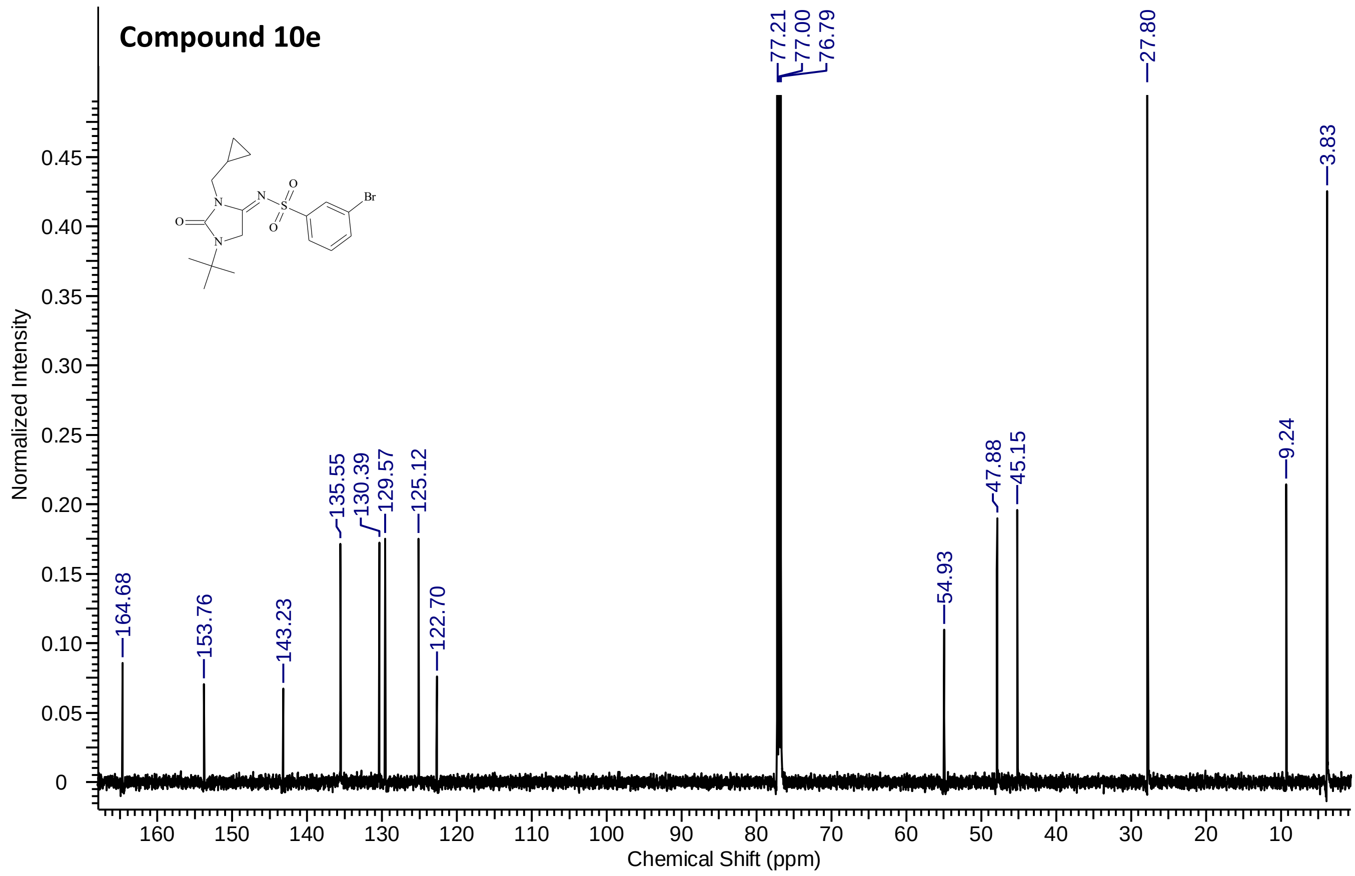
Compound 10e
Br
N
O
S
Normalized Intensity
Chemical Shift (ppm)
164.68
153.76
143.23
135.55 130.39 129.57
125.12
122.70
77.21 77.00 76.79
54.93
47.88 45.15
27.80
9.24
3.83

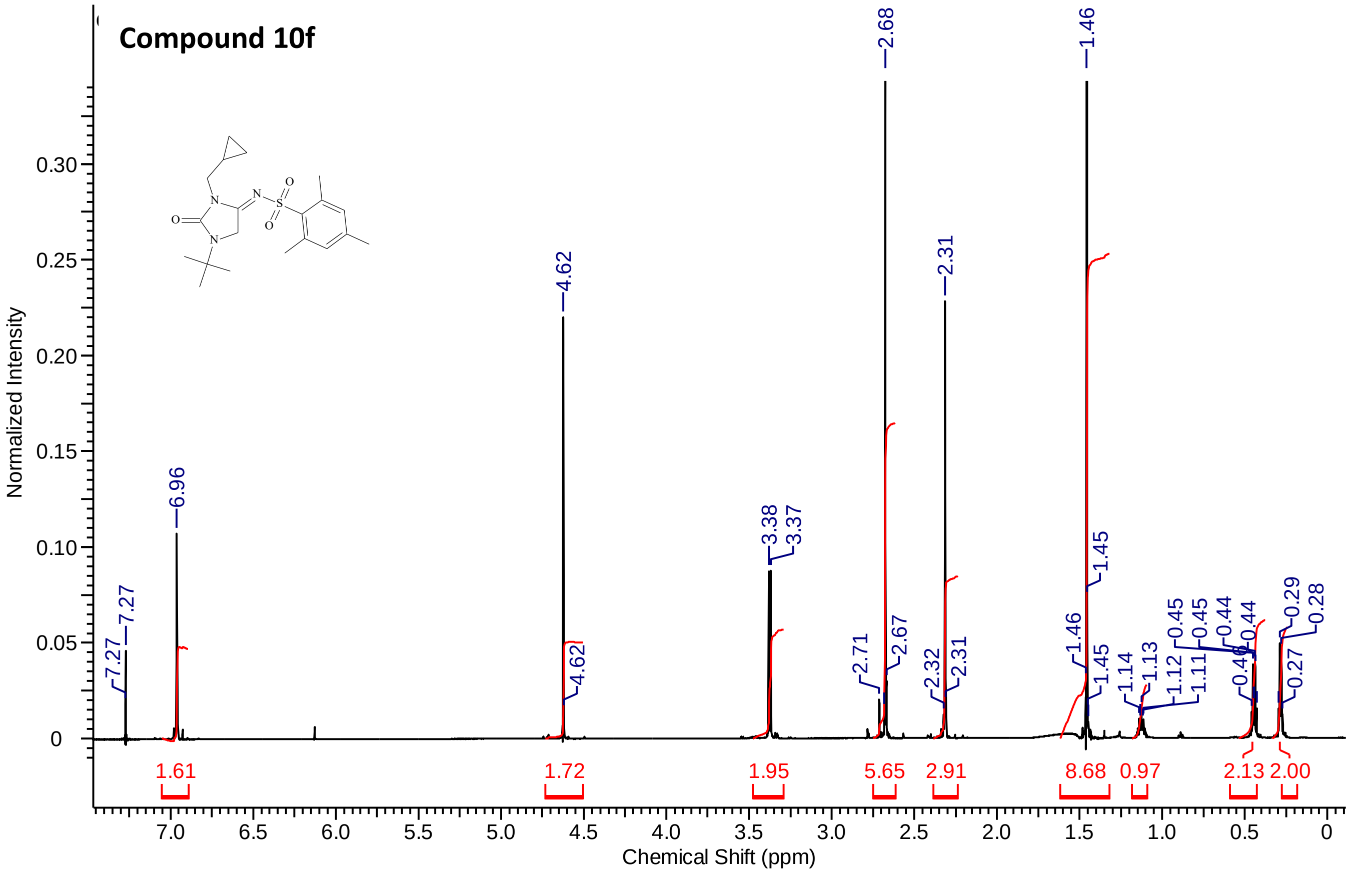
Compound 10f
Normalized Intensity
Chemical Shift (ppm)
1.61
1.72
1.95
5.65
2.91
8.68
0.97
2.13
2.00

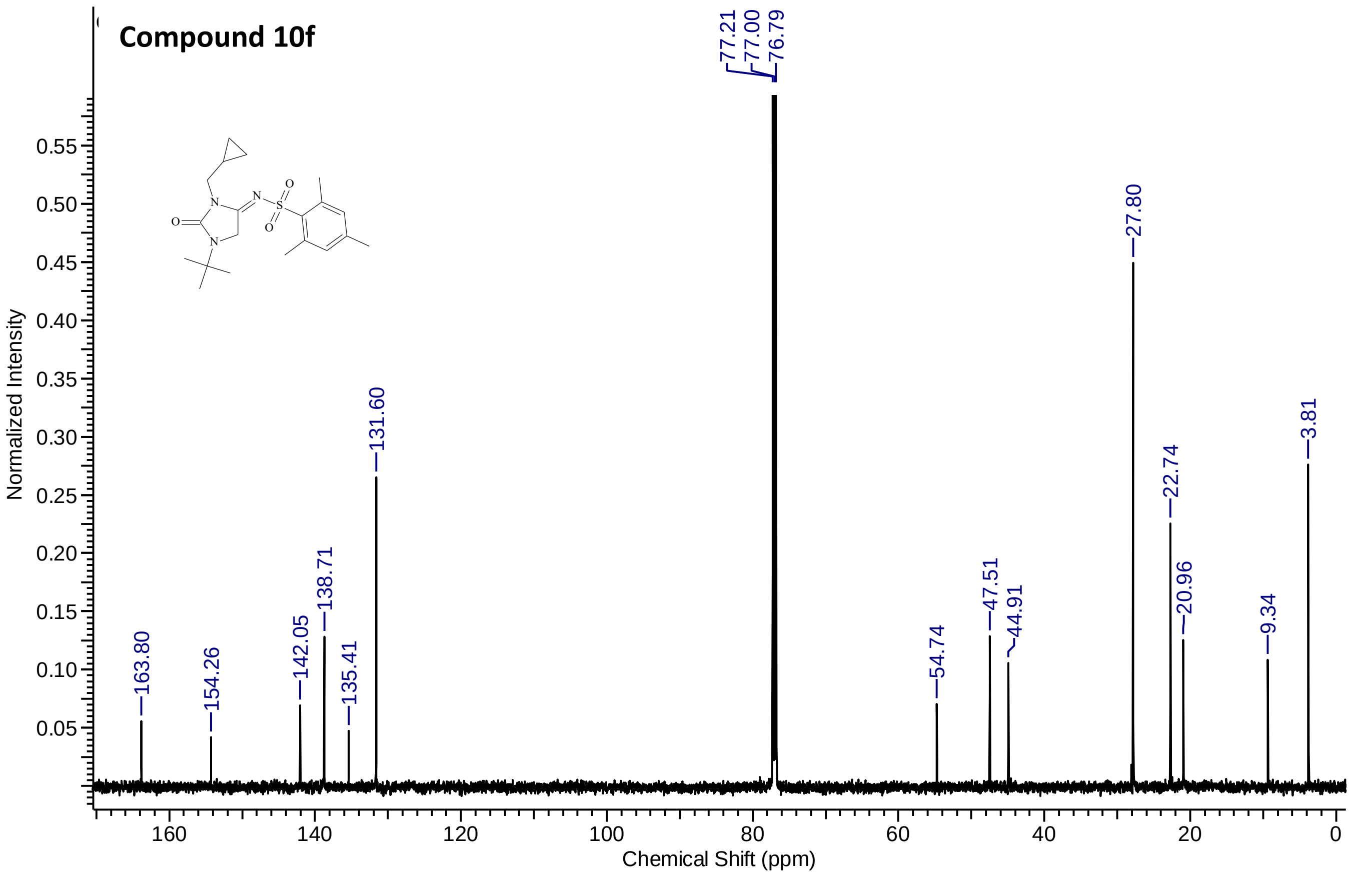
Compound 10f
Normalized Intensity
Chemical Shift (ppm)
163.80
154.26
142.05
138.71
135.41
131.60
77.21
77.00
76.79
54.74
47.51
44.91
27.80
22.74
20.96
9.34
3.81

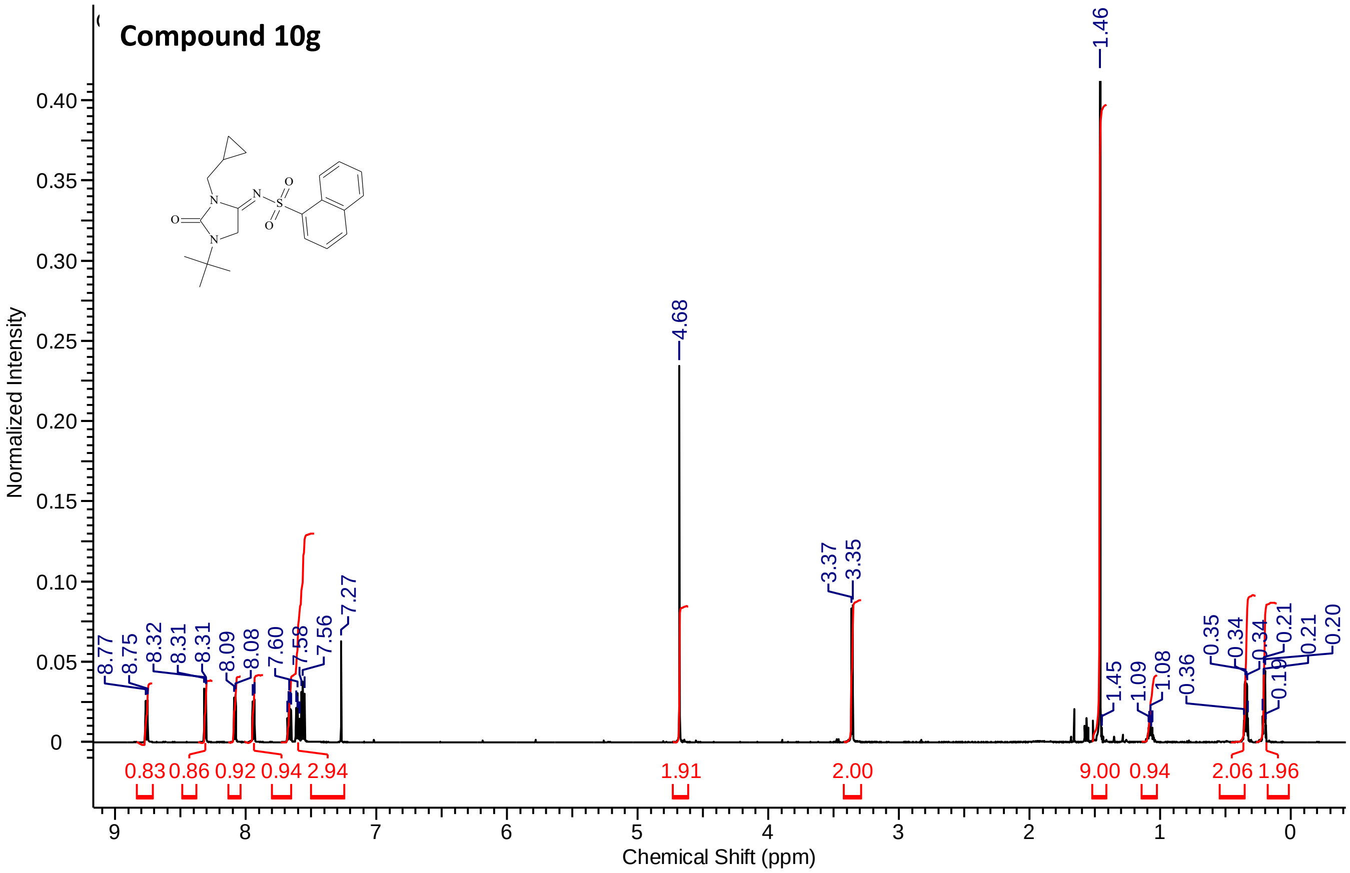
Compound 10g
Normalized Intensity
Chemical Shift (ppm)
8.77
8.75
8.32
8.31
8.31
8.09
8.08
7.60
7.58
7.56
7.27
4.68
3.37
3.35
1.46
1.45
1.09
1.08
0.36
0.35
0.34
0.34
0.21
0.21
0.20
0.19
0.83
0.86
0.92
0.94
2.94
1.91
2.00
9.00
0.94
2.06
1.96

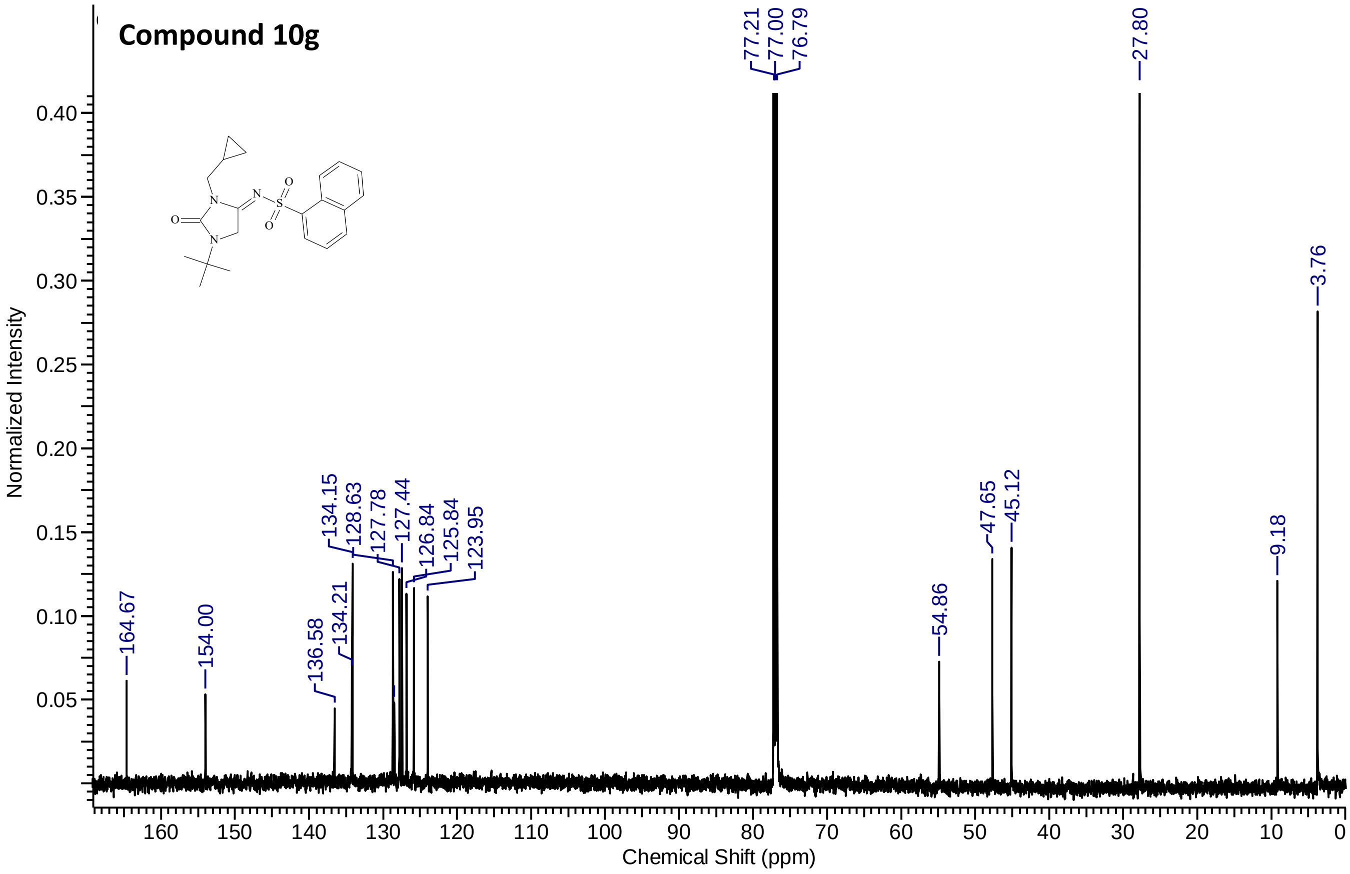
Compound 10g
Normalized Intensity
Chemical Shift (ppm)
164.67
154.00
136.58
134.21
134.15
128.63
127.78
127.44
126.84
125.84
123.95
77.21
77.00
76.79
54.86
47.65
45.12
27.80
9.18
3.76

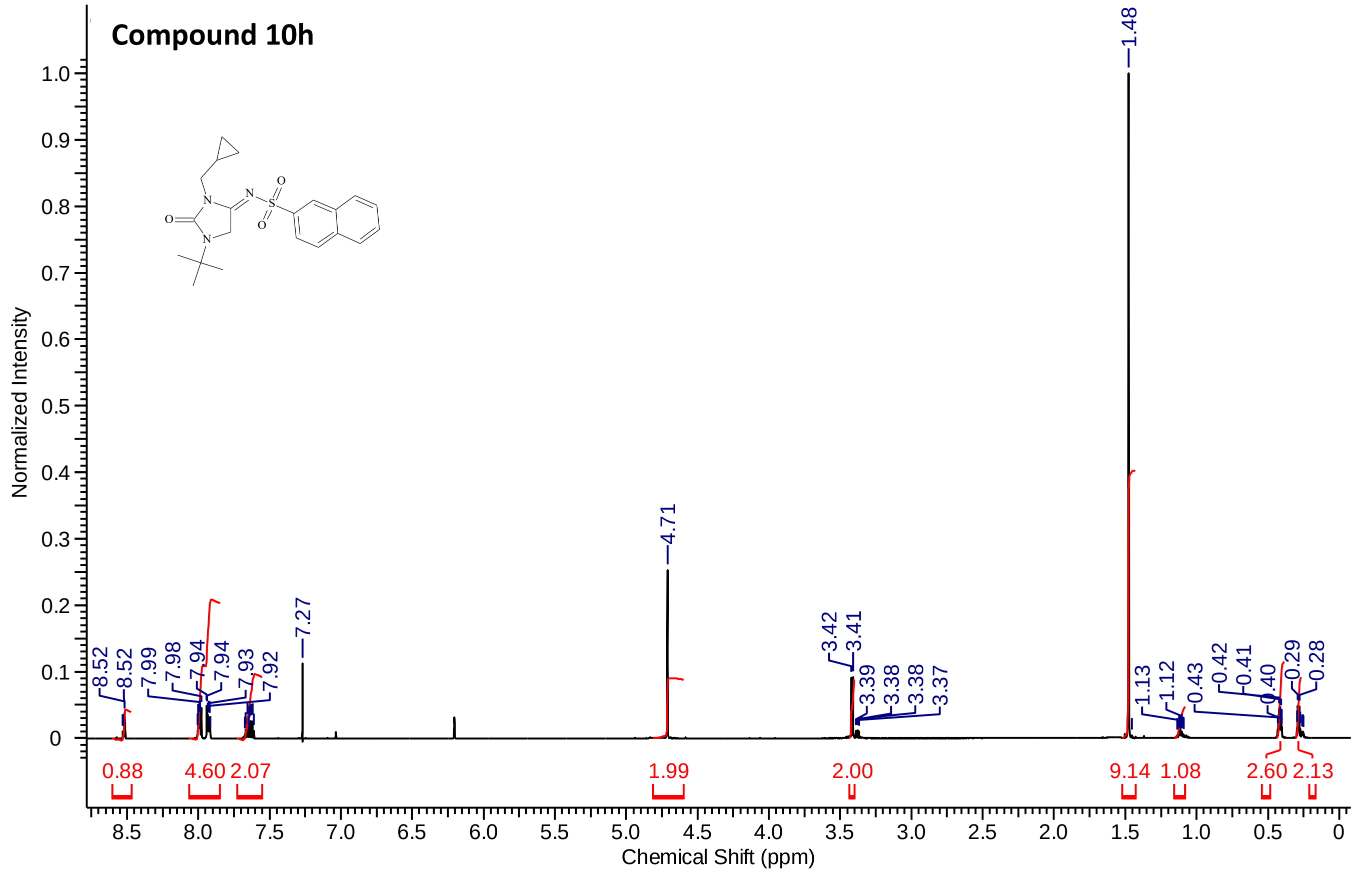
Compound 10h
Normalized Intensity
Chemical Shift (ppm)
8.52
8.52
7.99
7.98
7.94
7.94
7.93
7.92
7.27
4.71
3.42
3.41
3.39
3.38
3.38
3.37
1.48
1.13
1.12
0.43
0.42
0.41
0.40
0.29
0.28
0.88
4.60
2.07
1.99
2.00
9.14
1.08
2.60
2.13

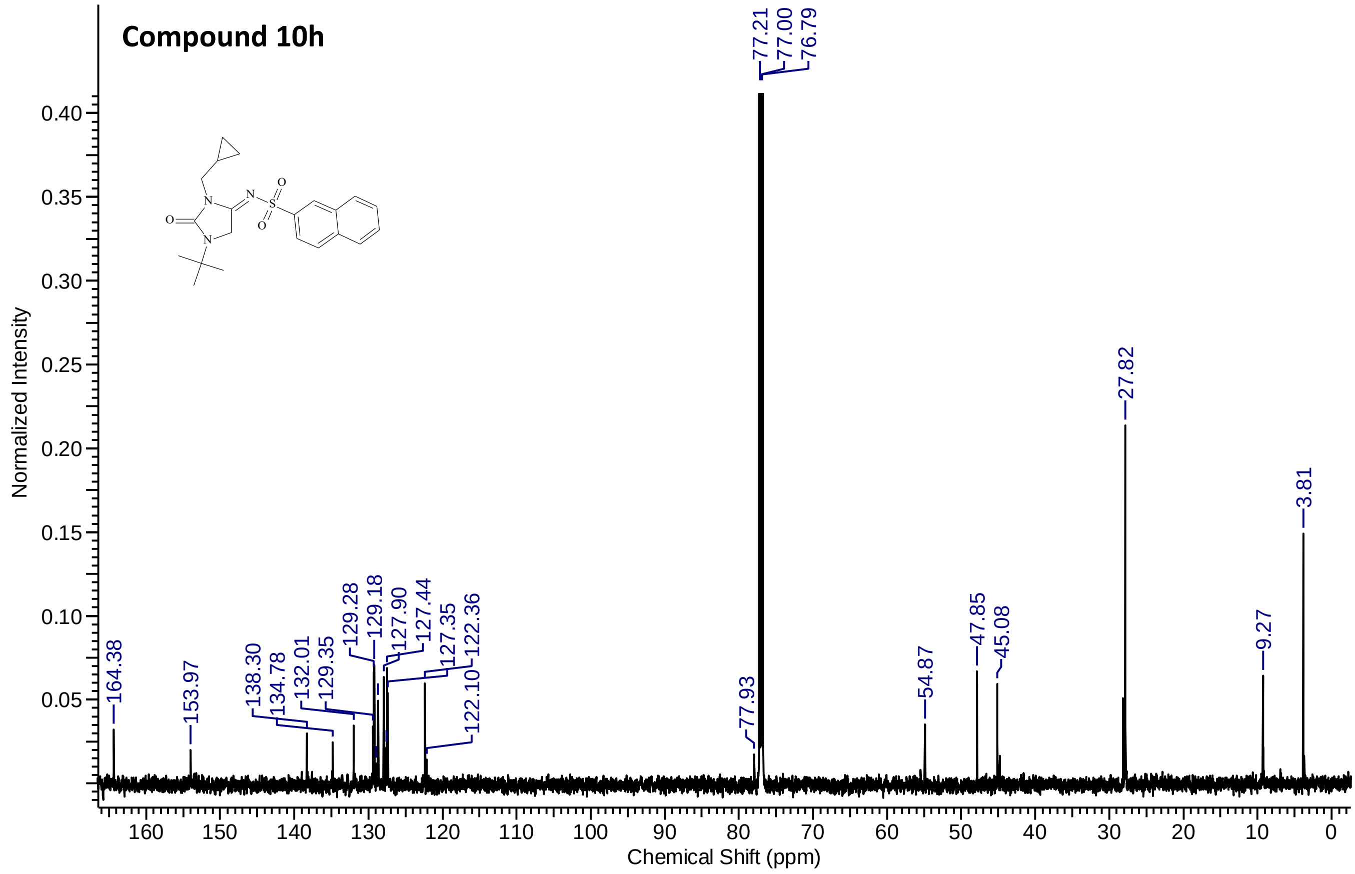
Compound 10h
Normalized Intensity
Chemical Shift (ppm)
164.38
153.97
138.30
134.78
132.01
129.35
129.28
129.18
127.90
127.44
127.35
122.36
122.10
77.93
77.21
77.00
76.79
54.87
47.85
45.08
27.82
9.27
3.81

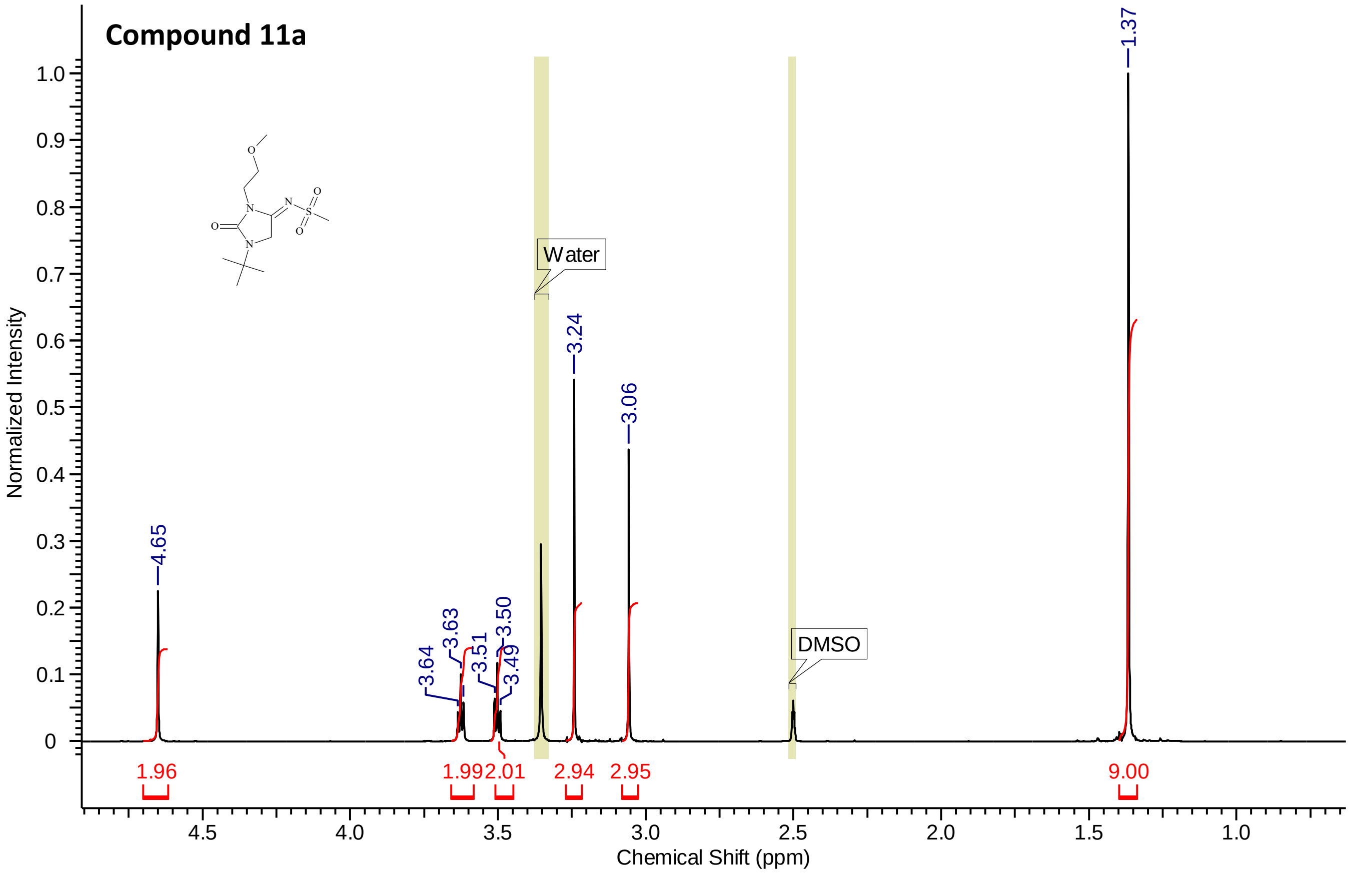
Compound 11a
Normalized Intensity
Chemical Shift (ppm)
Water
DMSO
4.65
3.64
3.63
3.51
3.50
3.49
3.24
3.06
1.37
1.96
1.99
2.01
2.94
2.95
9.00

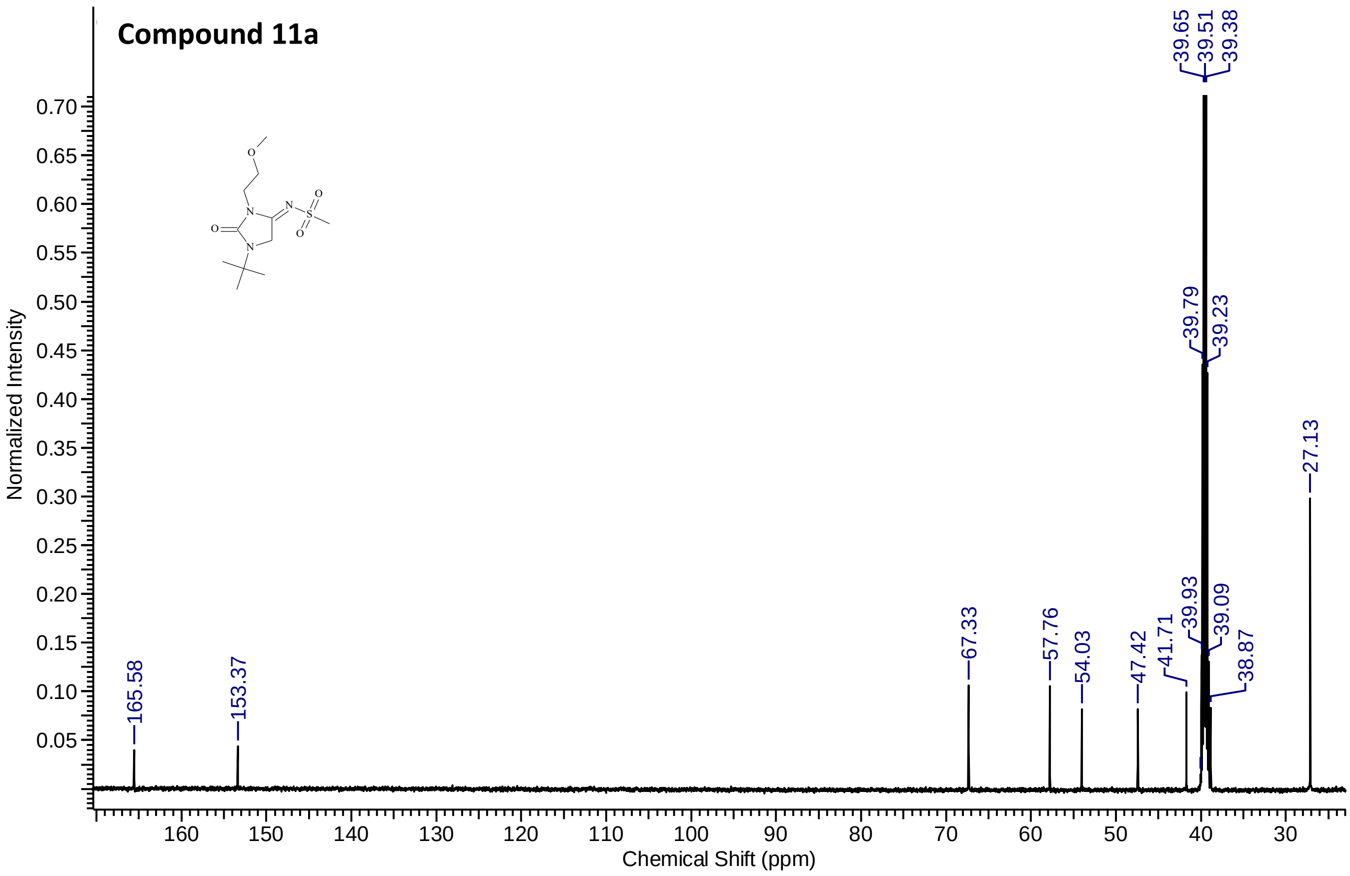
Compound 11a
Normalized Intensity
Chemical Shift (ppm)
165.58
153.37
67.33
57.76
54.03
47.42
41.71
39.93
39.79
39.65
39.51
39.38
39.23
39.09
38.87
27.13

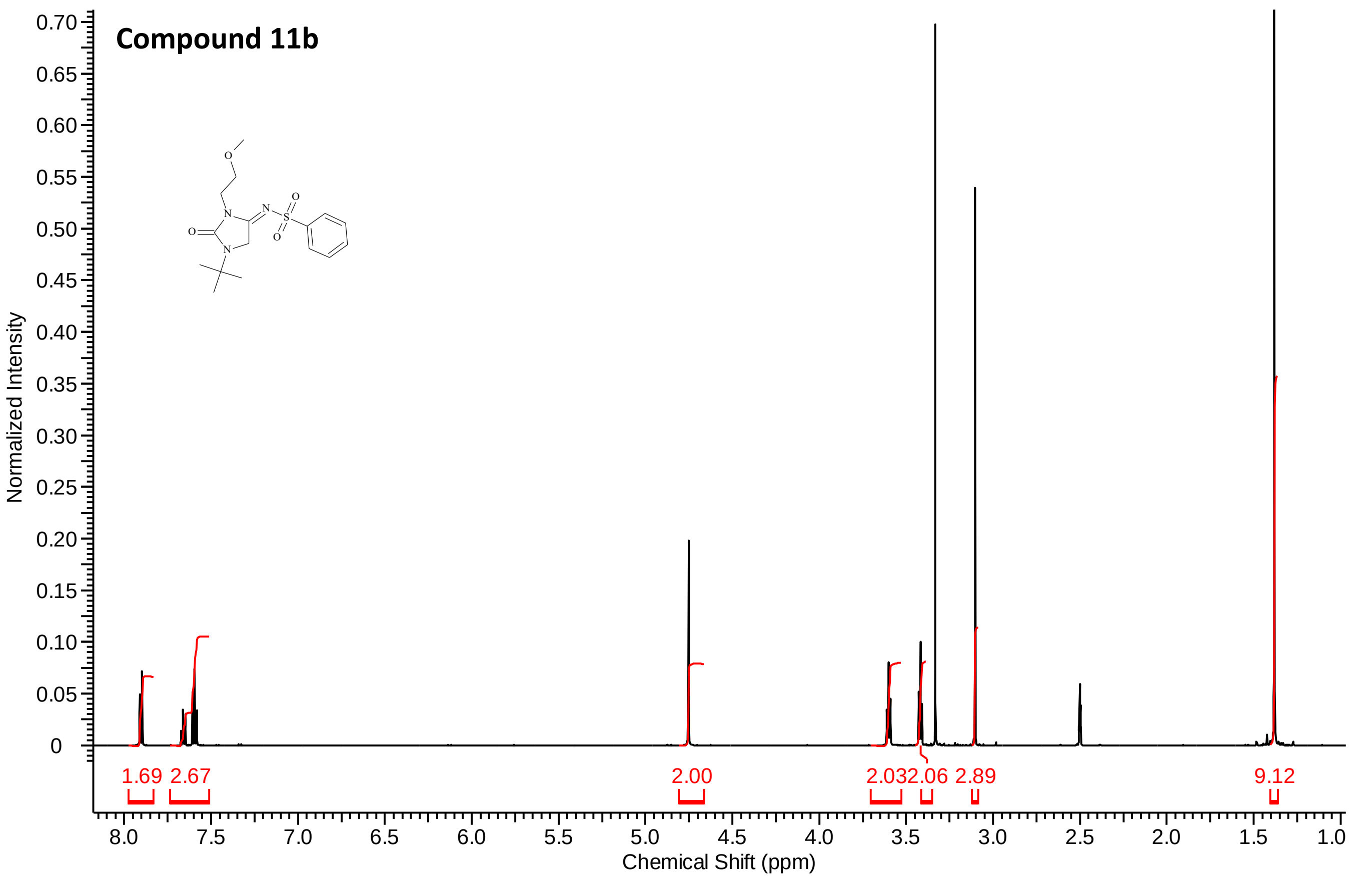

Compound 11b
Normalized Intensity
Chemical Shift (ppm)
1.69 2.67 2.00 2.03 2.06 2.89 9.12

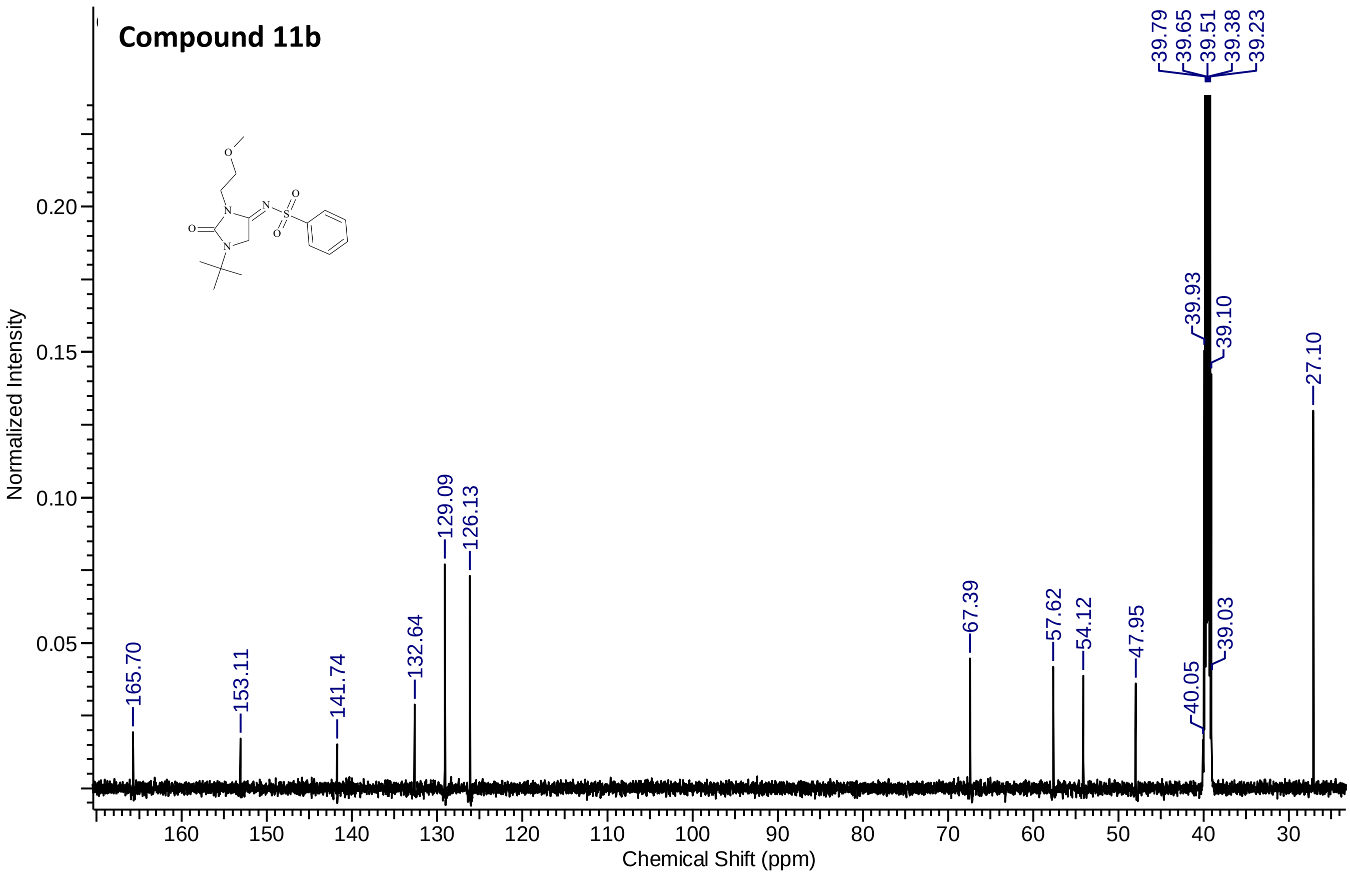

Compound 11b
Normalized Intensity
Chemical Shift (ppm)
165.70
153.11
141.74
132.64
129.09
126.13
67.39
57.62
54.12
47.95
40.05
39.93
39.79
39.65
39.51
39.38
39.23
39.10
39.03
27.10

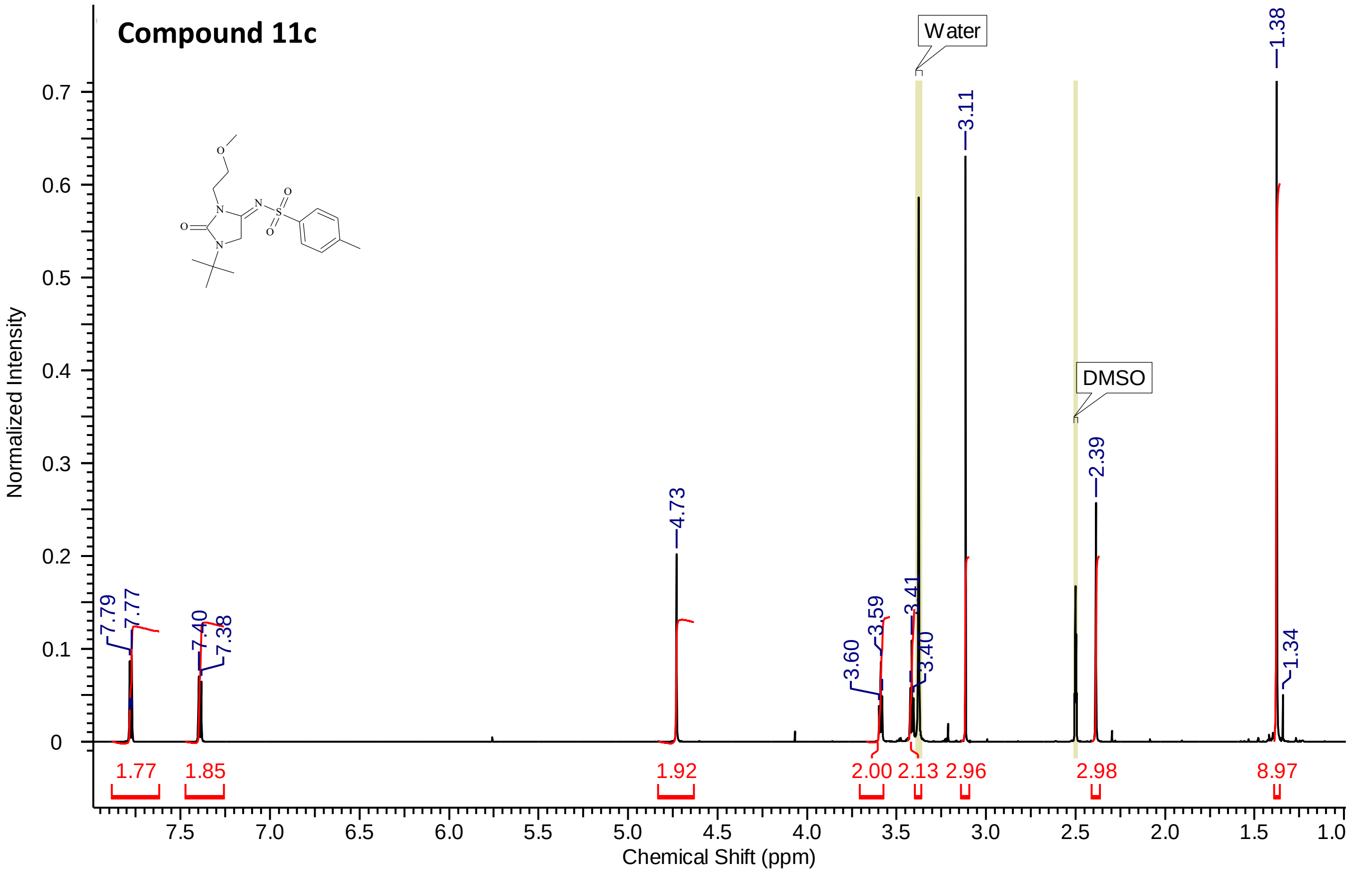
Compound 11c
Water
DMSO
7.79
7.77
7.40
7.38
4.73
3.60
3.59
3.41
3.40
3.11
2.39
1.38
1.34
1.77
1.85
1.92
2.00
2.13
2.96
2.98
8.97
Normalized Intensity
Chemical Shift (ppm)

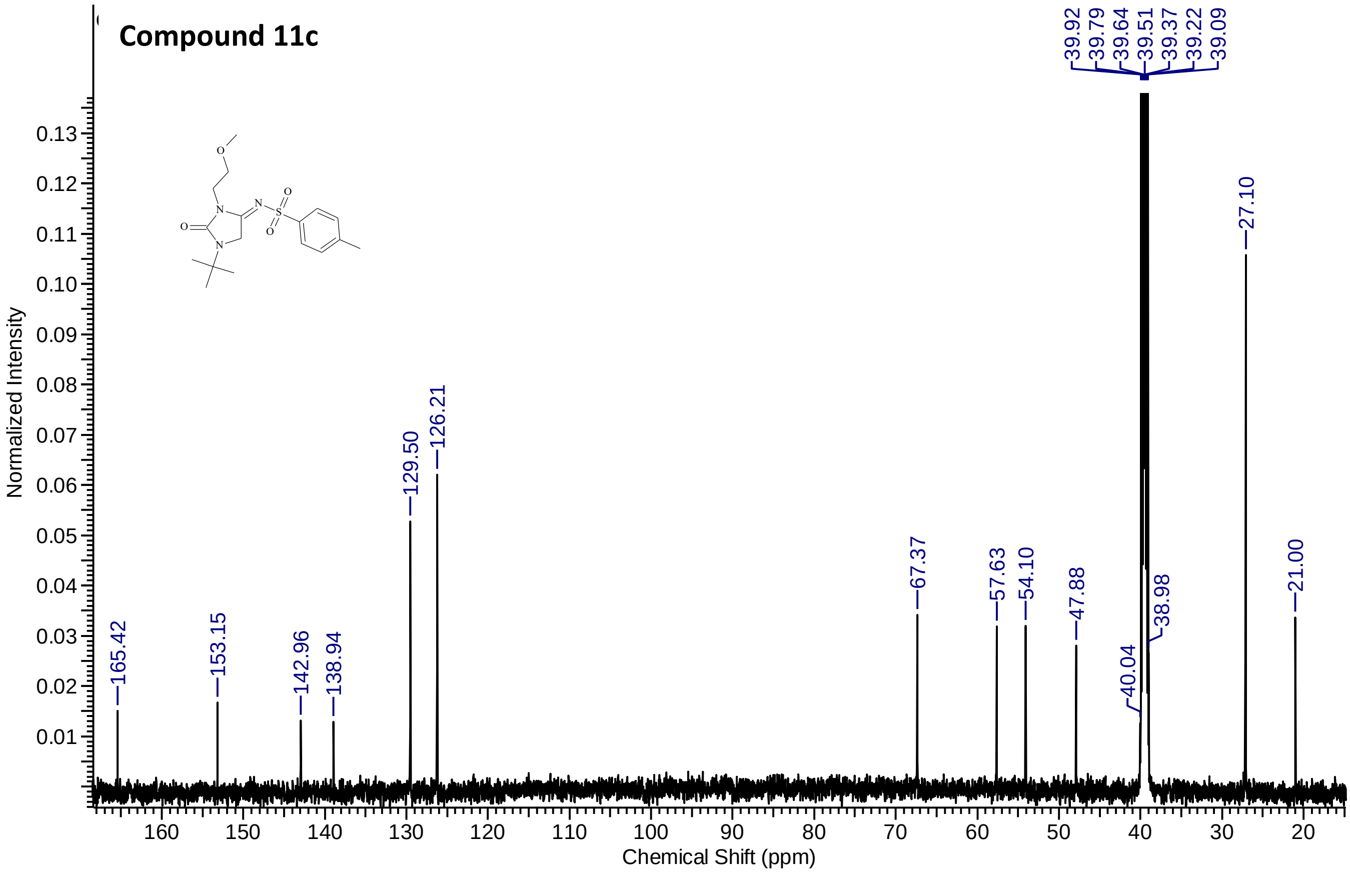
Compound 11c
39.92
39.79
39.64
39.51
39.37
39.22
39.09
165.42
153.15
142.96
138.94
129.50
126.21
67.37
57.63
54.10
47.88
40.04
38.98
27.10
21.00
Normalized Intensity
Chemical Shift (ppm)

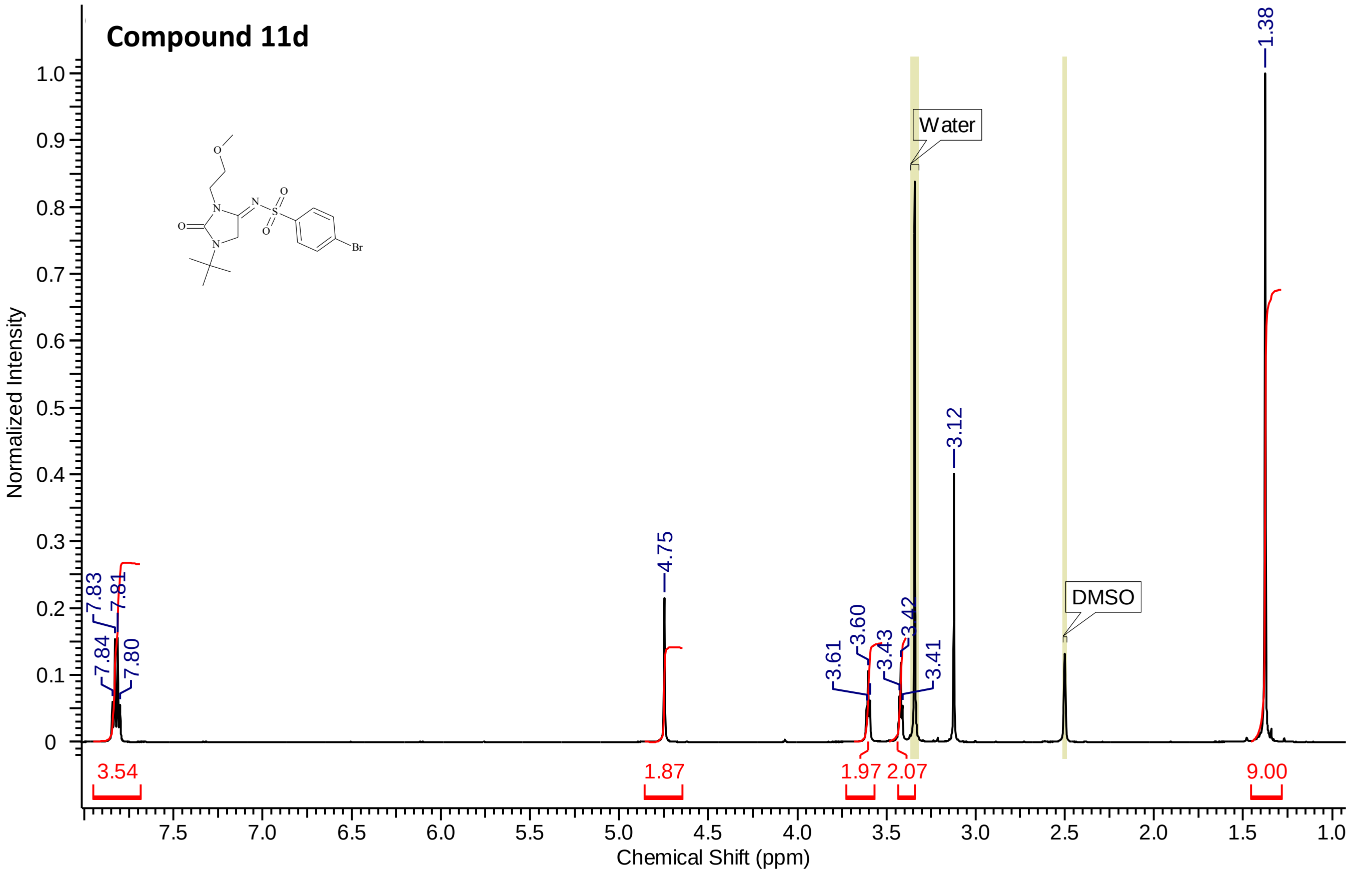

Compound 11d
Water
DMSO
7.83
7.84
7.81
7.80
4.75
3.61
3.60
3.43
3.42
3.41
3.12
1.38
3.54
1.87
1.97
2.07
9.00
Normalized Intensity
Chemical Shift (ppm)

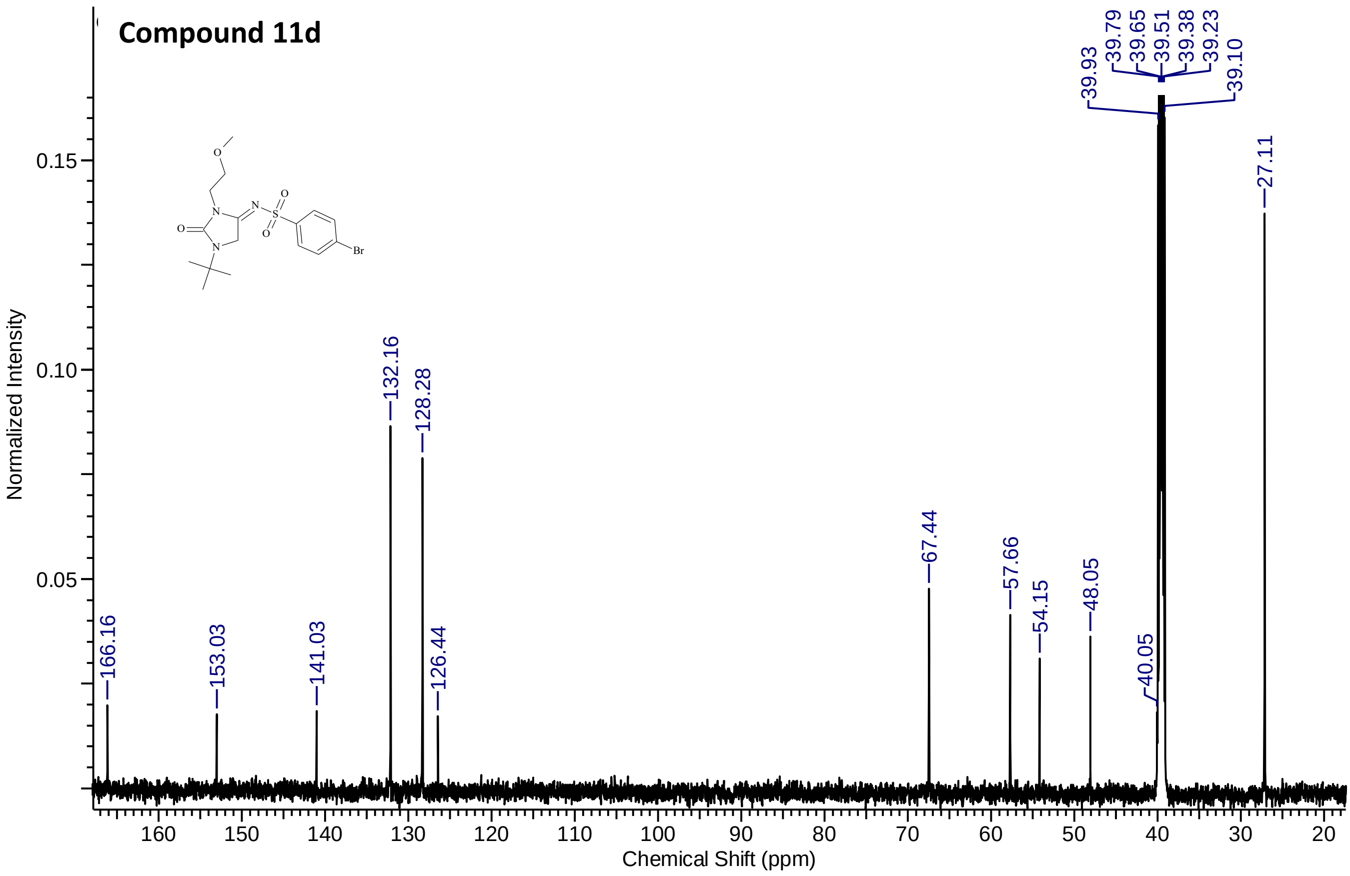

Compound 11d
39.93
39.79
39.65
39.51
39.38
39.23
39.10
27.11
132.16
128.28
67.44
57.66
54.15
48.05
40.05
166.16
153.03
141.03
126.44
Normalized Intensity
Chemical Shift (ppm)

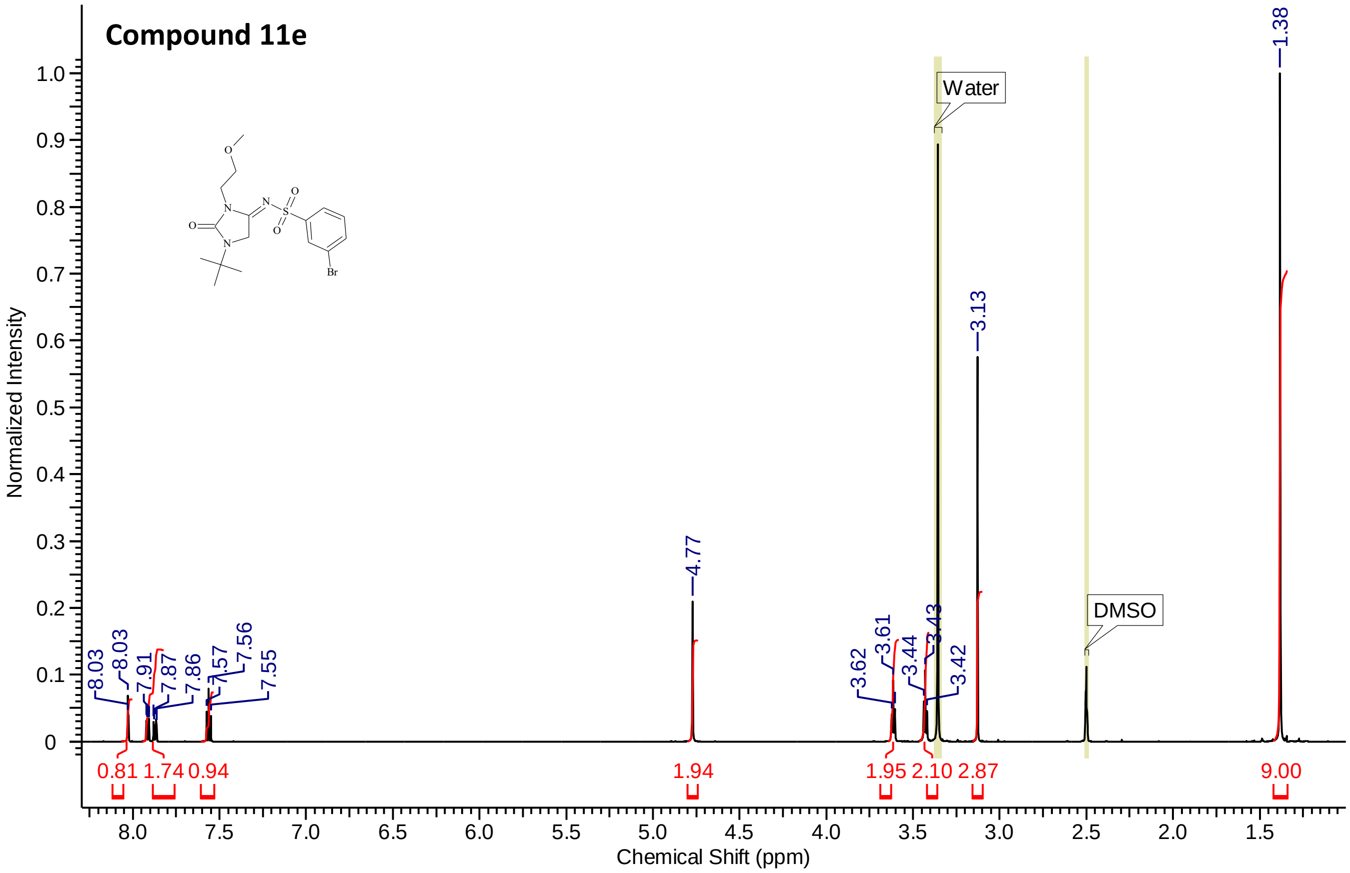
Compound 11e
Normalized Intensity
Chemical Shift (ppm)
Water
DMSO
8.03
8.03
7.91
7.87
7.86
7.57
7.56
7.55
4.77
3.62
3.61
3.44
3.43
3.42
3.13
1.38
0.81
1.74
0.94
1.94
1.95
2.10
2.87
9.00

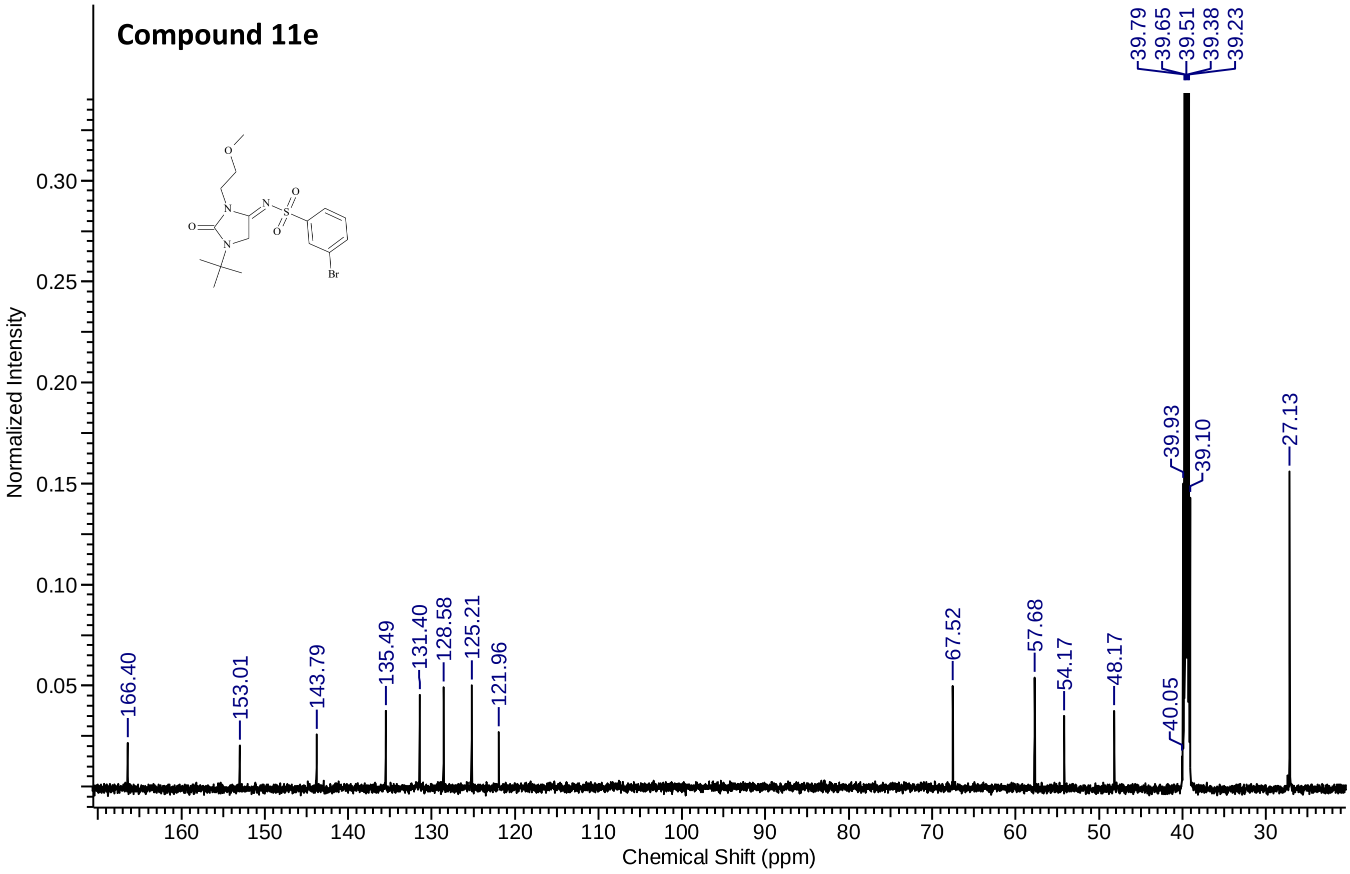
Compound 11e
Normalized Intensity
Chemical Shift (ppm)
166.40
153.01
143.79
135.49
131.40
128.58
125.21
121.96
67.52
57.68
54.17
48.17
40.05
39.93
39.79
39.65
39.51
39.38
39.23
39.10
27.13

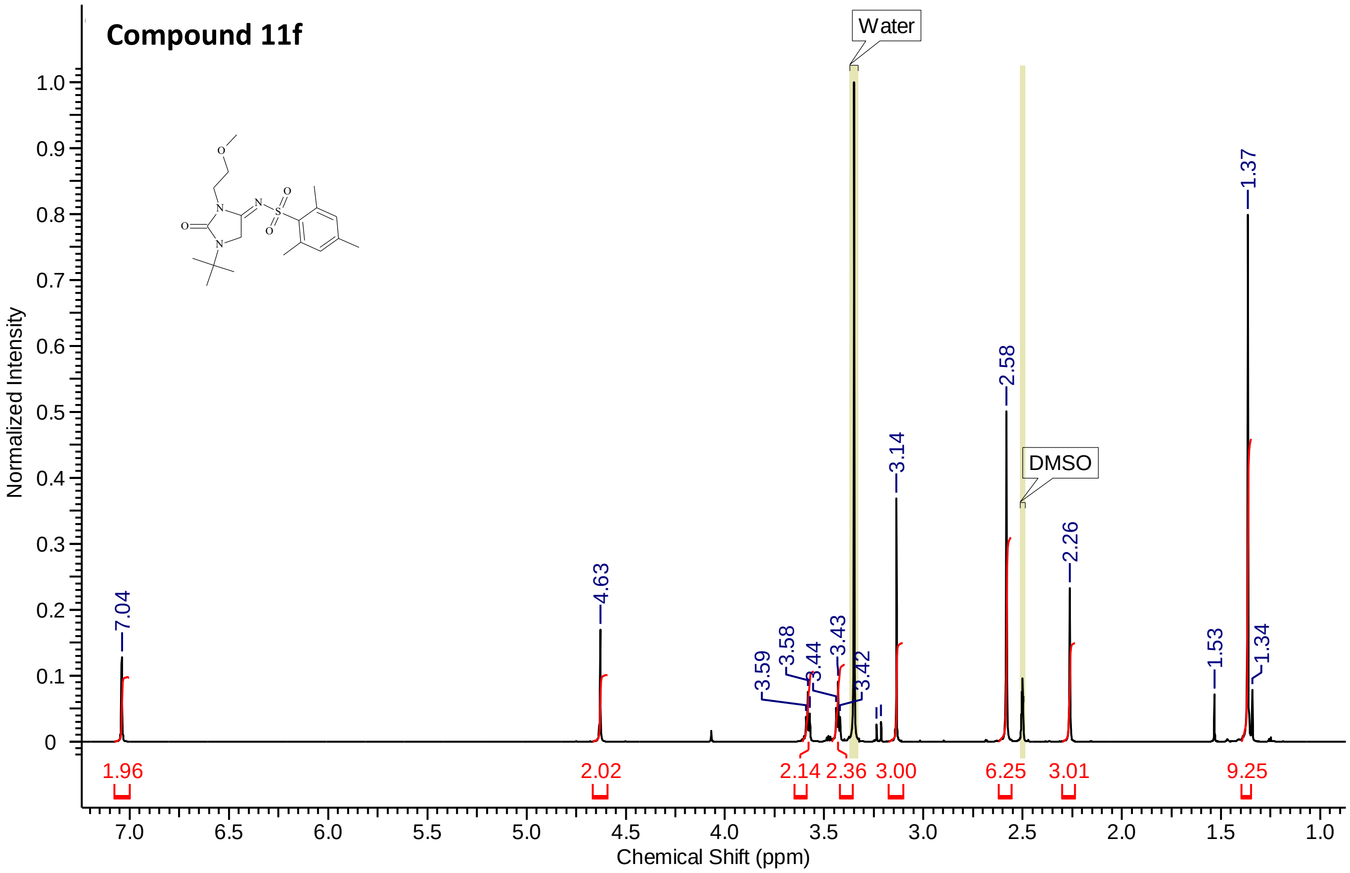
Compound 11f
Water
DMSO
Normalized Intensity
Chemical Shift (ppm)
7.04
4.63
3.59
3.58
3.44
3.43
3.42
3.14
2.58
2.26
1.53
1.37
1.34
1.96
2.02
2.14
2.36
3.00
6.25
3.01
9.25

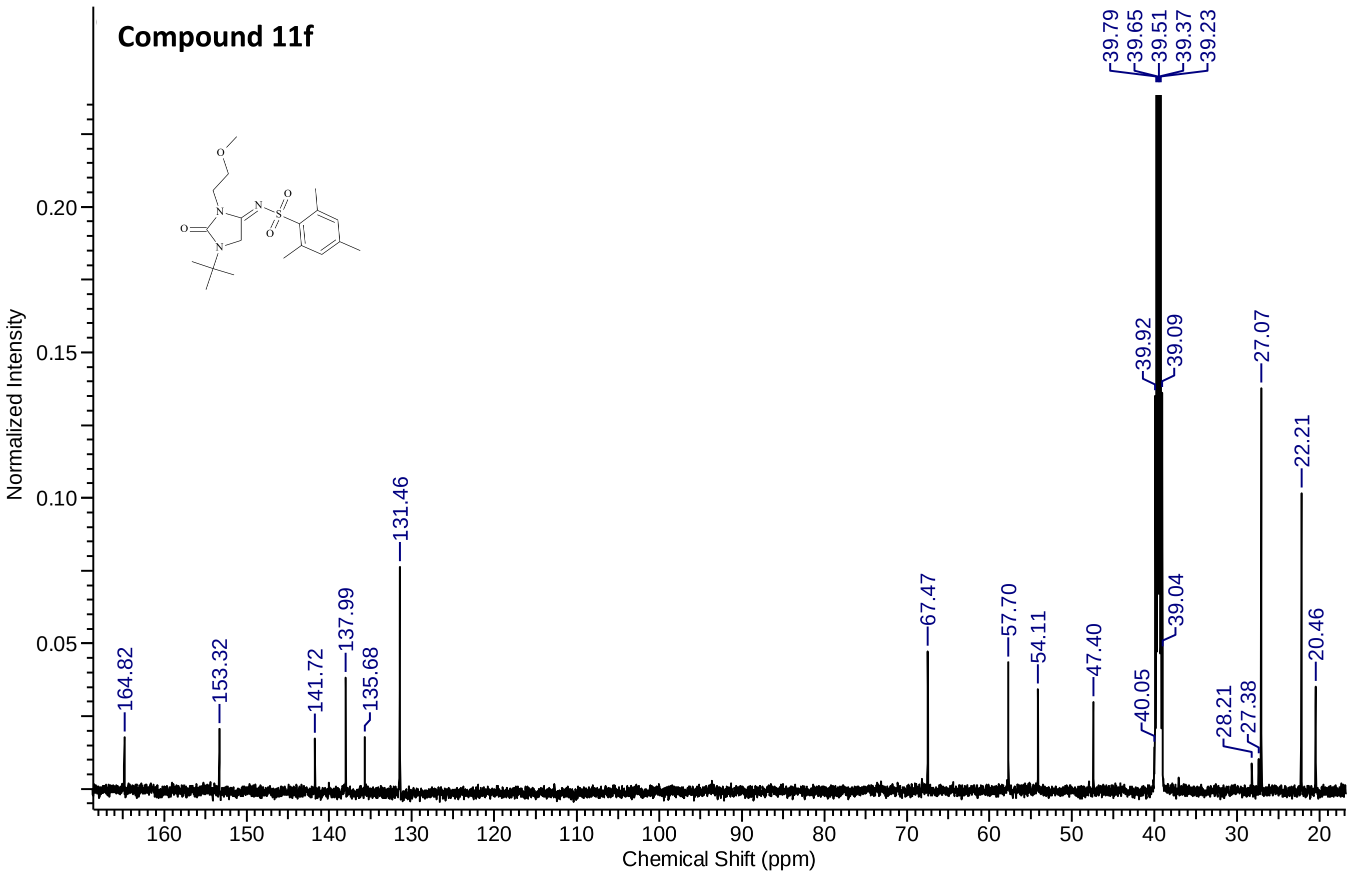
Compound 11f
Normalized Intensity
Chemical Shift (ppm)
164.82
153.32
141.72
137.99
135.68
131.46
67.47
57.70
54.11
47.40
40.05
39.92
39.79
39.65
39.51
39.37
39.23
39.09
39.04
28.21
27.38
27.07
22.21
20.46

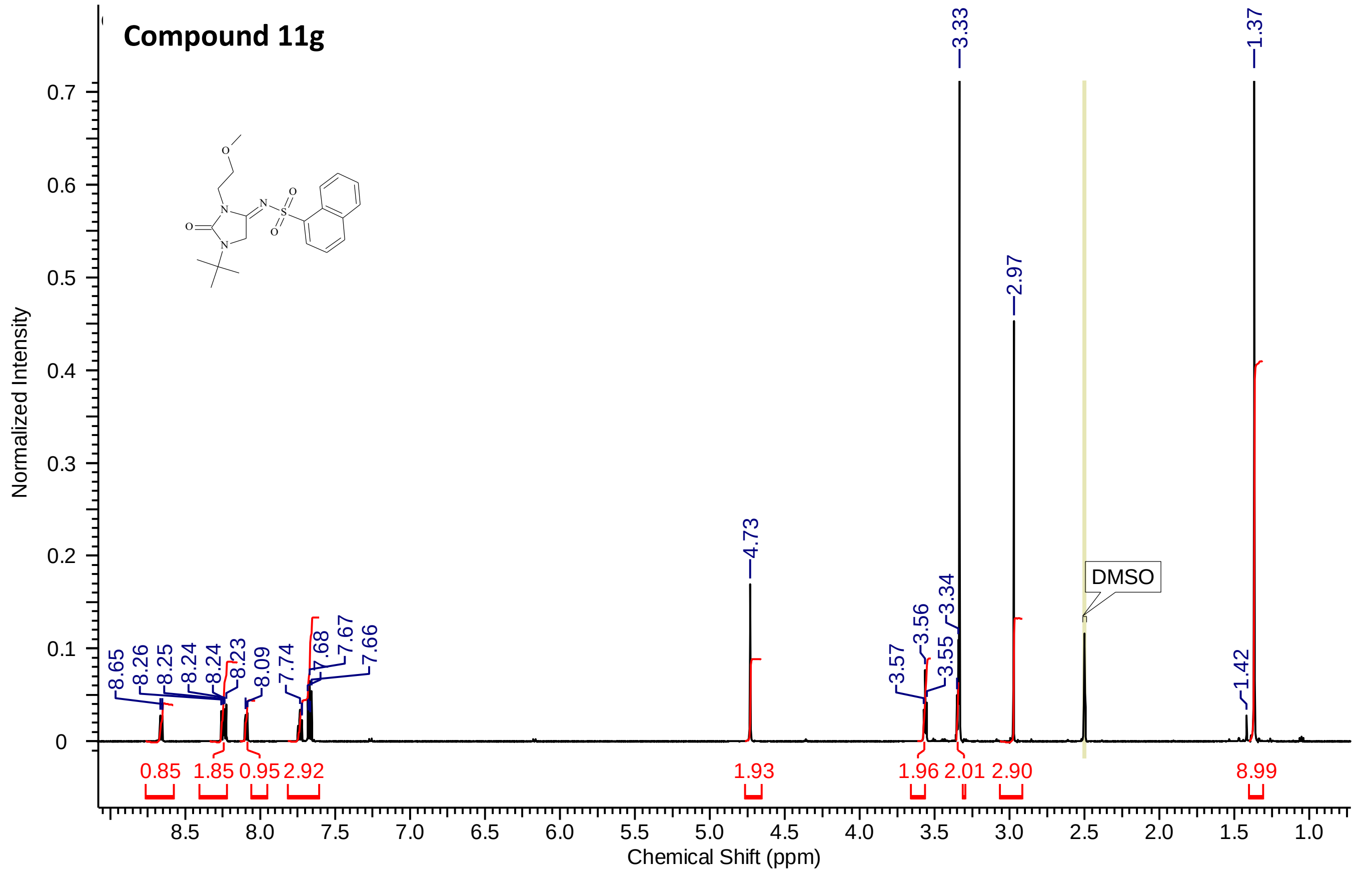
Compound 11g
Normalized Intensity
Chemical Shift (ppm)
DMSO
8.65
8.26
8.25
8.24
8.24
8.23
8.09
7.74
7.68
7.67
7.66
4.73
3.57
3.56
3.55
3.34
3.33
2.97
1.42
1.37
0.85
1.85
0.95
2.92
1.93
1.96
2.01
2.90
8.99

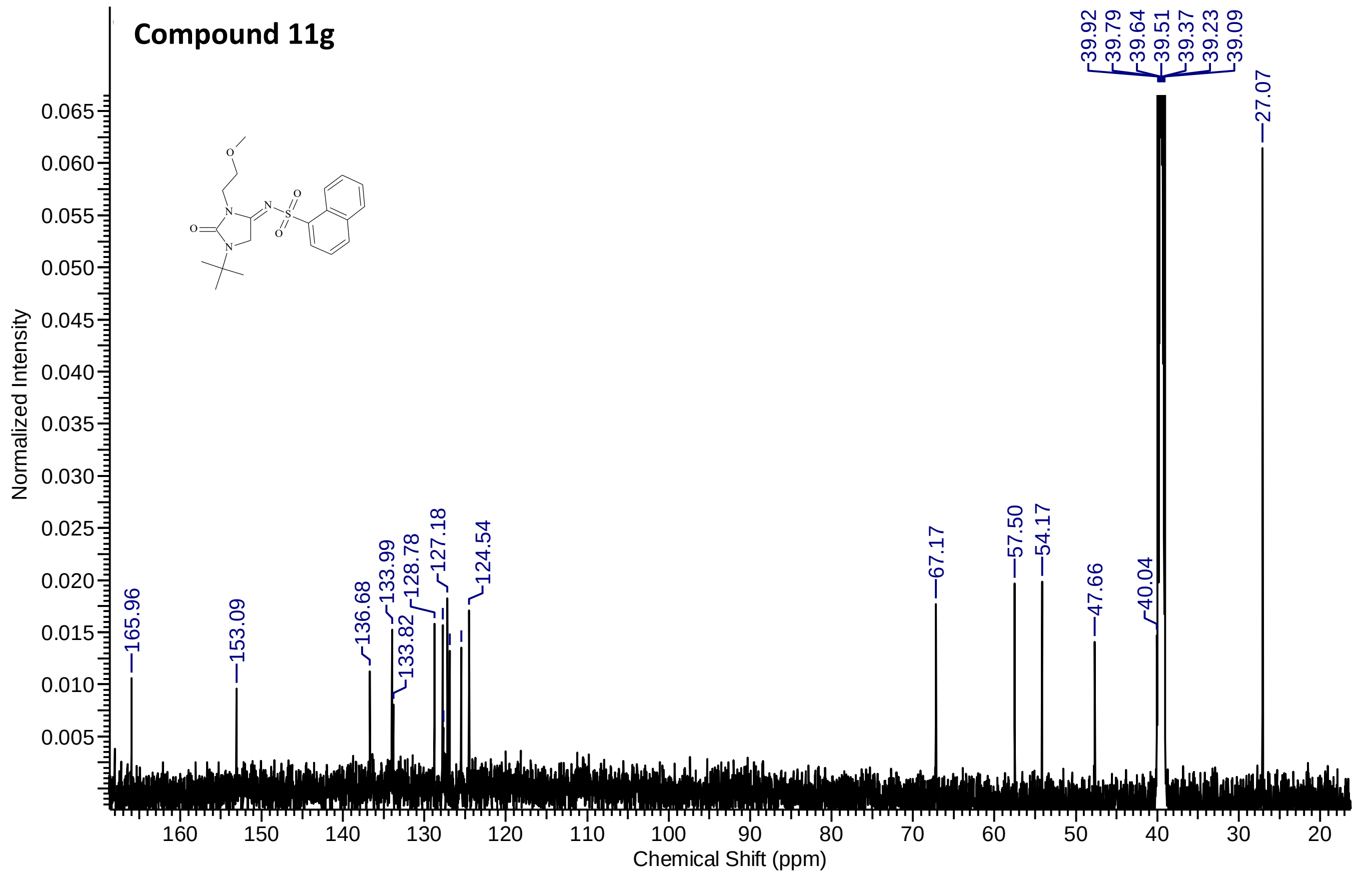
Compound 11g
Normalized Intensity
Chemical Shift (ppm)
165.96
153.09
136.68
133.99
133.82
128.78
127.18
124.54
67.17
57.50
54.17
47.66
40.04
39.92
39.79
39.64
39.51
39.37
39.23
39.09
27.07

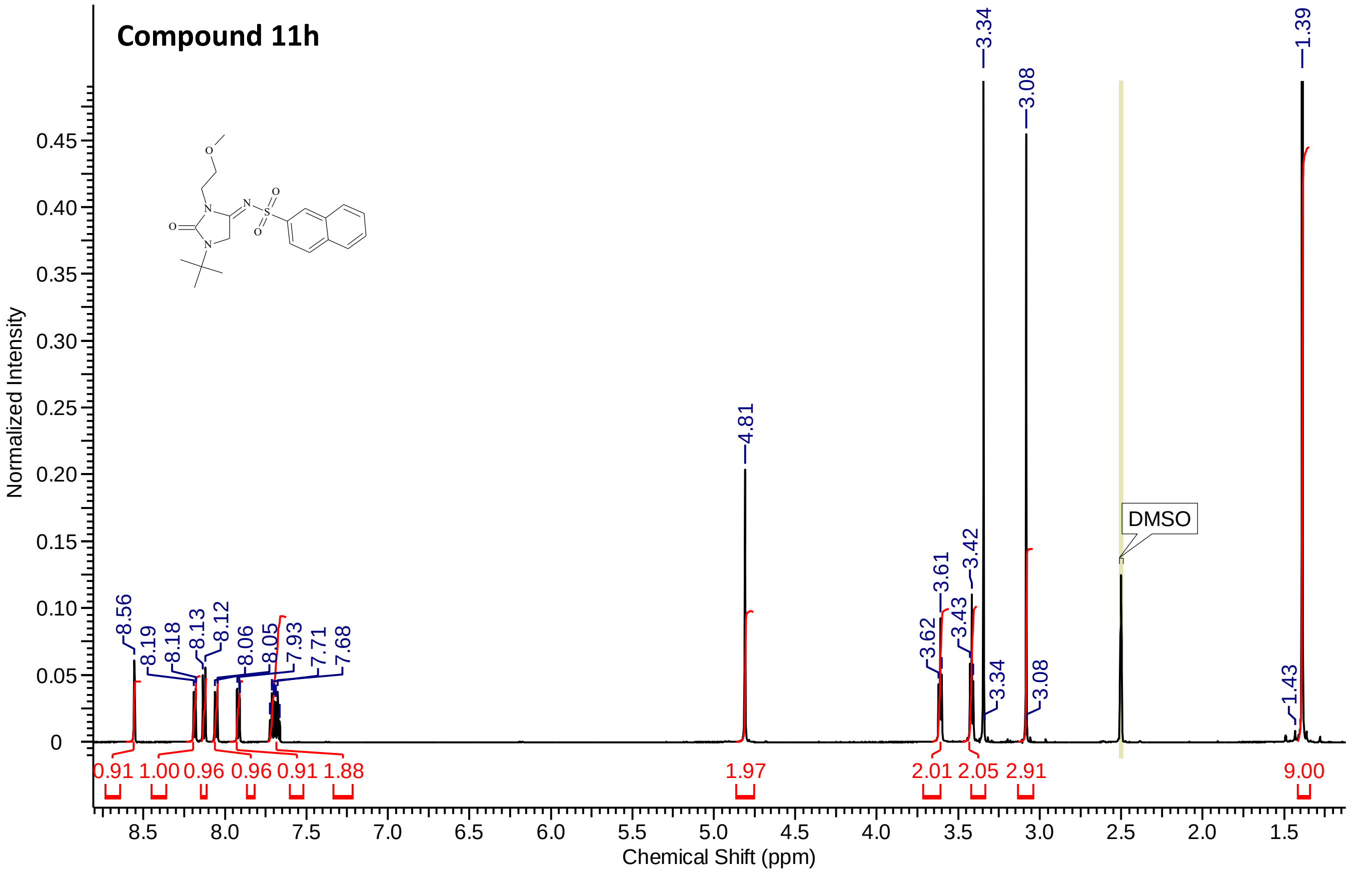

Compound 11h
8.56
8.19
8.18
8.13
8.12
8.06
8.05
7.93
7.71
7.68
4.81
3.62
3.61
3.43
3.42
3.34
3.34
3.08
3.08
DMSO
1.43
1.39
0.91 1.00 0.96 0.96 0.91 1.88
1.97
2.01 2.05 2.91
9.00
Normalized Intensity
Chemical Shift (ppm)

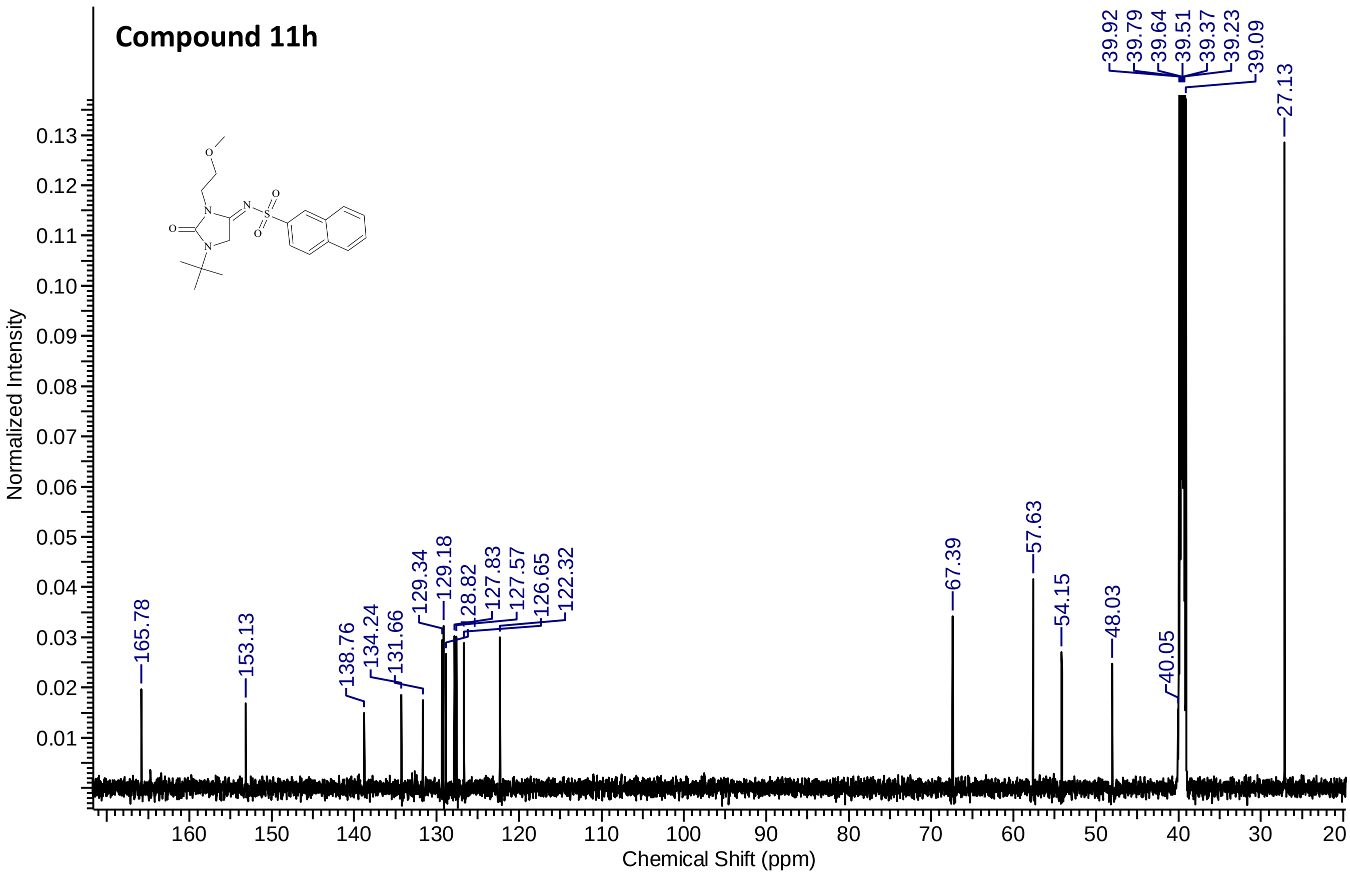

Compound 11h
165.78
153.13
138.76
134.24
131.66
129.34
129.18
128.82
127.83
127.57
126.65
122.32
67.39
57.63
54.15
48.03
40.05
39.92
39.79
39.64
39.51
39.37
39.23
39.09
27.13
Normalized Intensity
Chemical Shift (ppm)

Compound 12a

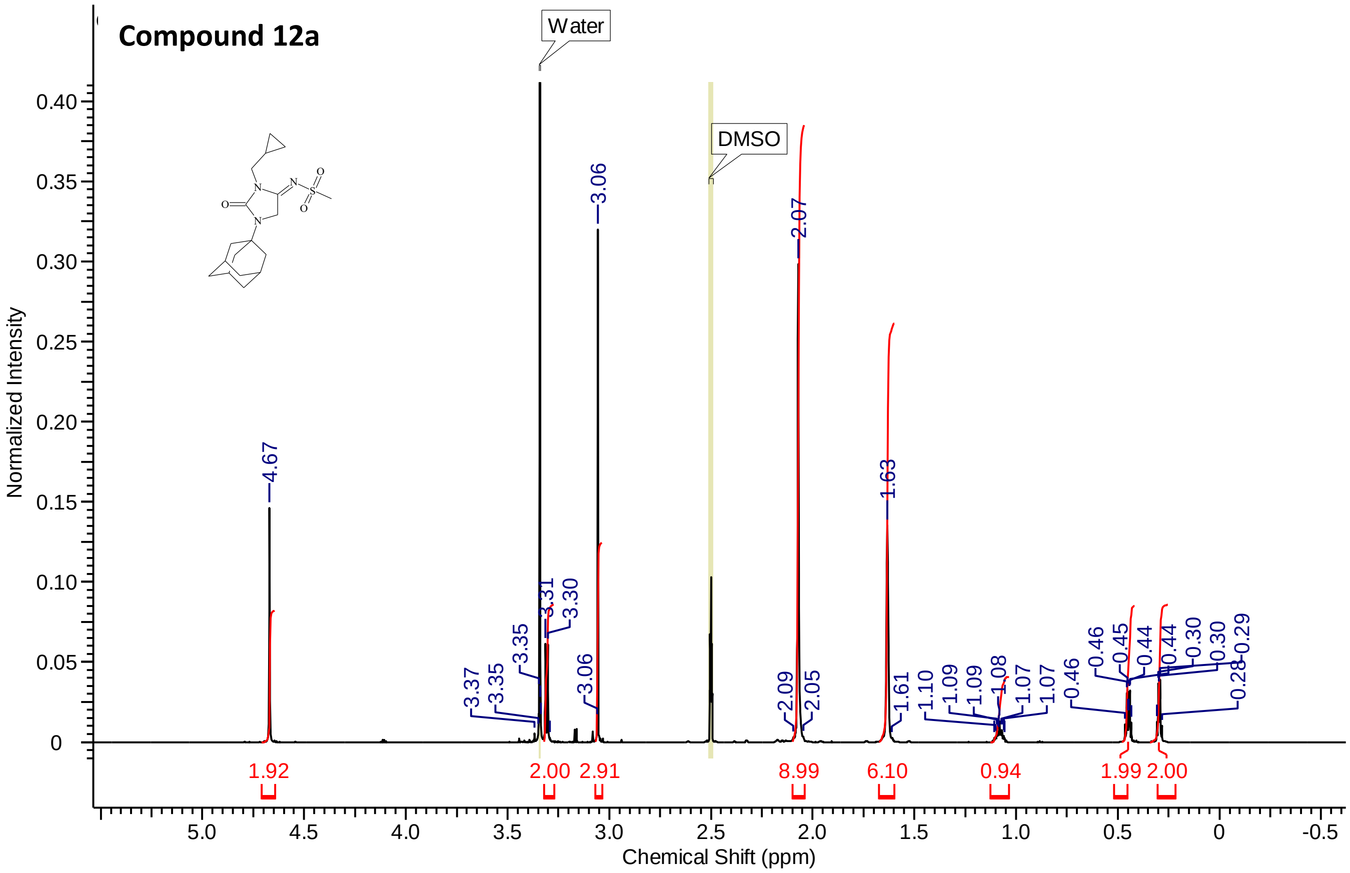

Compound 12a

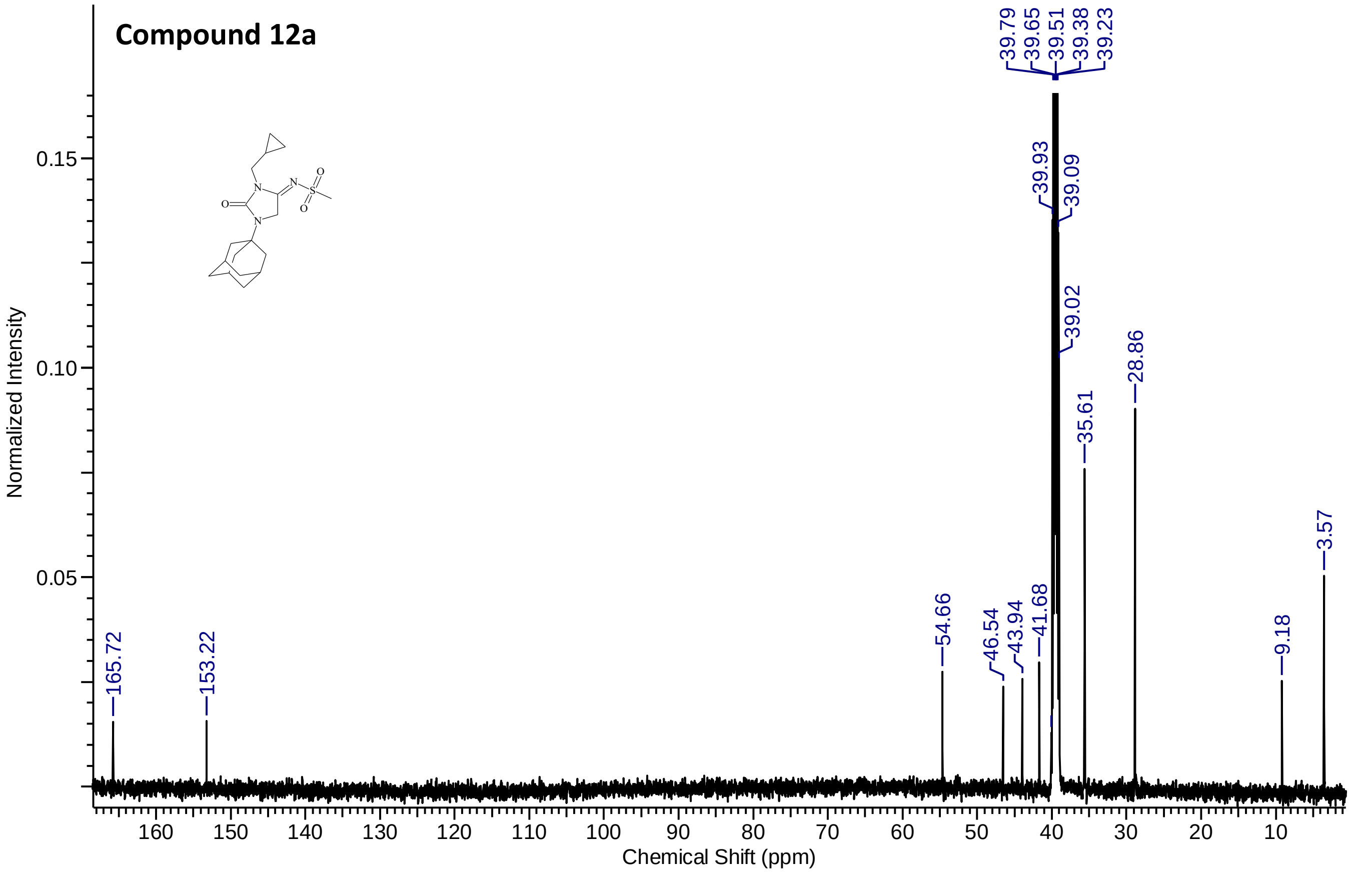

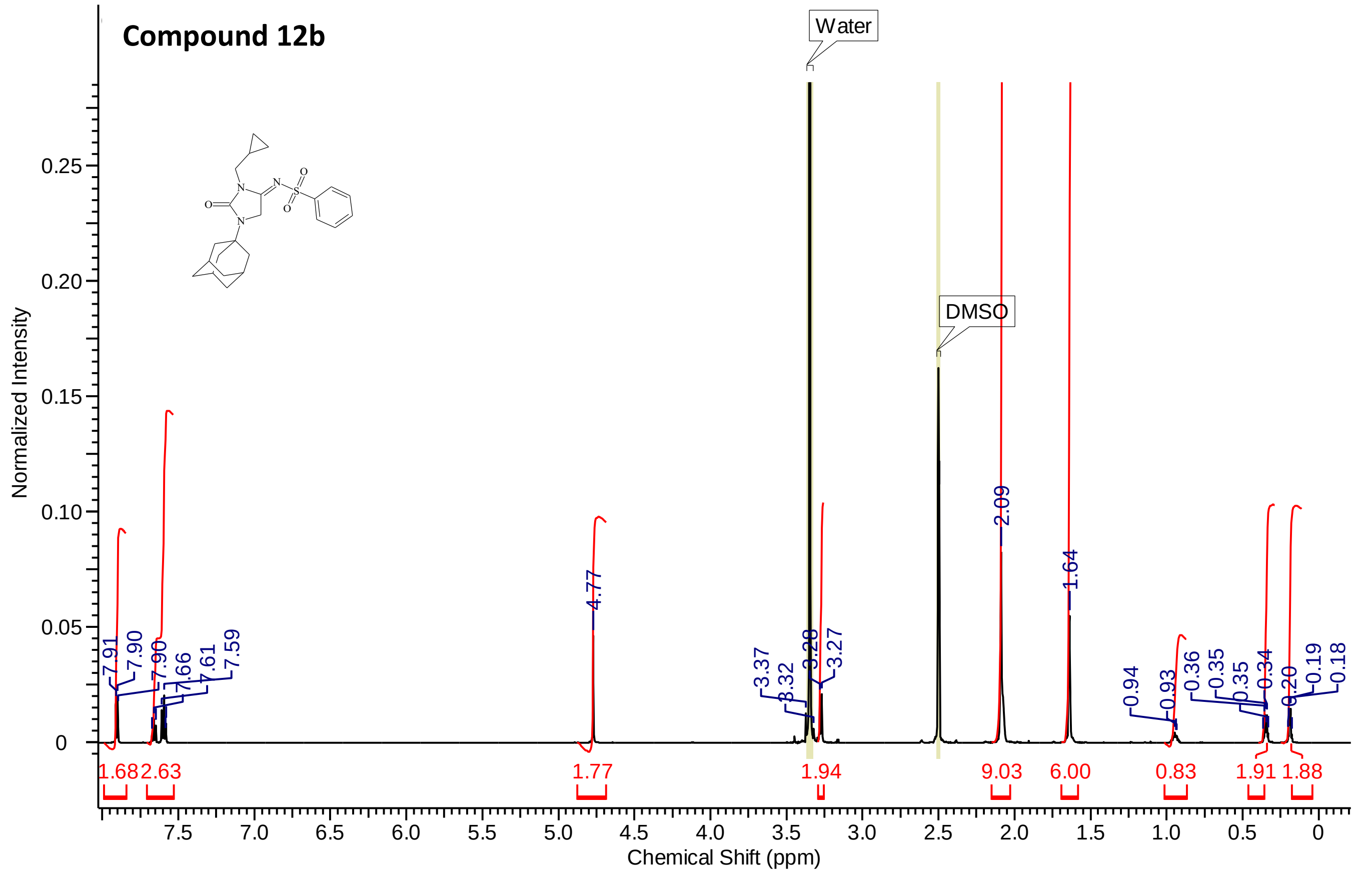
Compound 12b
Water
DMSO
Normalized Intensity
Chemical Shift (ppm)
7.91 7.90 7.90 7.66 7.61 7.59 4.77 3.37 3.32 3.28 3.27 2.09 1.64 0.94 0.93 0.36 0.35 0.35 0.34 0.20 0.19 0.18
1.68 2.63 1.77 1.94 9.03 6.00 0.83 1.91 1.88

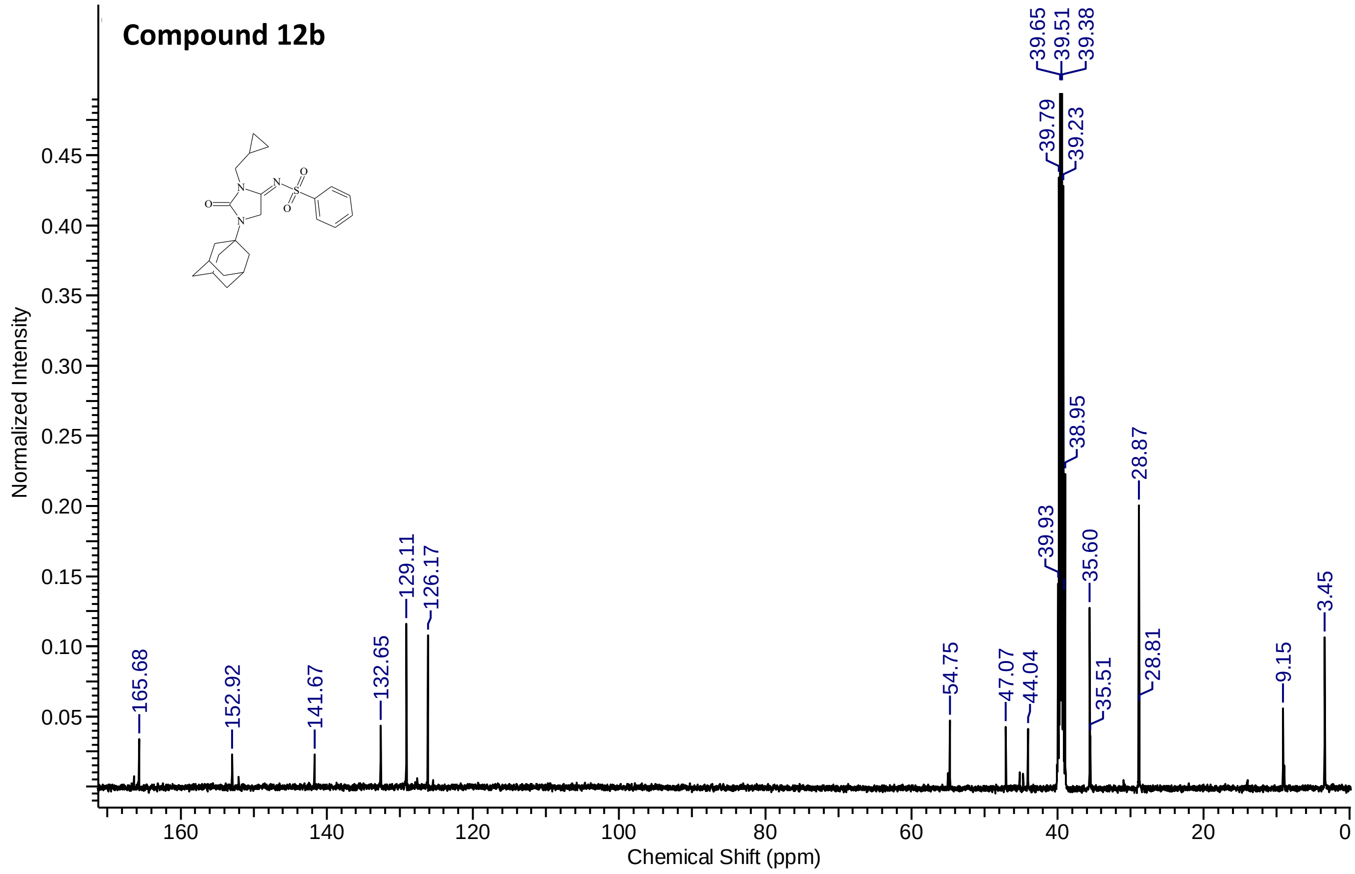
Compound 12b
Normalized Intensity
Chemical Shift (ppm)
165.68 152.92 141.67 132.65 129.11 126.17 54.75 47.07 44.04 39.93 39.79 39.65 39.51 39.38 39.23 38.95 35.60 35.51 28.87 28.81 9.15 3.45

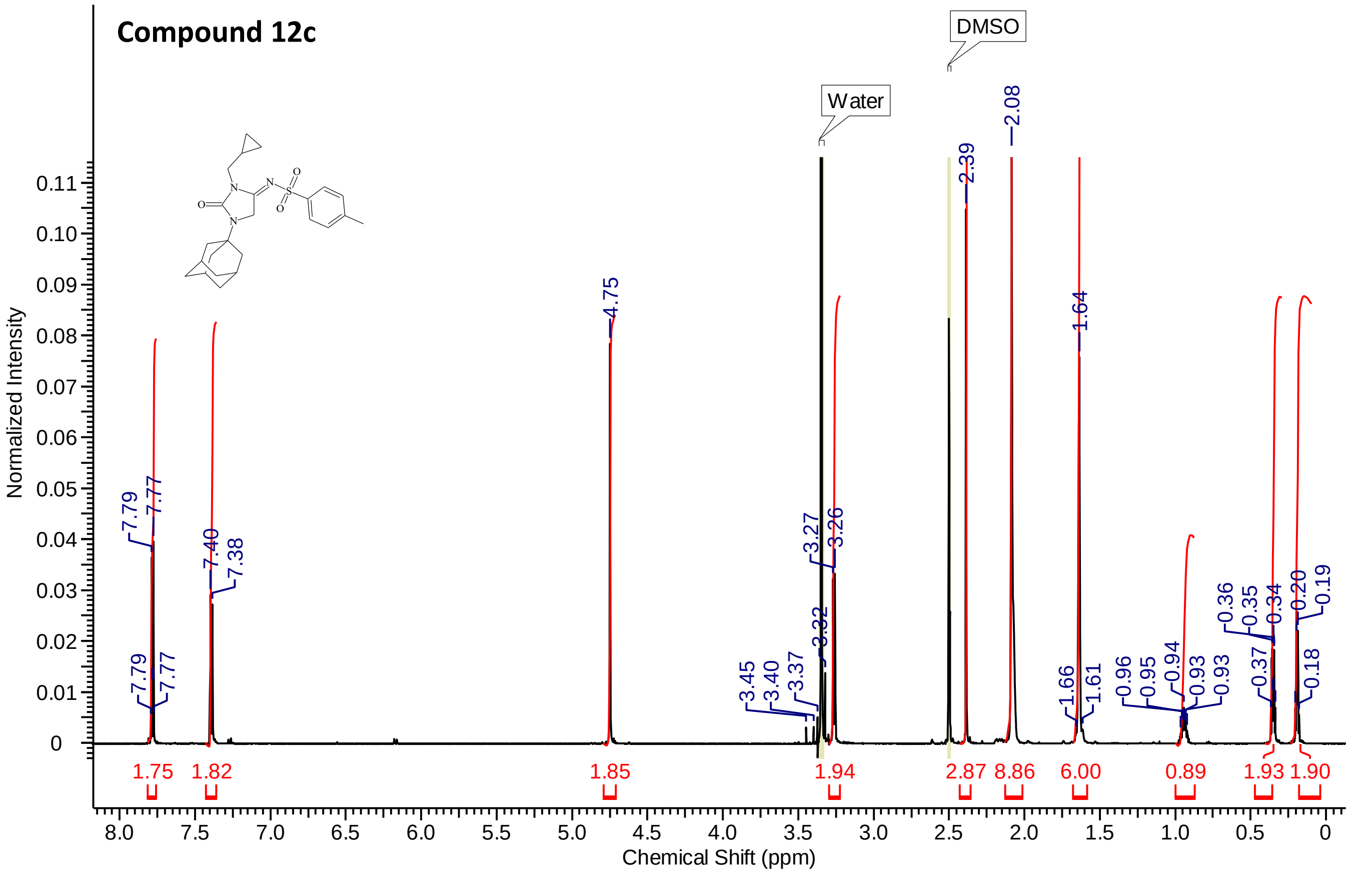
Compound 12c
Water
DMSO
Normalized Intensity
Chemical Shift (ppm)
7.79 7.77 7.79 7.77 7.40 7.38 4.75 3.45 3.40 3.37 3.32 3.27 3.26 2.39 2.08 1.66 1.64 1.61 0.96 0.95 0.94 0.93 0.93 0.37 0.36 0.35 0.34 0.20 0.19 0.18
1.75 1.82 1.85 1.94 2.87 8.86 6.00 0.89 1.93 1.90

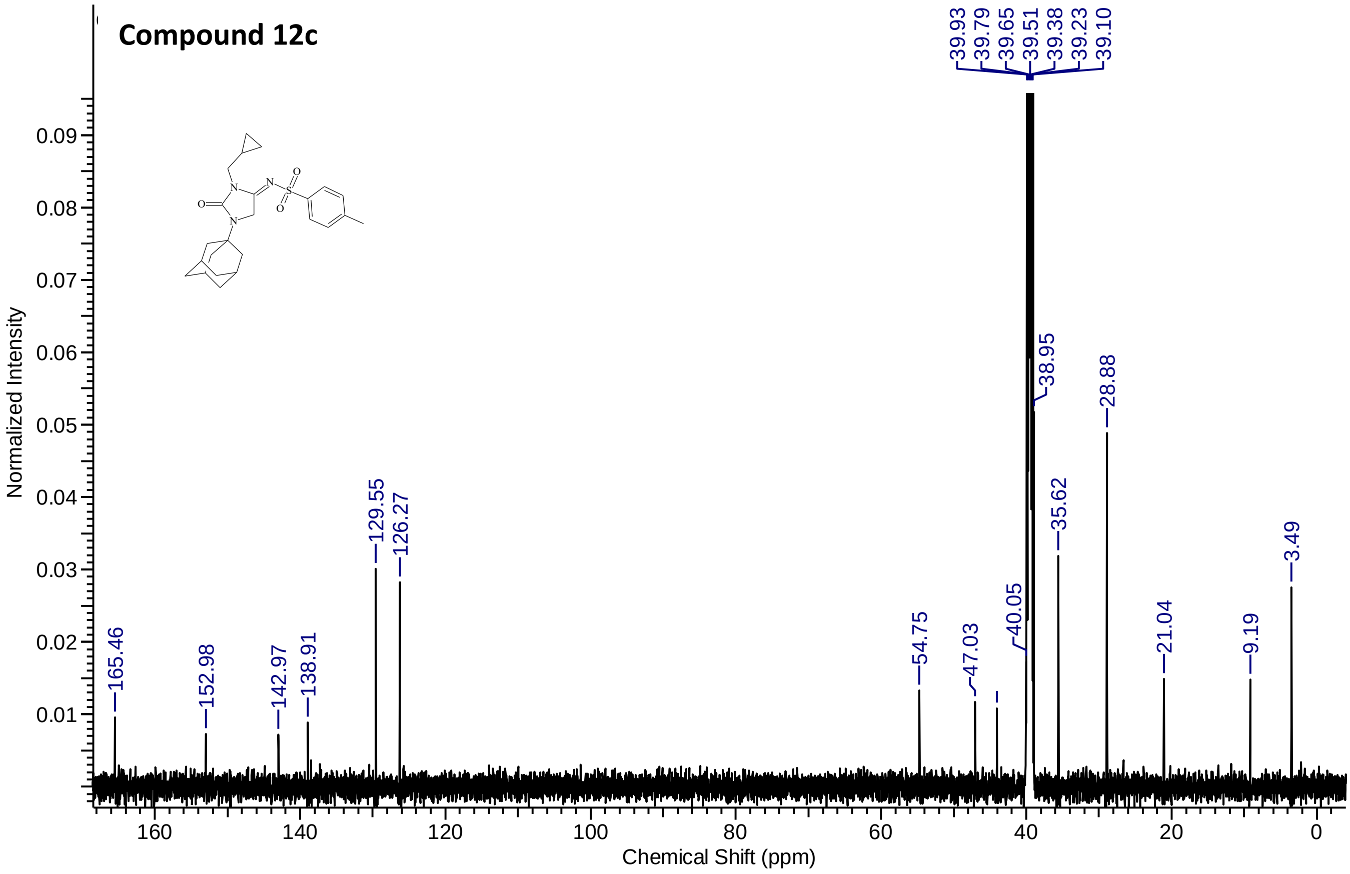
Compound 12c
Normalized Intensity
Chemical Shift (ppm)
39.93 39.79 39.65 39.51 39.38 39.23 39.10
165.46 152.98 142.97 138.91 129.55 126.27 54.75 47.03 40.05 38.95 35.62 28.88 21.04 9.19 3.49

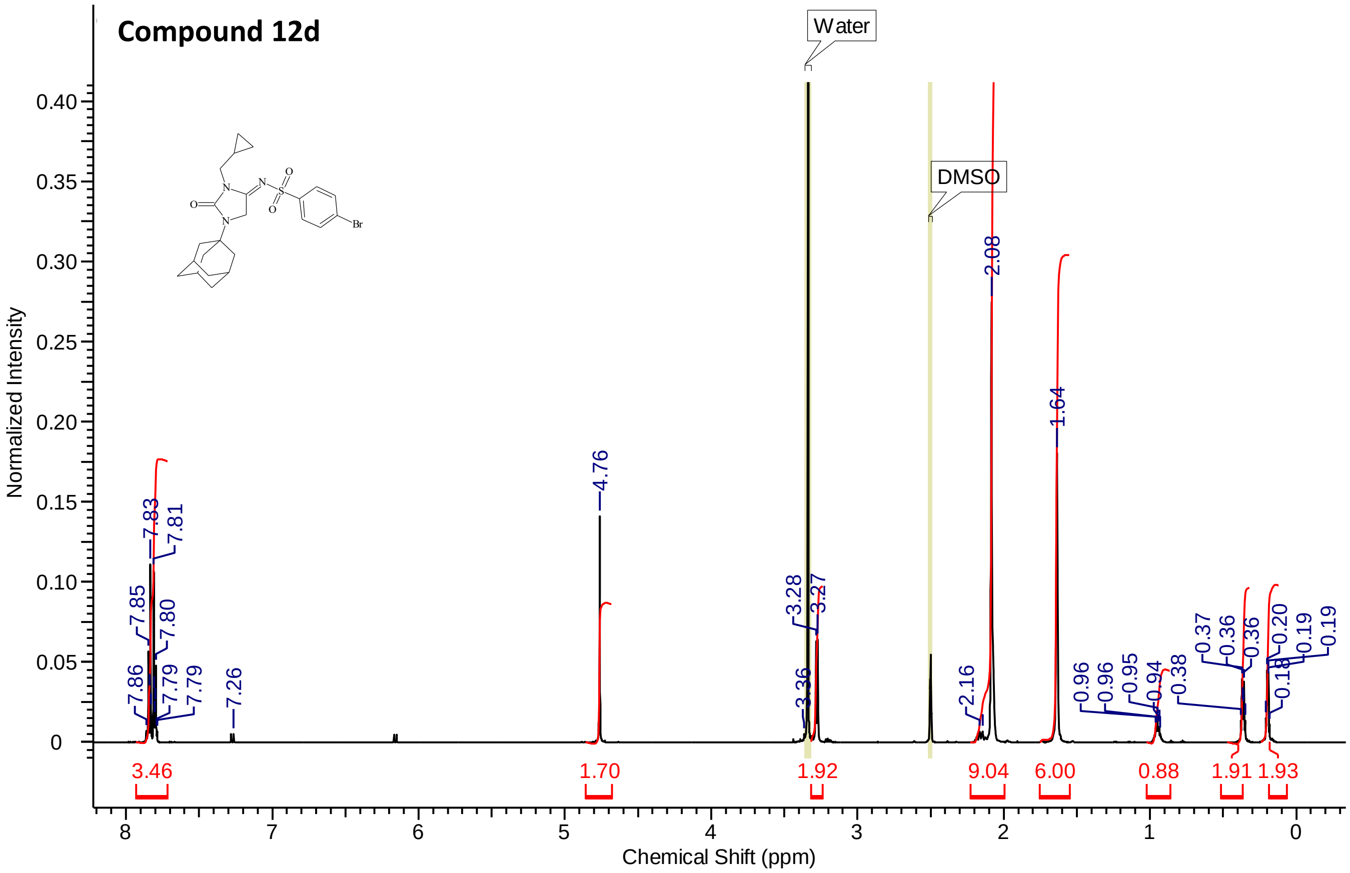
Compound 12d
Water
DMSO
Normalized Intensity
Chemical Shift (ppm)
7.86
7.85
7.83
7.81
7.80
7.79
7.79
7.26
4.76
3.36
3.28
3.27
2.16
2.08
1.64
0.96
0.96
0.95
0.94
0.38
0.37
0.36
0.36
0.20
0.19
0.19
0.18
3.46
1.70
1.92
9.04
6.00
0.88
1.91
1.93

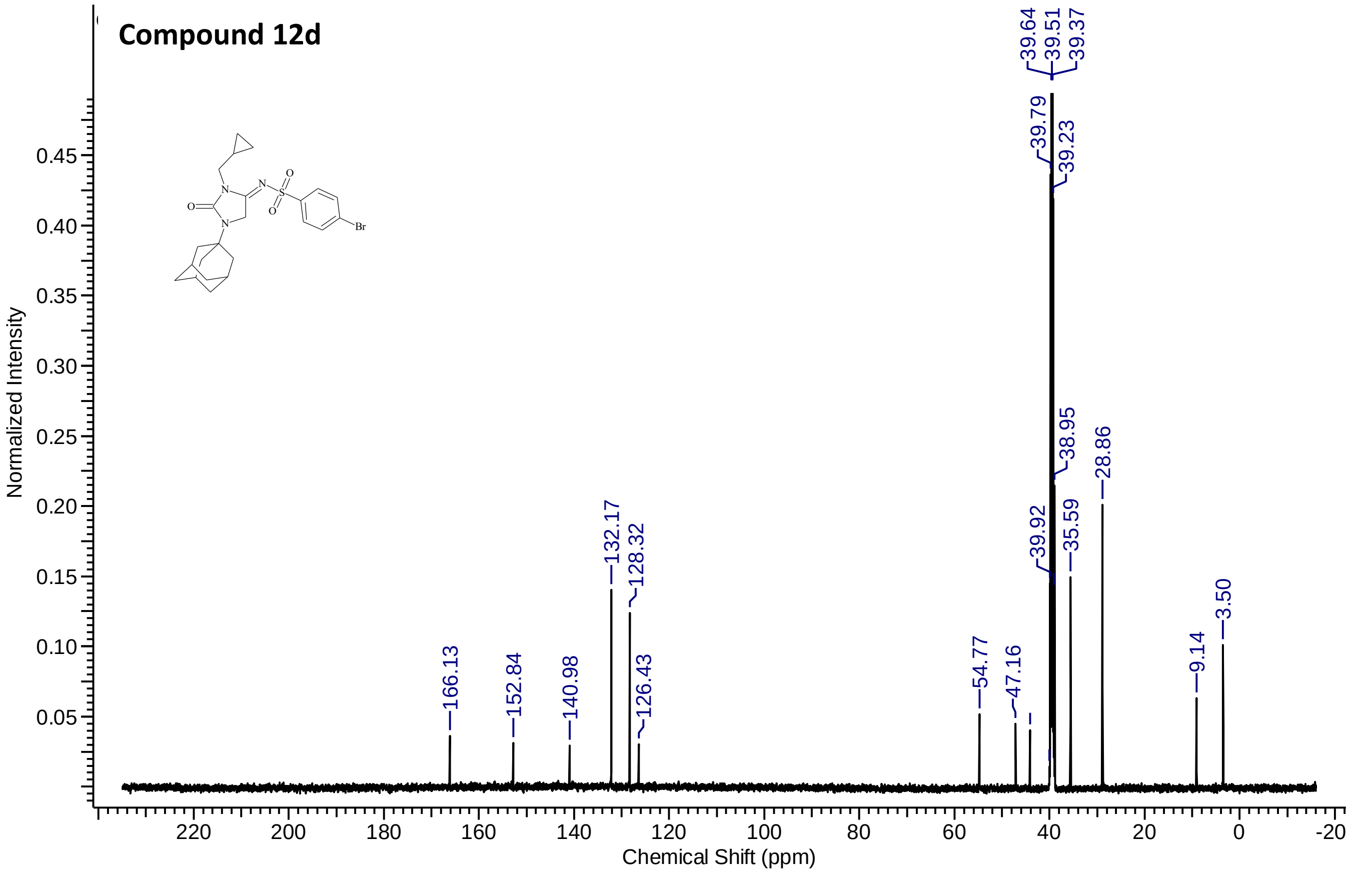
Compound 12d
Normalized Intensity
Chemical Shift (ppm)
166.13
152.84
140.98
132.17
128.32
126.43
54.77
47.16
39.92
39.79
39.64
39.51
39.37
39.23
38.95
35.59
28.86
9.14
3.50

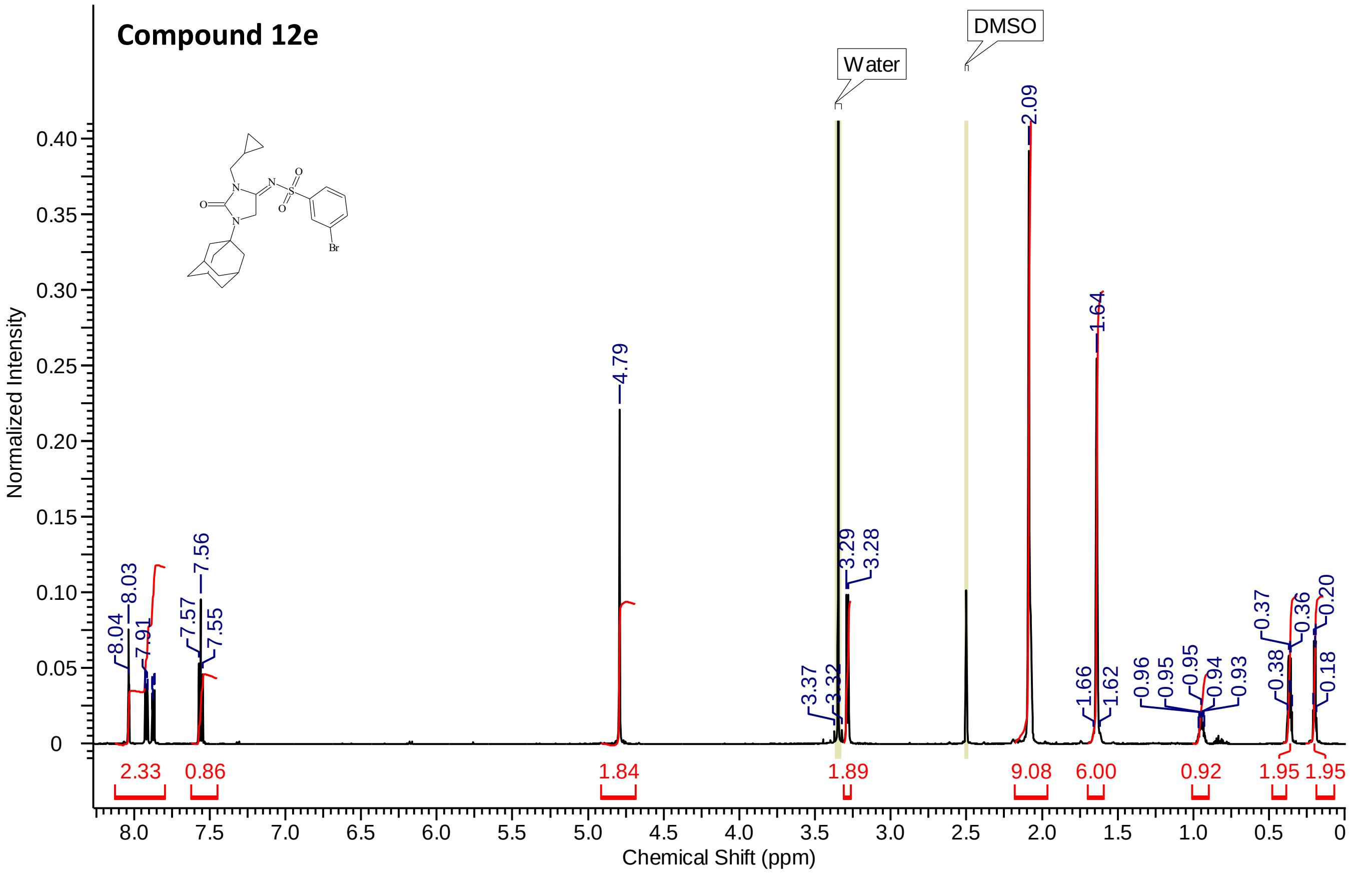
Compound 12e
Water
DMSO
Normalized Intensity
Chemical Shift (ppm)
8.04
8.03
7.91
7.57
7.56
7.55
4.79
3.37
3.32
3.29
3.28
2.09
1.66
1.64
1.62
0.96
0.95
0.95
0.94
0.93
0.38
0.37
0.36
0.20
0.18
2.33
0.86
1.84
1.89
9.08
6.00
0.92
1.95
1.95
Br

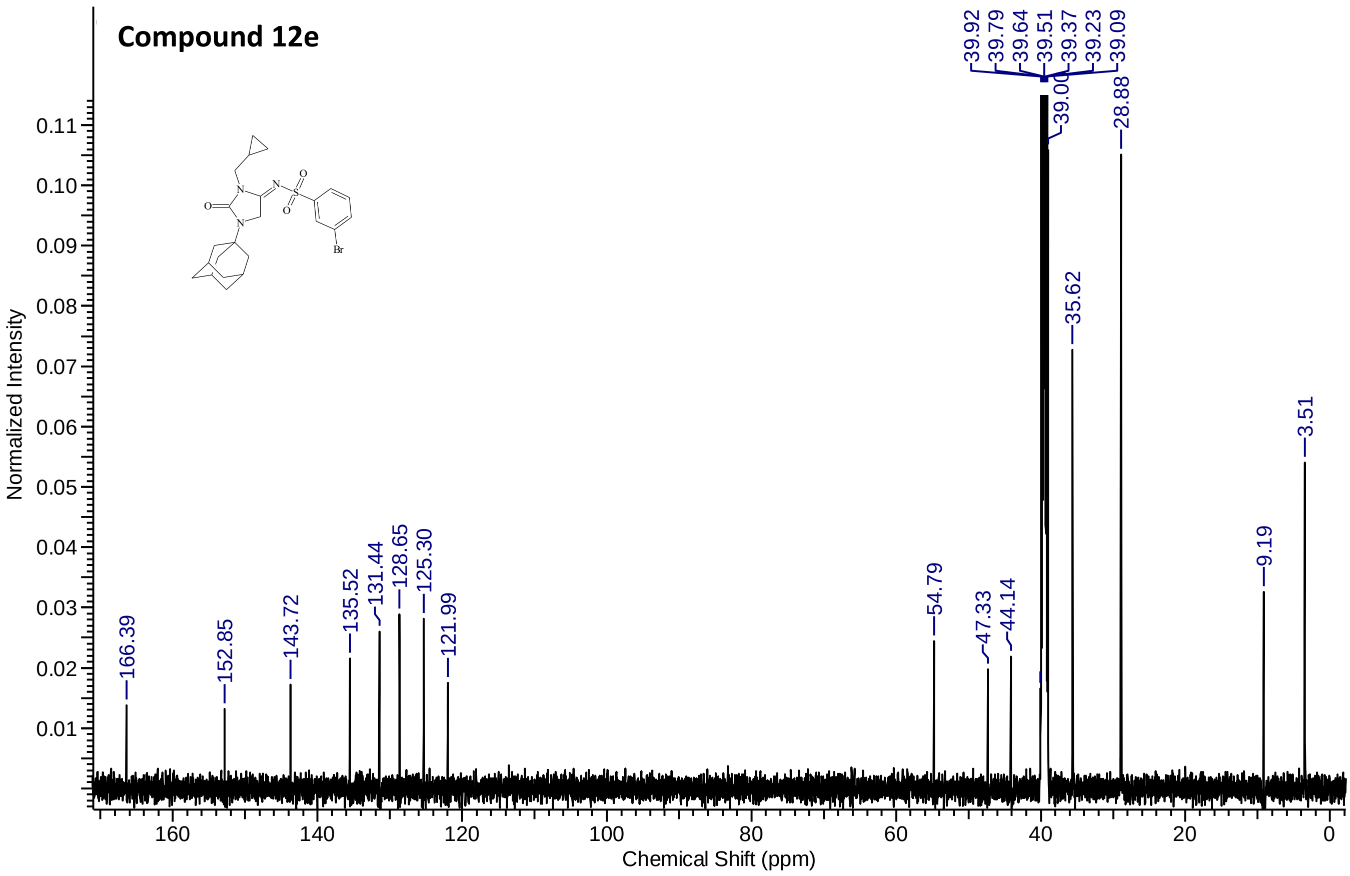
Compound 12e
Normalized Intensity
Chemical Shift (ppm)
166.39
152.85
143.72
135.52
131.44
128.65
125.30
121.99
54.79
47.33
44.14
39.92
39.79
39.64
39.51
39.37
39.23
39.09
39.00
35.62
28.88
9.19
3.51
Br

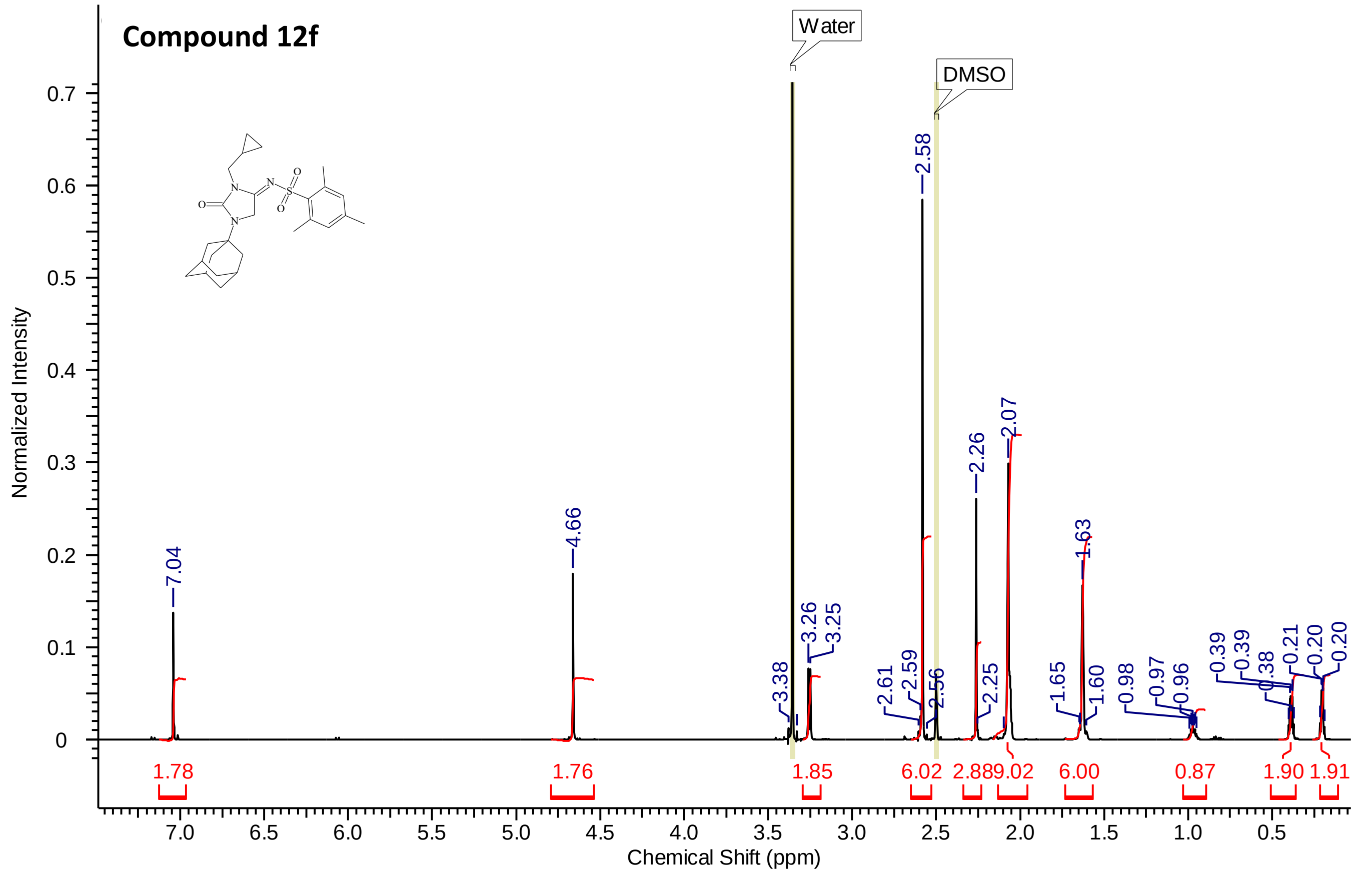

Compound 12f
Water
DMSO
Normalized Intensity
Chemical Shift (ppm)
7.04
4.66
3.38
3.26
3.25
2.61
2.59
2.58
2.56
2.26
2.25
2.07
1.65
1.63
1.60
0.98
0.97
0.96
0.39
0.39
0.38
0.21
0.20
0.20
1.78
1.76
1.85
6.02
2.88
9.02
6.00
0.87
1.90
1.91

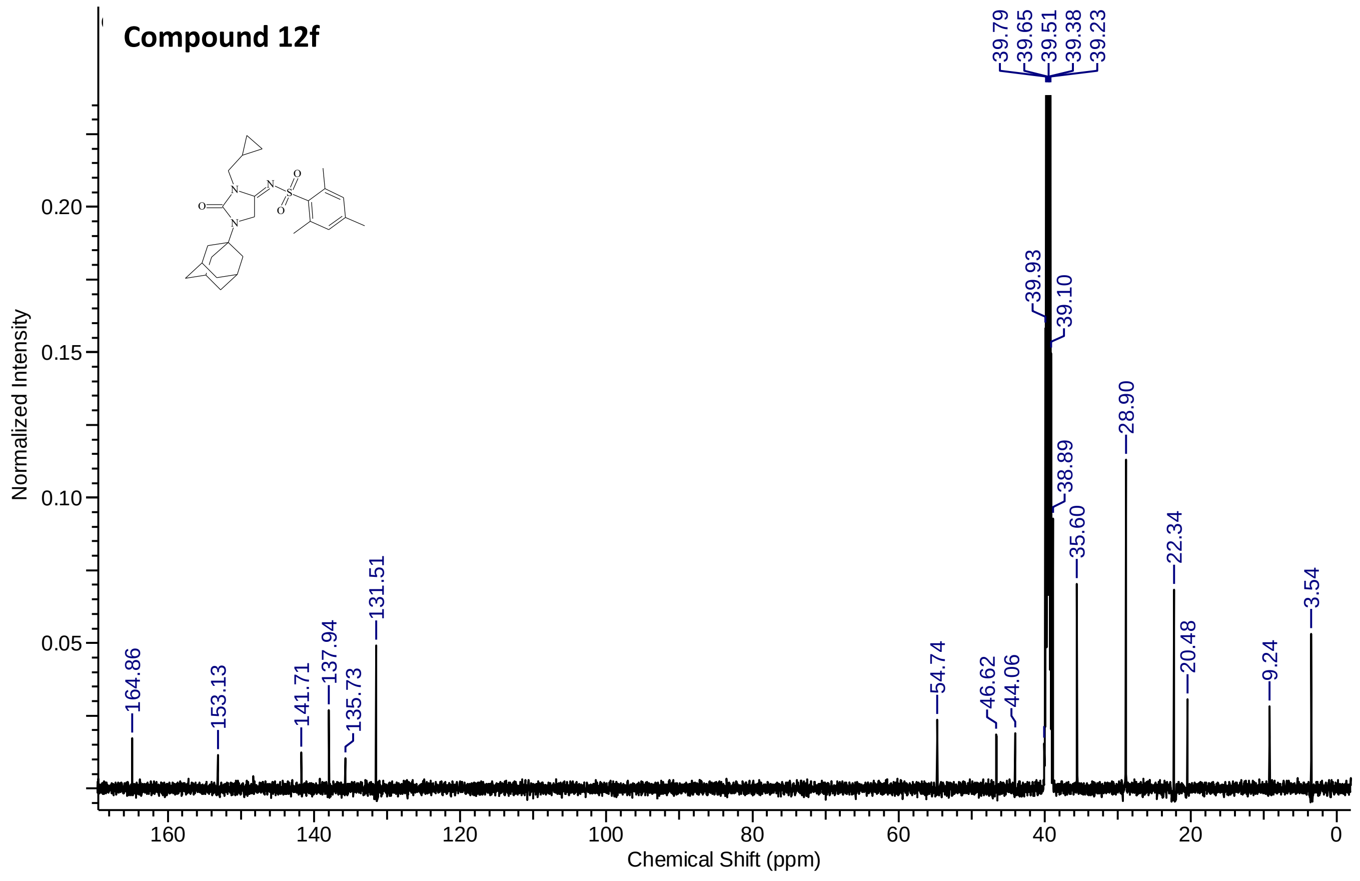

Compound 12f
Normalized Intensity
Chemical Shift (ppm)
164.86
153.13
141.71
137.94
135.73
131.51
54.74
46.62
44.06
39.93
39.79
39.65
39.51
39.38
39.23
39.10
38.89
35.60
28.90
22.34
20.48
9.24
3.54

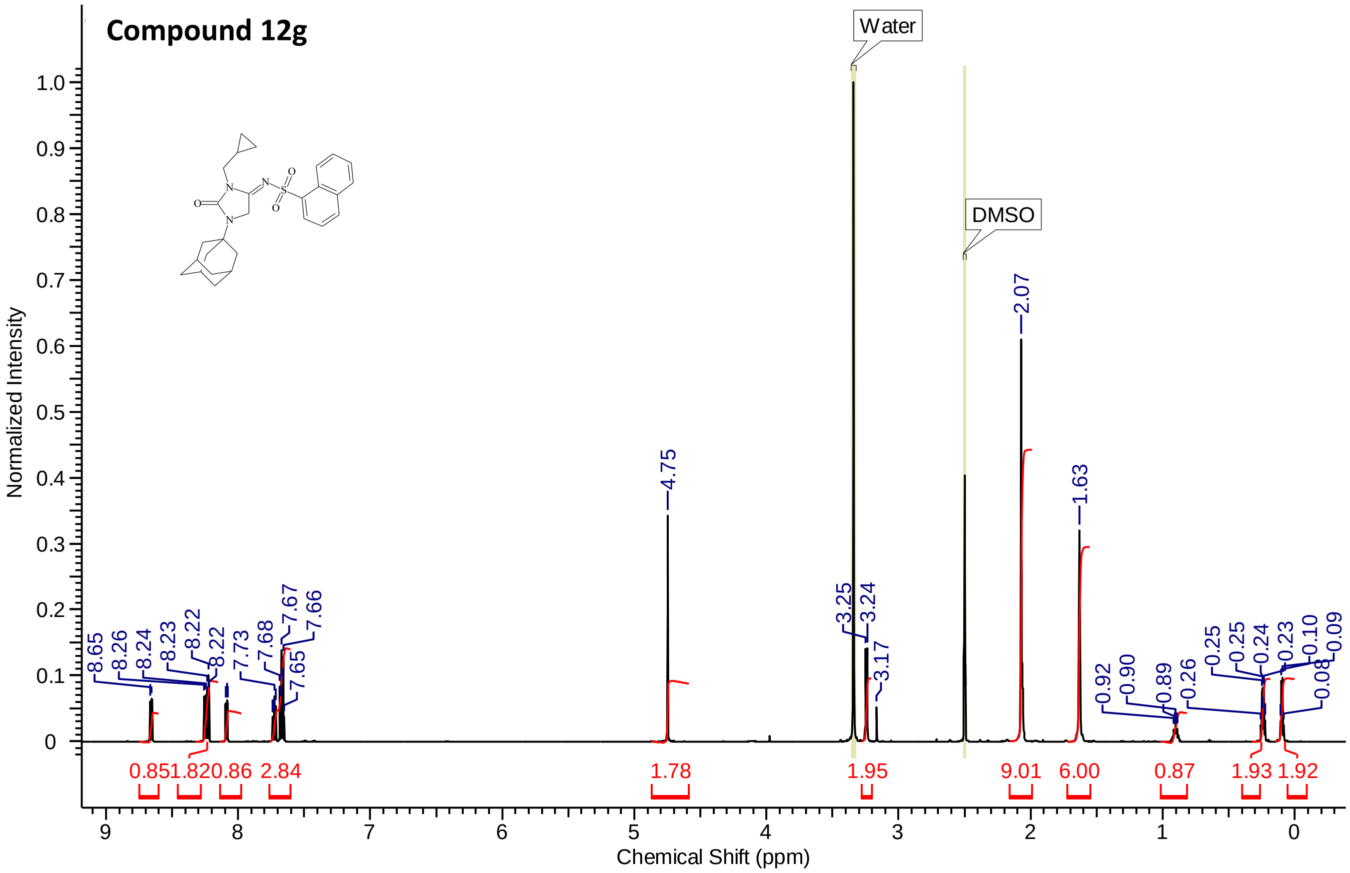
Compound 12g
Water
DMSO
Normalized Intensity
Chemical Shift (ppm)
8.65 8.26 8.24 8.23 8.22 8.22 7.73 7.68 7.67 7.66 7.65
4.75
3.25 3.24 3.17
2.07
1.63
0.92 0.90 0.89 0.26 0.25 0.25 0.24 0.23 0.10 0.09 0.08
0.85 1.82 0.86 2.84 1.78 1.95 9.01 6.00 0.87 1.93 1.92

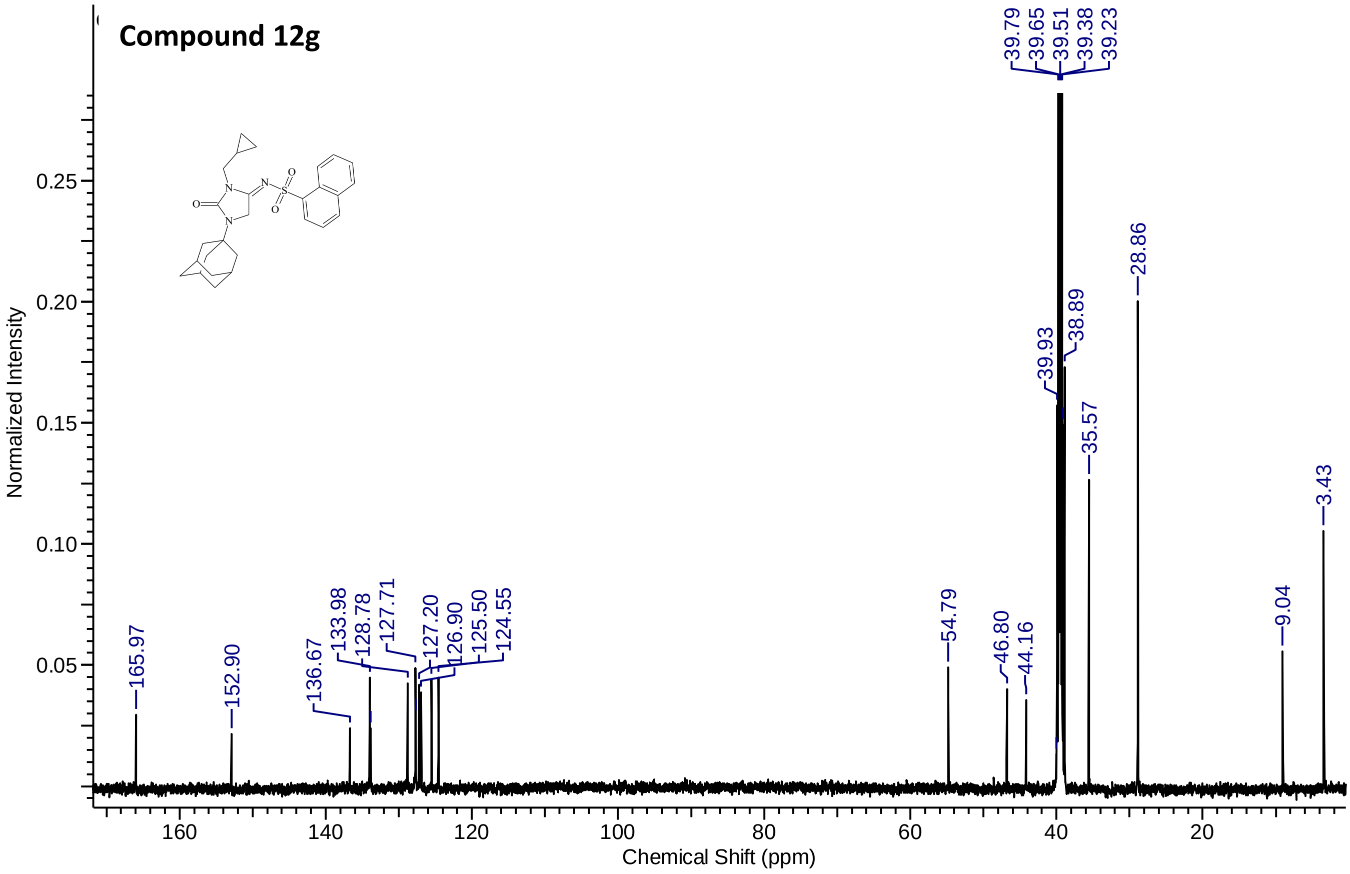
Compound 12g
Normalized Intensity
Chemical Shift (ppm)
165.97
152.90
136.67 133.98 128.78 127.71 127.20 126.90 125.50 124.55
54.79
46.80 44.16
39.93 39.79 39.65 39.51 39.38 39.23 38.89
35.57
28.86
9.04
3.43

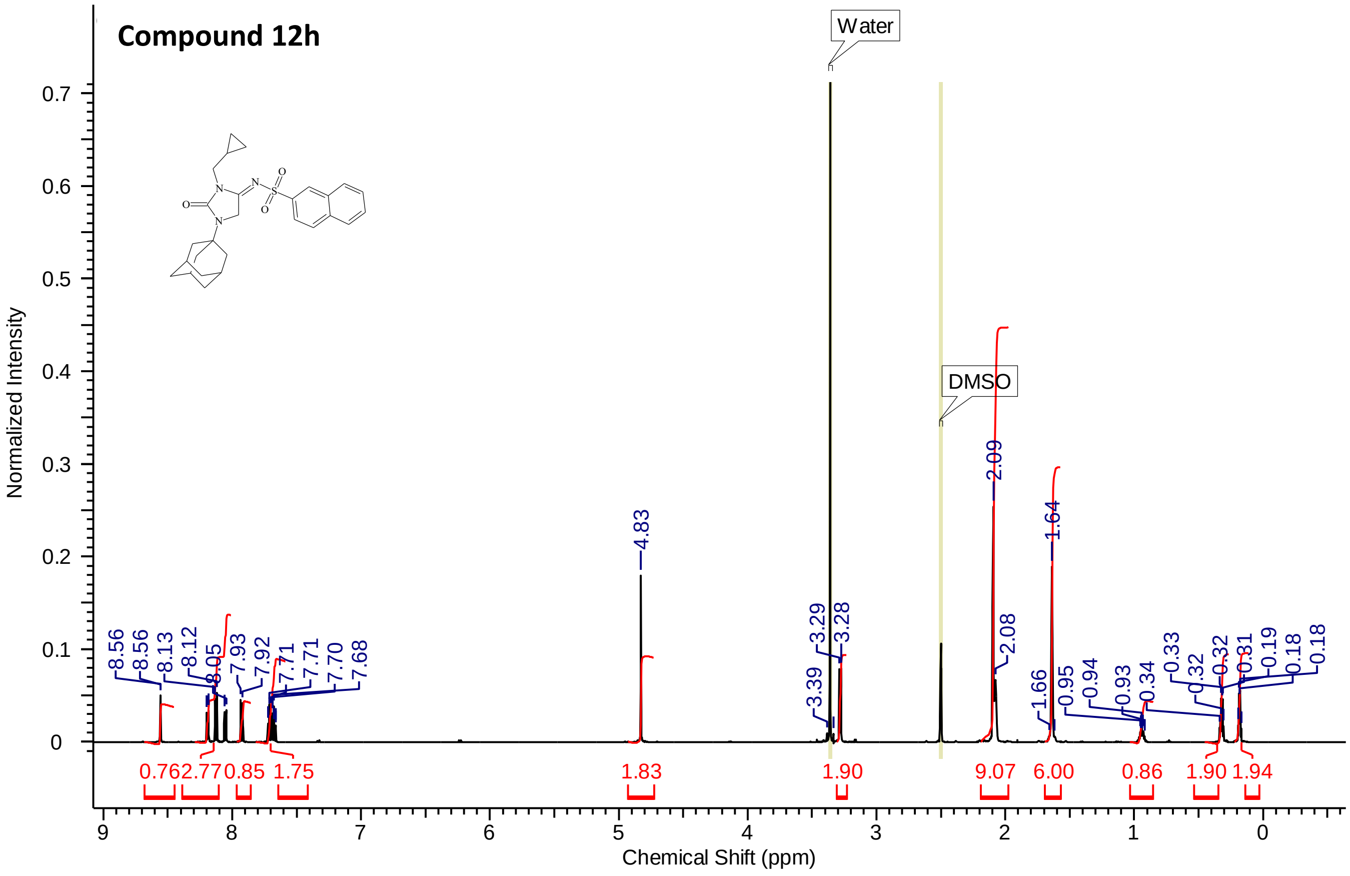
Compound 12h
Water
DMSO
Normalized Intensity
Chemical Shift (ppm)
8.56 8.56 8.13 8.12 8.05 7.93 7.92 7.71 7.71 7.70 7.68
4.83
3.39 3.29 3.28
2.09 2.08
1.66 1.64
0.95 0.94 0.93 0.34 0.33 0.32 0.32 0.31 0.19 0.18 0.18
0.76 2.77 0.85 1.75 1.83 1.90 9.07 6.00 0.86 1.90 1.94

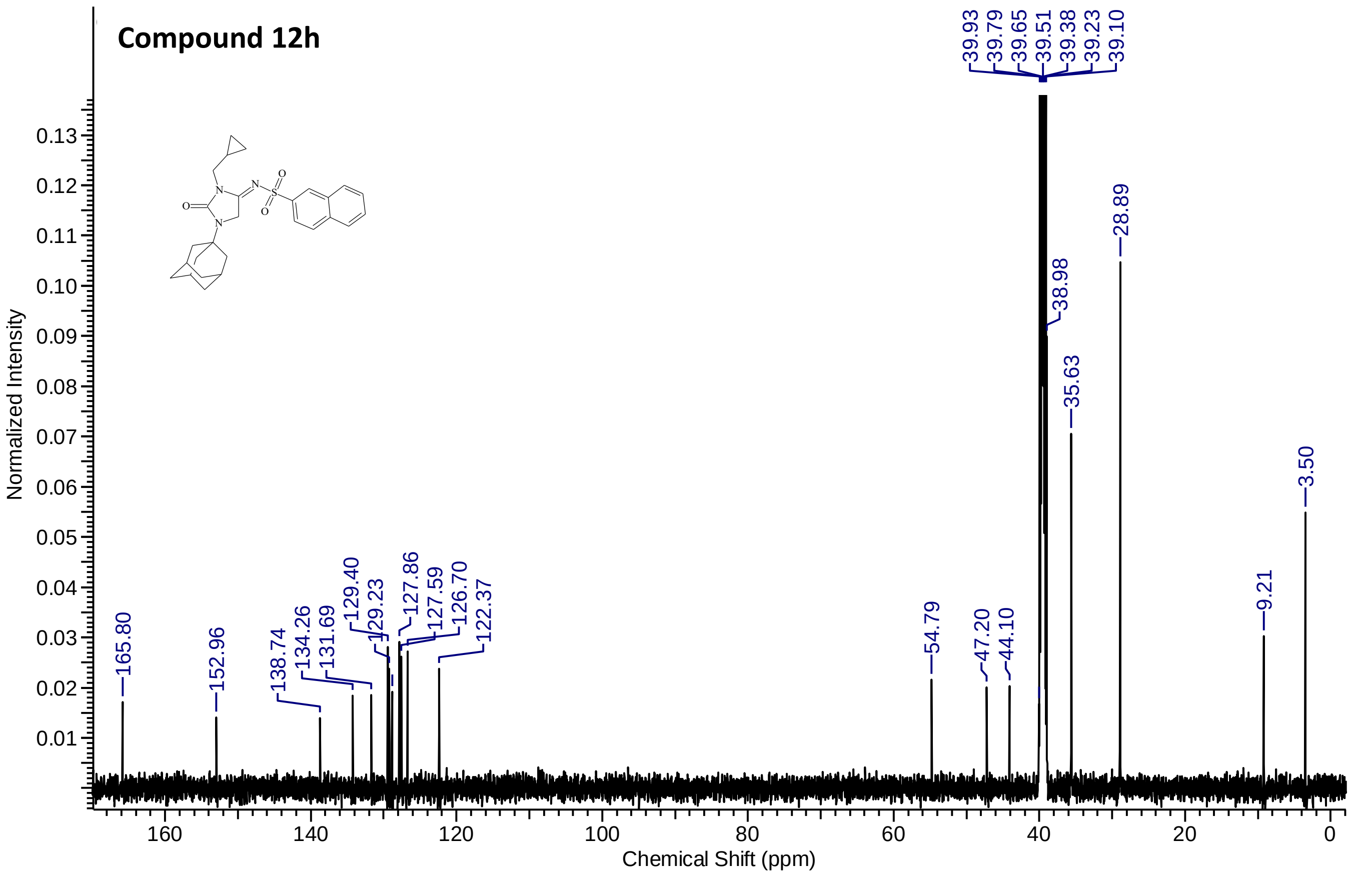
Compound 12h
Normalized Intensity
Chemical Shift (ppm)
39.93 39.79 39.65 39.51 39.38 39.23 39.10
165.80
152.96
138.74 134.26 131.69 129.40 129.23 127.86 127.59 126.70 122.37
54.79
47.20 44.10
38.98
35.63
28.89
9.21
3.50

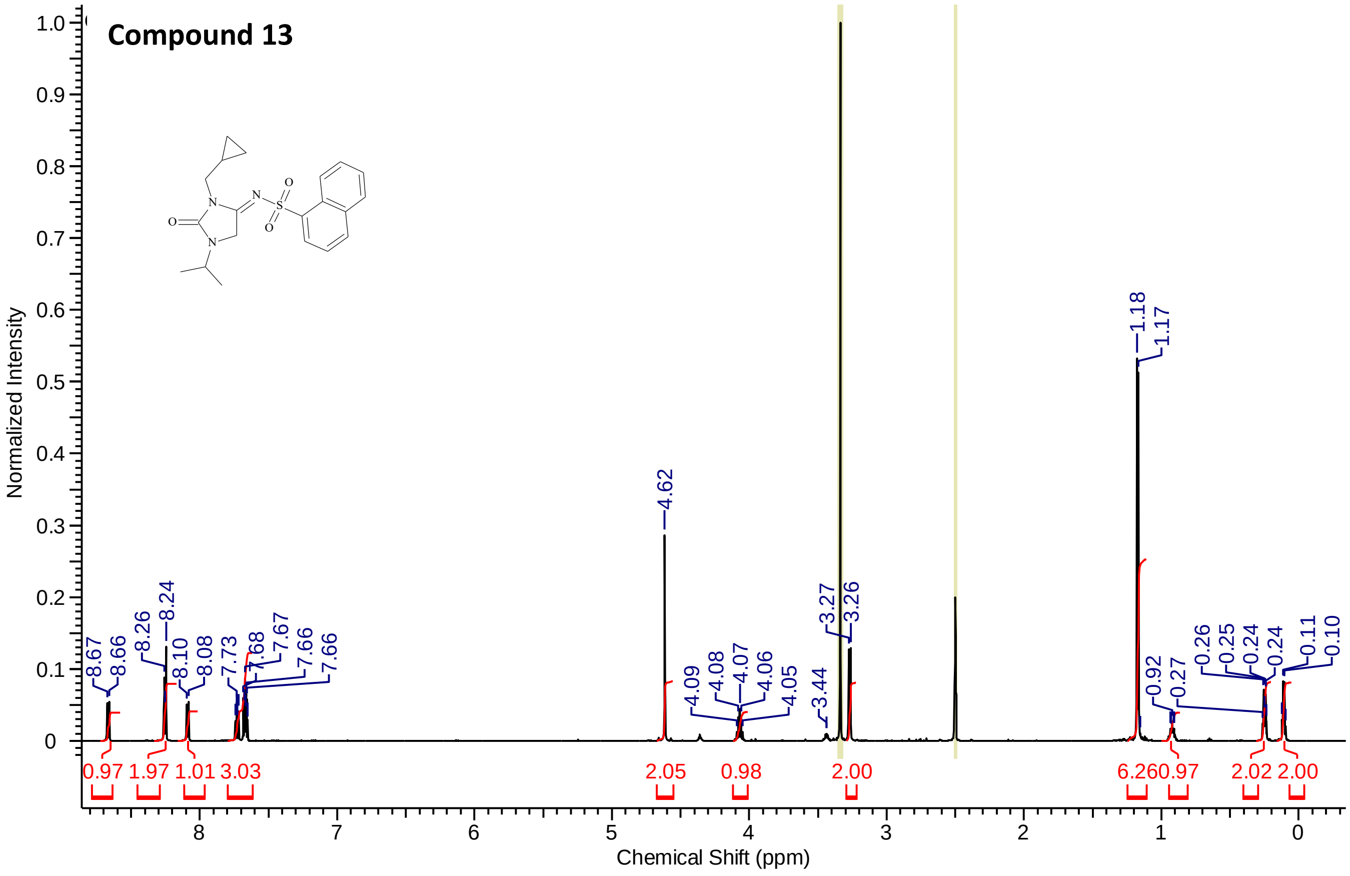
Compound 13
Normalized Intensity
Chemical Shift (ppm)
8.67 8.66 8.26 8.24 8.10 8.08 7.73 7.68 7.67 7.66 7.66
4.62
4.09 4.08 4.07 4.06 4.05
3.44
3.27 3.26
1.18 1.17
0.92 0.27 0.26 0.25 0.24 0.24 0.11 0.10
0.97 1.97 1.01 3.03 2.05 0.98 2.00 6.26 0.97 2.02 2.00

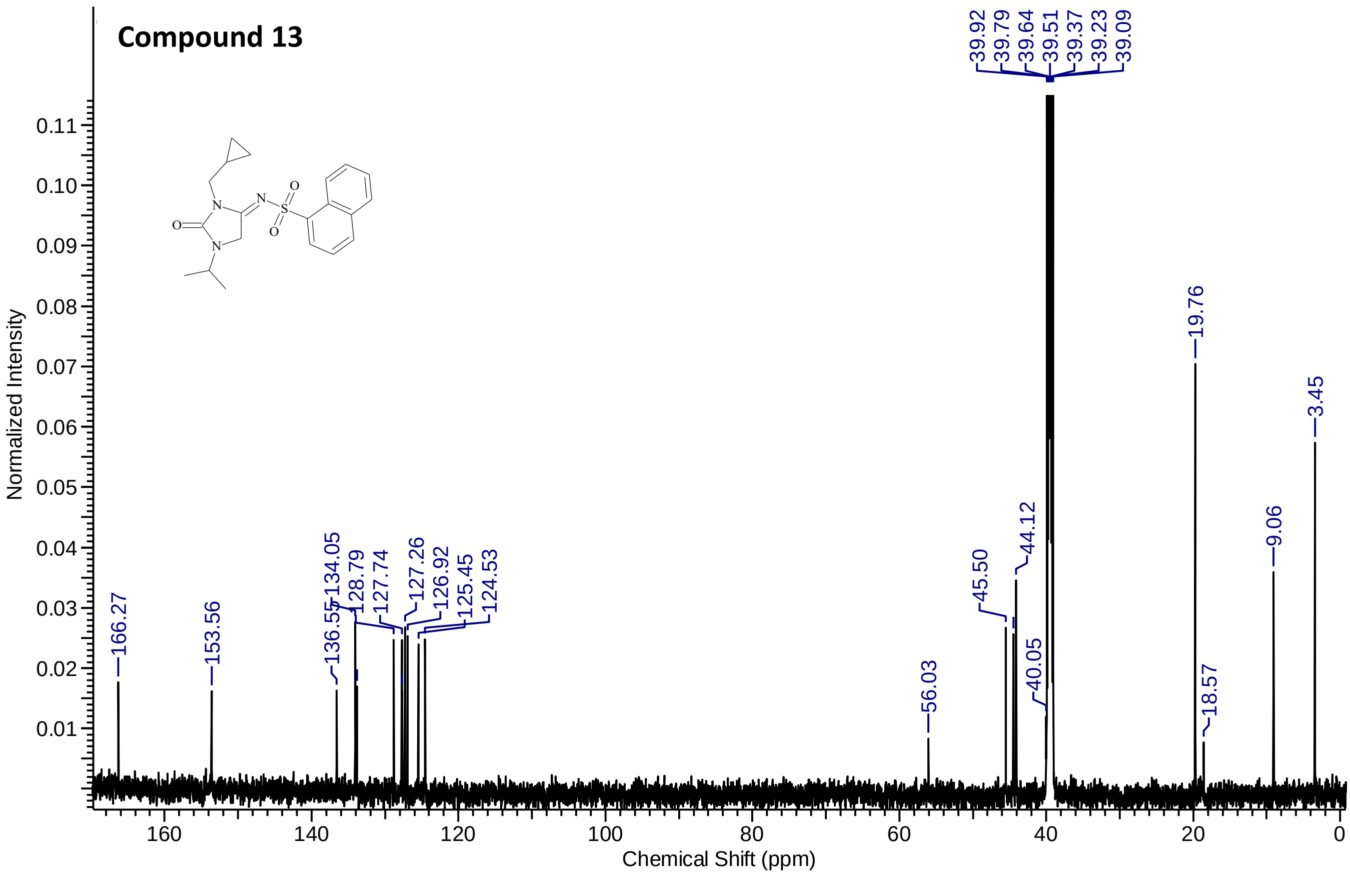
Compound 13
Normalized Intensity
Chemical Shift (ppm)
166.27
153.56
136.55 134.05 128.79 127.74 127.26 126.92 125.45 124.53
56.03
45.50 44.12 40.05
39.92 39.79 39.64 39.51 39.37 39.23 39.09
19.76 18.57
9.06
3.45

Compound 14

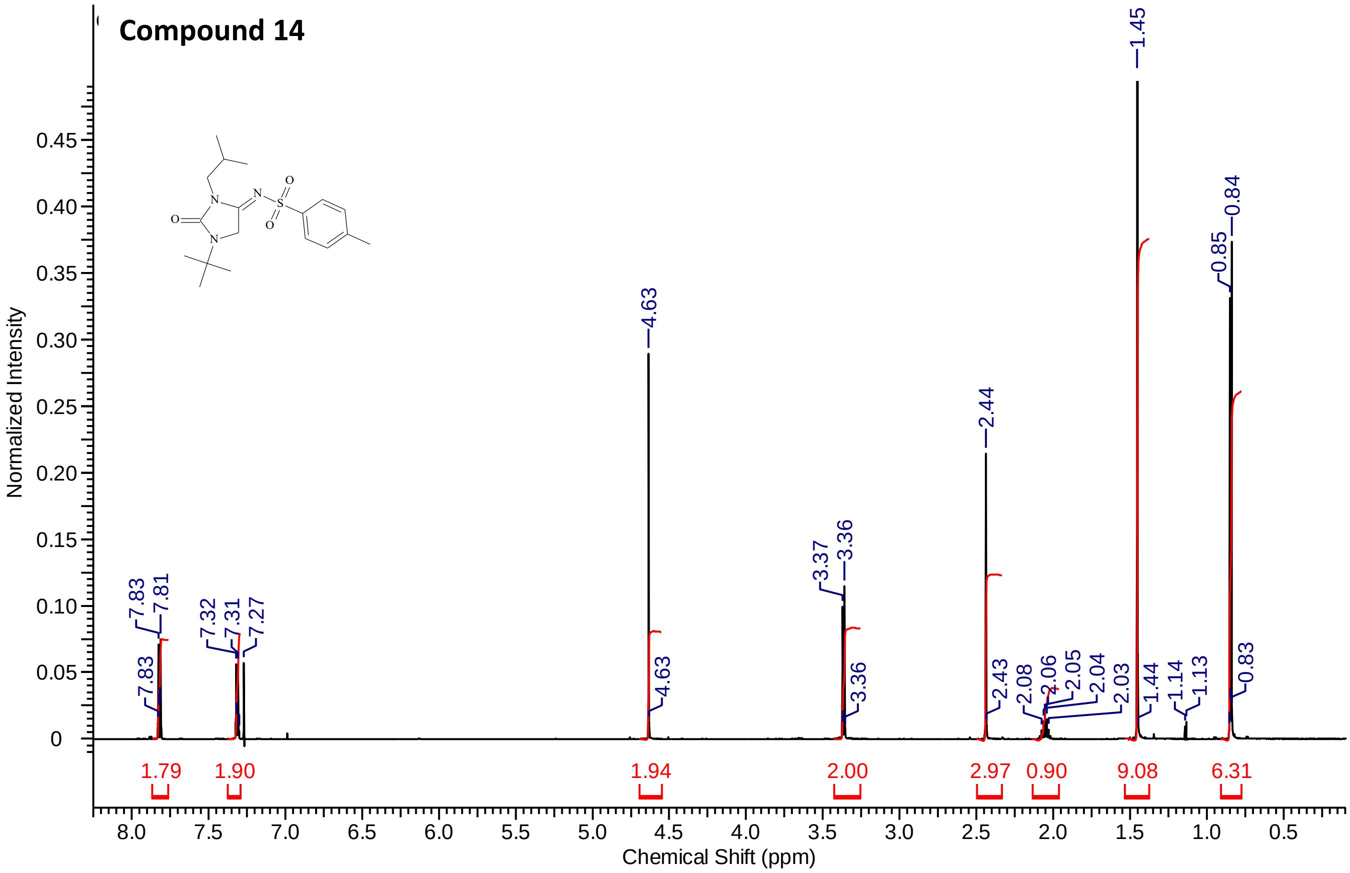

Compound 14

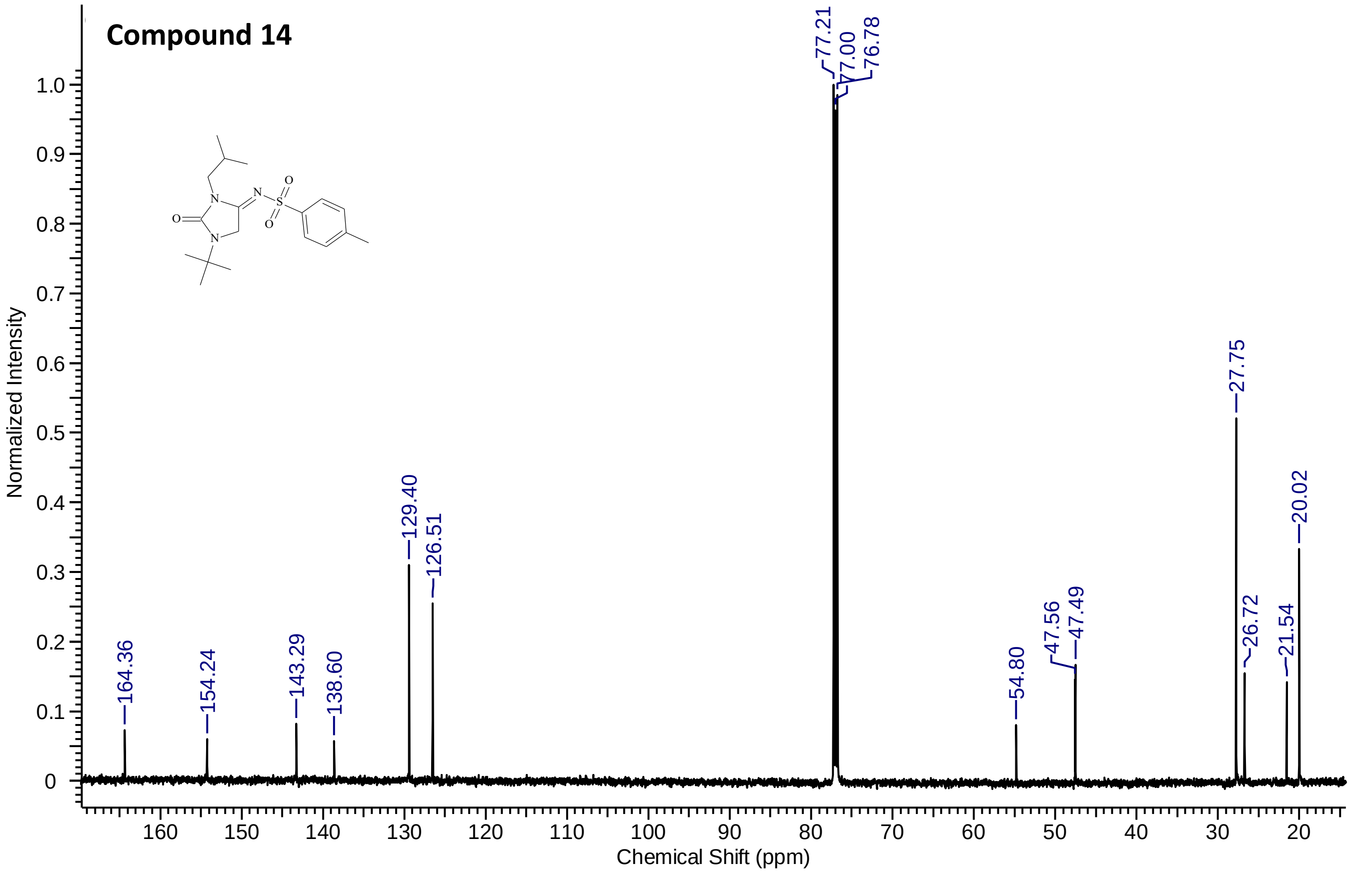

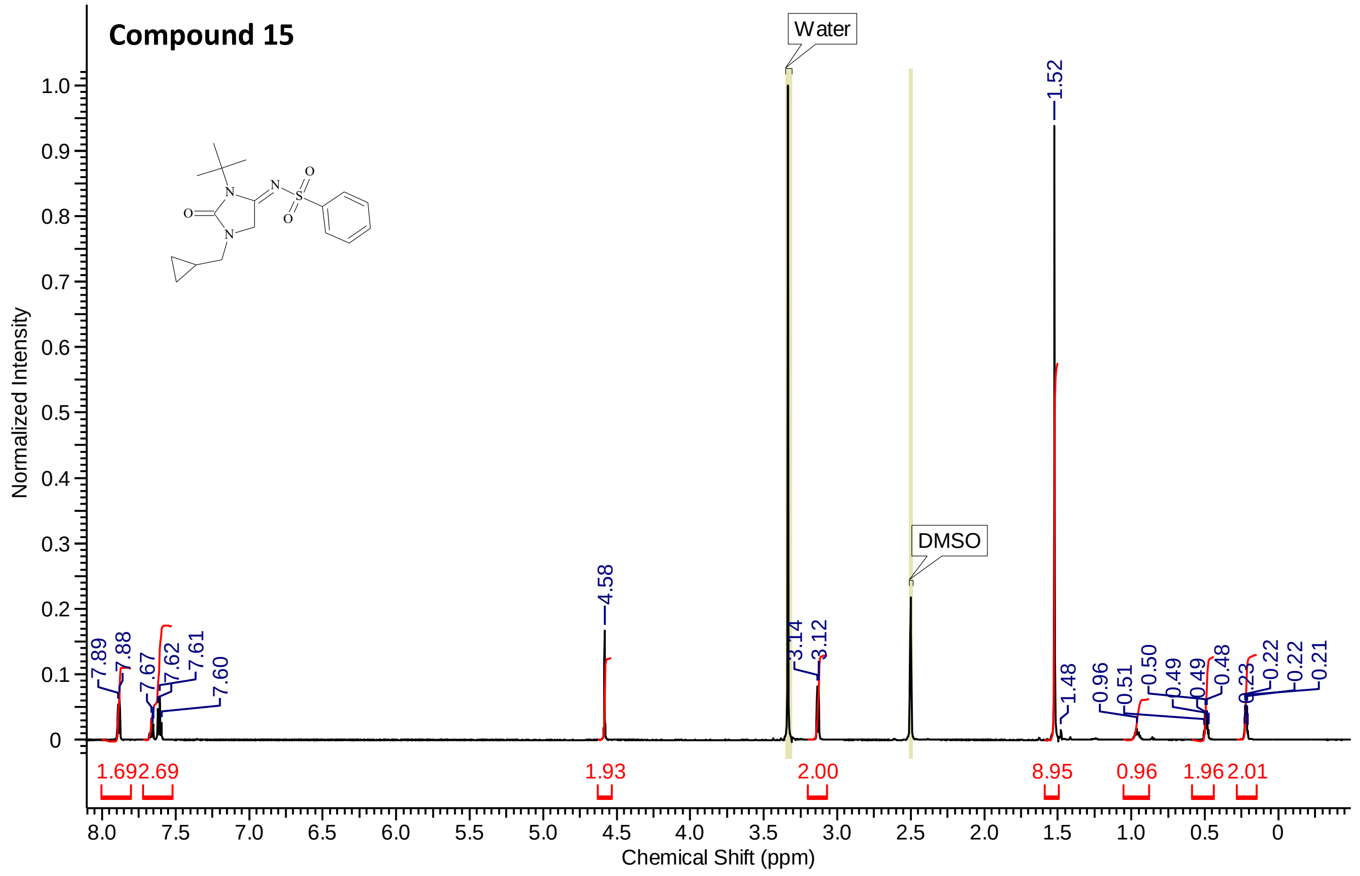
Compound 15
Water
DMSO
Normalized Intensity
Chemical Shift (ppm)
7.89
7.88
7.67
7.62
7.61
7.60
4.58
3.14
3.12
1.52
1.48
0.96
0.51
0.50
0.49
0.49
0.48
0.23
0.22
0.22
0.21
1.69
2.69
1.93
2.00
8.95
0.96
1.96
2.01

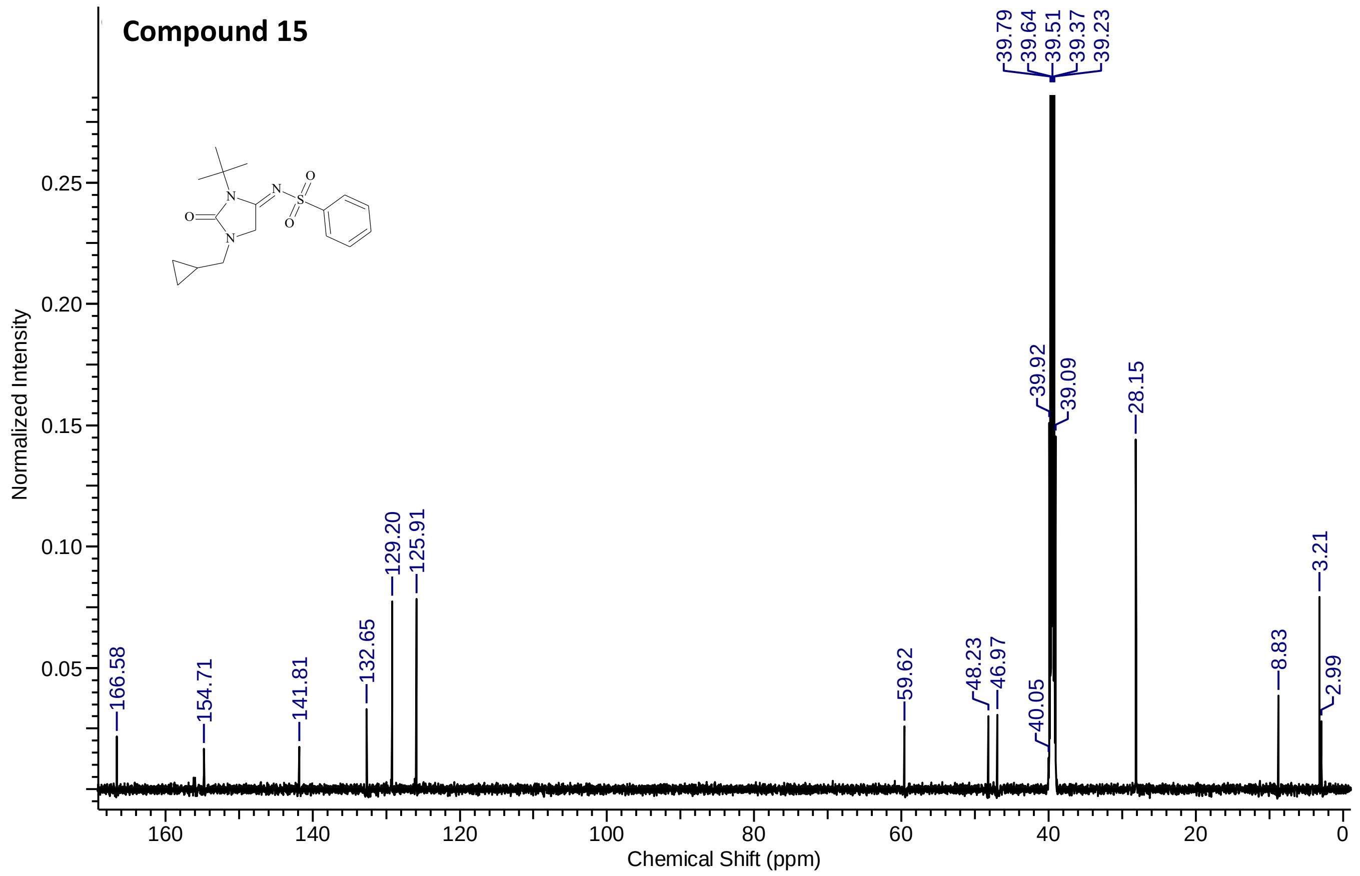
Compound 15
Normalized Intensity
Chemical Shift (ppm)
166.58
154.71
141.81
132.65
129.20
125.91
59.62
48.23
46.97
40.05
39.92
39.79
39.64
39.51
39.37
39.23
39.09
28.15
8.83
3.21
2.99
